# Computational NMR of Carbohydrates: Theoretical Background, Applications, and Perspectives

**DOI:** 10.3390/molecules26092450

**Published:** 2021-04-22

**Authors:** Leonid B. Krivdin

**Affiliations:** A. E. Favorsky Irkutsk Institute of Chemistry, Siberian Branch of the Russian Academy of Sciences, Favorsky St. 1, 664033 Irkutsk, Russia; krivdin55@gmail.com

**Keywords:** computational NMR, chemical shift, spin-spin coupling constant, carbohydrates

## Abstract

This review is written amid a marked progress in the calculation of NMR parameters of carbohydrates substantiated by a vast amount of experimental data coming from several laboratories worldwide. By no means are we trying to cover in the present compilation a huge amount of all available data. The main idea of the present review was only to outline general trends and perspectives in this dynamically developing area on the background of a marked progress in theoretical and computational NMR. Presented material is arranged in three basic sections: (1)—a brief theoretical introduction; (2)—applications and perspectives in computational NMR of monosaccharides; and (3)—calculation of NMR chemical shifts and spin-spin coupling constants of di- and polysaccharides.

## 1. Introduction

**Figure 1:**
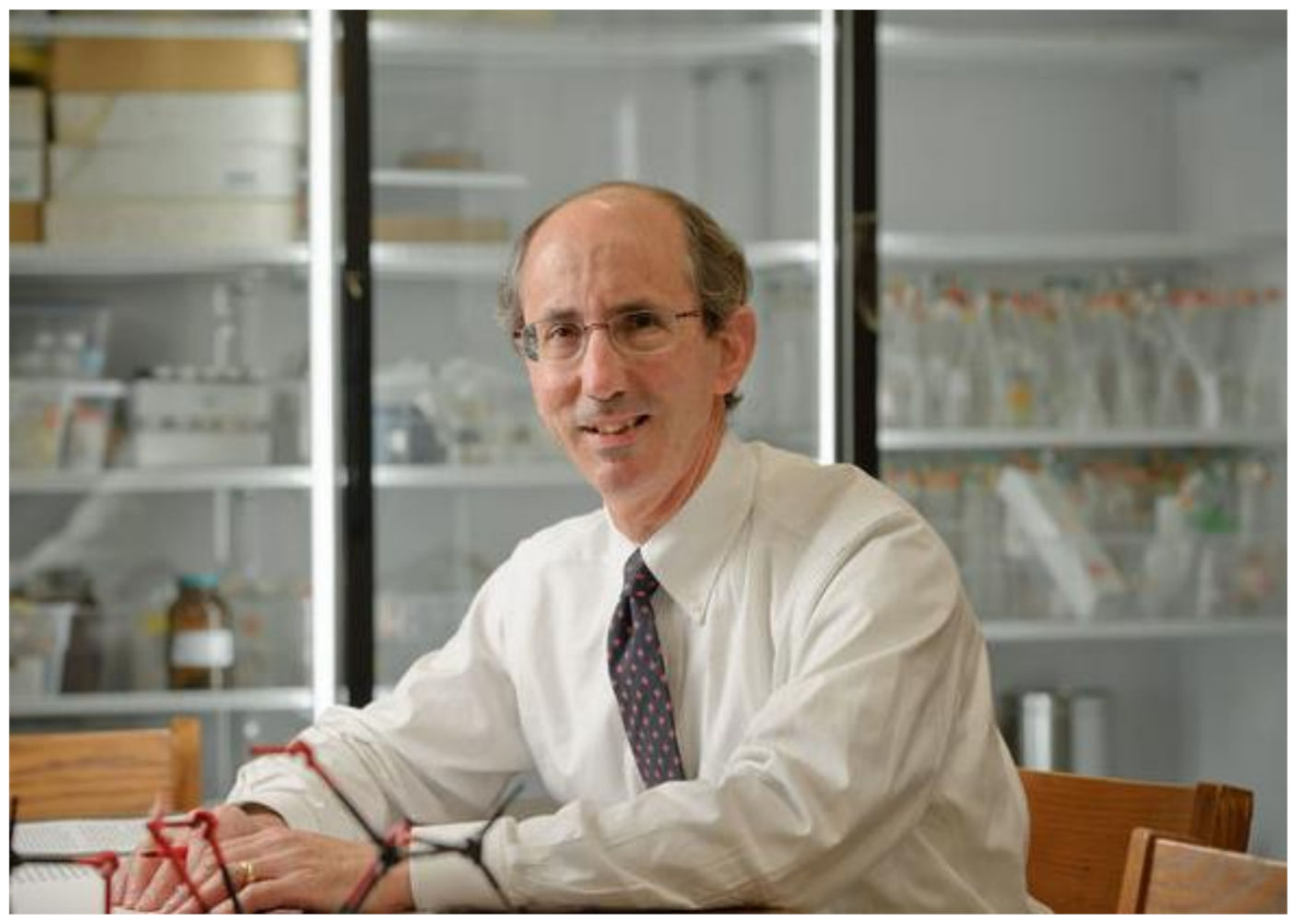
Professor Anthony S. Serianni, University of Notre Dame, USA. Adopted from the official site of the University of Notre Dame.

A major breakthrough in the stereochemical studies of carbohydrates by means of computational NMR has mainly been achieved by Serianni (see Figure 1 adopted from the official site of the University of Notre Dame) and coworkers based on a vast amount of original publications and comprehensive reviews. As a result of those numerous fundamental studies, it was demonstrated that stereochemical analysis of carbohydrates could mostly be performed by using computational and experimental ^1^H- and ^13^C-NMR chemical shifts and spin-spin coupling constants in view of their marked stereochemical dependences.

As an example, ^3^*J*_H,H_ values, measured experimentally and calculated theoretically, exercised a profound role in the assigning of the preferred conformations of furanose and pyranose rings. This topic is well covered in recent books, book chapters, and numerous reviews from Serianni’s group (see most recent reviews by Klepach et al. [1] and Hadad et al. [2]) together with several early reviews from the same group [3,4,5,6] and in a comprehensive review by Toukach and Ananikov [7]. Those reviews, providing a new guide in the fundamental factors controlling molecular recognition and catalysis in biochemical systems, are based on a great number of original research papers which are partly discussed and referenced below.

By no means are we trying to cover in the present compilation a huge amount of data coming mostly from Serianni’s laboratories. The main idea of the present review was to outline the main trends and perspectives in this dynamically developing area in view of a marked progress of computational NMR [8], applied to the calculation of chemical shifts and spin-spin coupling constants, primarily those involving proton [9,10,11] and carbon [12,13,14] nuclei.

## 2. Theoretical Background

### 2.1. Levels of Theory

Theoretical aspects of the calculation of two basic NMR parameters, chemical shifts and spin-spin coupling constants, are well covered in a fundamental handbook of molecular electromagnetism by Stephan Sauer [8] and in a number of original reviews on theoretical and methodological aspects of general computational NMR [15,16,17,18,19,20,21,22,23,24,25,26]. Usually, practical NMR spectroscopists prefer to deal with “chemical shift” rather than “shielding constant”, the later defined as “the difference between the external magnetic flux density and the local magnetic flux density at a resonating nucleus affected by the neighboring electrons divided by the external flux density” (IUPAC). Further in the review, it is the former which will be utilized as much as possible.

A number of recent comprehensive reviews concentrated on particular computational aspects for the less common nuclei (i.e., those excluding ^1^H and ^13^C)—namely, nitrogen [27], fluorine [28], silicon [29], phosphorus [30,31], selenium [32,33,34], and heavy nuclei [34,35,36]. Theoretical calculations of NMR parameters are nowadays performed at either non-empirical (ab initio) level with taking into account electronic correlation in an explicit way or within the density functional theory (DFT) including electronic correlation effects inexplicitly.

For the DFT level, the most “trustful” (in my opinion) functionals and basis sets are listed in Section 2.1.2. For the non-empirical level, the most convenient results are achieved with the coupled cluster methods for chemical shifts and Sauers’ polarization propagator methods for spin-spin coupling constants, as discussed in Section 2.1.1. Herewith, we will briefly concentrate on theoretical and computational aspects of the calculation of NMR chemical shifts and spin-spin coupling constants at both levels of theory leaving deeper theoretical insight for a more specialized consideration.

#### 2.1.1. Non-Empirical Level

Nowadays, calculations of NMR chemical shifts and spin-spin coupling constants at the non-empirical level are markedly increasing and becoming more and more common in the practice of computational NMR. The Restricted Hartree-Fock (RHF) or Random Phase Approximation (RPA) level and a suite of correlated post Hartree Fock methods are now realized in a number of quantum-chemical packages like very popular Gaussian [37].

For the calculation of NMR chemical shifts at the non-empirical level, a number of correlated methods like second-order Møller-Plesset perturbation theory (MP2), Coupled Cluster Singles and Doubles (CCSD) and Coupled Cluster Singles and Doubles with Perturbative Triples Corrections, CCSD(T) are widely used throughout. Higher-level Coupled Cluster Single-Double and Triple corrections (CCSDT) and Coupled Cluster Iterative Triples model (CC3) methods showing very promising but enormously expensive results are rather far from their practical application. Indeed, CCSD, CCSD(T), CCSDT, and especially CC3 methods are characterized by a high degree of electron correlation taken into account, but their practical applications are, at least nowadays, computationally limited to very small molecules due to their enormously large computational demands.

In the basic publication by Auer and Gauss [38] dealing with the high-level calculations of indirect spin-spin coupling constants, the effect of triple excitations in a coupled-cluster was investigated. The coupled-cluster singles and doubles CCSD calculations were augmented by a perturbative treatment at the CCSD(T), CCSDT, and CC3 levels. Though triple excitation effects were found to be in most cases not particularly pronounced, it was demonstrated that among the approximate schemes for handling triples, only the CC3 model with no orbital relaxation included unrelaxed CC3 provided the adequate results. The otherwise successful CCSD(T) approach appeared to either significantly overestimate triple excitation effects or to yield corrections with the wrong sign in comparison to CCSDT.

In a much more recent publication on this point [39], the first analytical implementation of CC3 s derivatives using the spin-unrestricted approach was introduced. This allowed the calculation of nuclear spin-spin coupling constants at the CC3 level of theory in a fully analytical manner. The CC3 results for a number of small molecules and their fluorine substituted derivatives involved in that study were compared with the corresponding CCSD results obtained using some specialized basis sets. Calculations at the CCSDT level indicated that the most important contributions arising from connected triple excitations in the coupled cluster expansion should be accounted for at the CC3 level.

For the calculation of spin-spin coupling constants at the non-empirical level, very promising in terms of the degree of electron correlation taken into account versus computational effort are Sauer’s suite of methods. Among those are the Second-Order Polarization Propagator Approach within the second-order Møller-Plesset perturbation theory, SOPPA(MP2), Second-Order Polarization Propagator Approach in combination with the second-order approximate Coupled Cluster to second order, SOPPA(CC2), and Second-Order Polarization Propagator Approach in combination with Coupled Cluster Singles and Doubles, SOPPA(CCSD). For general references on the SOPPA-based methods for the calculation of spin-spin coupling constants, see basic publications [40,41,42,43].

In this regard, one of the reviewers commented that “for shielding tensors, CCSD and CCSD(T) have been implemented in the GIAO framework as analytical second derivatives. The SOPPA approach is an alternative path to properties introducing further approximations. However, also for conventional CCSD analytical derivatives have been implemented for coupling constants without the need for a polarization propagator treatment. CCSD(T) on the other hand is not used, as the triples correction introduces the triplet instability problem can yield unphysical contributions. While one could argue whether this is “too theoretical” for someone who just wants to apply a method, it would inform readers about potential catastrophic failures and give due credit to all scientists who helped to develop methods like higher order correlation treatments and analytical derivative implementations”.

In the forthcoming very recent theoretical article [44], the Random Phase Approximation Doubles, RPA(D), and Highest Random Phase Approximation Doubles, HRPA(D), models for the calculation of linear response functions were introduced. The performance of the RPA(D) and HRPA(D) models was compared to the performance of the established RPA, HRPA, and SOPPA methods in the calculation of spin-spin coupling constants using the CCSD level as a reference. The doubles correction offered a significant improvement on both the RPA and HRPA models; however, the improvement was more dramatic in the case of the RPA approximation. For all coupling types investigated in that study, the results obtained using the HRPA(D) model were comparable in accuracy to those given by the SOPPA level. The RPA(D) model, while of slightly lower accuracy compared to the CCSD than HRPA(D), offered computational times of only approximately 25% of those required for SOPPA, as was exemplified two years later by the same principal authors for carbon-carbon coupling constants [45].

For general references on those methods involving general theoretical aspects and their practical implementation, see most comprehensive review by Sauer [viii] and references given therein.

It should be emphasized that the non-empirical calculations of NMR chemical shifts and spin-spin coupling constants in the medium-sized organic molecules (and even those being larger) are becoming more and more common in chemical practice. Undoubtedly, in a very near future along with a general progress in computational software and hardware, they will dominate over the traditionally employed DFT calculations, at least for small and the medium-sized molecules.

#### 2.1.2. DFT Level

Nowadays, calculations of NMR chemical shifts and spin-spin coupling constants in carbohydrates and larger biological species are performed mostly within the DFT framework, in contrast to the non-empirical computations applied to much smaller molecules. This is not surprising in view of the fact that at the DFT level involving electron correlation in an implicit way, such calculations are much more economic (in the sense of computing resources), as compared to the non-empirical methods, the latter taking into account electron correlation effects explicitly. However, it should be kept in mind that results of the DFT calculations drastically depend on the choice of a particular functional and, to a lesser extent, on the use of a particular basis set.

Among dozens and dozens of various local spin-density, exchange, correlation and exchange-correlation functionals used for the calculation of NMR chemical shifts and spin-spin coupling constants, the following ones are most popular among practical computational chemists:

- B3LYP—the most common three-parameter hybrid functional of Becke (B3) [46] in combination with the correlation functional of Lee, Yang and Parr (LYP) [47];
- BHLYP—Becke’s “half and half” HF/DFT hybrid exchange functional (BH) [48] in combination with the Lee, Yang, and Parr correlation functional (LYP) [49];
- OLYP—Handy and Cohen’s hybrid functional (OPTX) [48] in combination with Lee, Yang and Parr’s correlation functional (LYP) [48];
- OPBE—Handy and Cohen’s hybrid functional (OPTX) [49] in combination with the PBE correlation functional (PBEc) [50];
- OPW91—Handy and Cohen’s hybrid functional (OPTX) [49] in combination with the PW91 correlation functional [51];
- PBE0—the generalized gradient functional of Perdew, Burke and Ernzerhof [50] with a predetermined amount of exact exchange [52];
- KT2 and KT3—generalized gradient exchange-correlation functional of Keal and Tozer (KT2) [53] and that with the gradient-corrected exchange and correlation terms (KT3) [54].

On the other hand, the following basis sets are currently used for the calculations of NMR chemical shifts and spin-spin coupling constants (for the choice of a particular basis set, see Basis Set Exchange (BSE) database [55]):

- Pople’s 6-31G and 6-311G family of functionals with or without diffuse functions and different sets of polarization functions;
- Dunning’s cc-pVnZ (*n* = D, T, Q, 5) correlation-consistent basis sets and their augmented (diffuse) versions (aug-cc-pVnZ);
- Dunning’s cc-pCVnZ (*n* = D, T, Q, 5) correlation-consistent core-valence basis sets and their augmented versions (aug-cc-pCVnZ);
- Sauer’s augmented correlation-consistent basis sets aug-cc-pVTZ-J;
- Sauer’s correlation-consistent 6-31G-J and 6-311G-J basis sets;
- Jensen’s polarized-consistent basis sets (pc-n, *n* = 0−4) and their augmented versions (aug-pc-n);
- Jensen’s pc-n-based basis sets (pcS-n and pcJ-n) and their augmented versions (aug-pcS-n and aug-pcJ-n) used accordingly for the calculations of NMR chemical shifts and, on the other hand, spin-spin coupling constants;
- Jensen’s segment contracted pcS-n basis sets (pcSseg-n, *n* = 0, 4) and their augmented versions (aug-pcSseg-n) used for the calculations of NMR chemical shifts;
- Four different groups of “relativistic” Dyall’s basis sets of double-, triple- and quadruple-zeta quality, namely, valence (dyall.vXz), core-valence (dyall.cvXz), diffuse valence (dyall.avXz) and diffuse core-valence (dyall.acvXz), X = 2, 3 and 4.

The vitally important aspects of calculation of NMR parameters dealing with a proper choice of a functional and a basis set are discussed in much detail in a recent comprehensive review by Iron [56] providing insight into the factors affecting the accuracy of NMR calculations at the DFT level.

#### 2.1.3. DP4 Analysis

It is well known that stereochemical study of complex biological molecules requires a detailed knowledge of their absolute configuration together with the preferred conformations, which are nowadays being mostly established by means of the DP4 method [57] together with its DP4+ modification, the later developed in the paper by Grimblat and coworkers [58].

In the first paper, a new probability measure for assigning a probability to each candidate structure was introduced and tested to distinguish for up to 64 possible diastereoisomers of 117 different molecules with using NMR shifts obtained in rapid and computationally inexpensive single-point calculations on molecular mechanics geometries. It was shown that a DP4 probability analysis based on the errors in each ^13^C or ^1^H-NMR chemical shift was significantly more successful at making correct assignments with high confidence than are probabilities based on the correlation coefficient and mean absolute error parameters. As an example, the application of the DP4 method was illustrated by assigning the stereochemistry or structure of 21 natural products that were originally misassigned.

In the second paper, in order to improve the performance of the DP4 method, a modified version, DP4+, was introduced, whose main differences from the original DP4 method were the inclusion of unscaled data and the use of higher levels of theory for the NMR computational procedure. The DP4+ probability *P_i_* of each of *m* candidate diastereomers is evaluated by using the equation:

(1) $$P_{i}=\frac{\prod_{k=1}^{N}\left[{1-T}_{s}^{\nu }\left(\frac{e_{s,k}^{i}}{\sigma_{s}}\right)\right]\left[{1-T}_{u-spx}^{\nu }\left(\frac{e_{u,k}^{i}{-\mu }_{u-\mathrm{spx}}}{\sigma_{u-\mathrm{spx}}}\right)\right]}{\sum_{j=1}^{m}\prod_{k=1}^{N}\left[{1-T}_{s}^{\nu }\left(\frac{e_{s,k}^{i}}{\sigma_{s}}\right)\right]\left[{1-T}_{u-spx}^{\nu }\left(\frac{e_{u,k}^{i}{-\mu }_{u-\mathrm{spx}}}{\sigma_{u-\mathrm{spx}}}\right)\right]}$$

where *T_s_^ν^*, and *T_u-spx_^ν^* are the standard cumulative *t*-distribution function with *ν* degrees of freedom centered on average error *μ* and variance *σ* corresponding to the scaled and unscaled hybridization of the carbon atoms. Accordingly, scaled and unscaled errors *e_s_* and accordingly, *e_u_* for the nucleus *k* could be calculated as *e_s,u_* = *δ_s,u_*−*δ_exp_*. With these modifications, a significant improvement in the overall performance of DP4 was achieved, as was illustrated for the establishing the stereochemistry of 48 challenging isomeric compounds [58].

**Figure 2:**
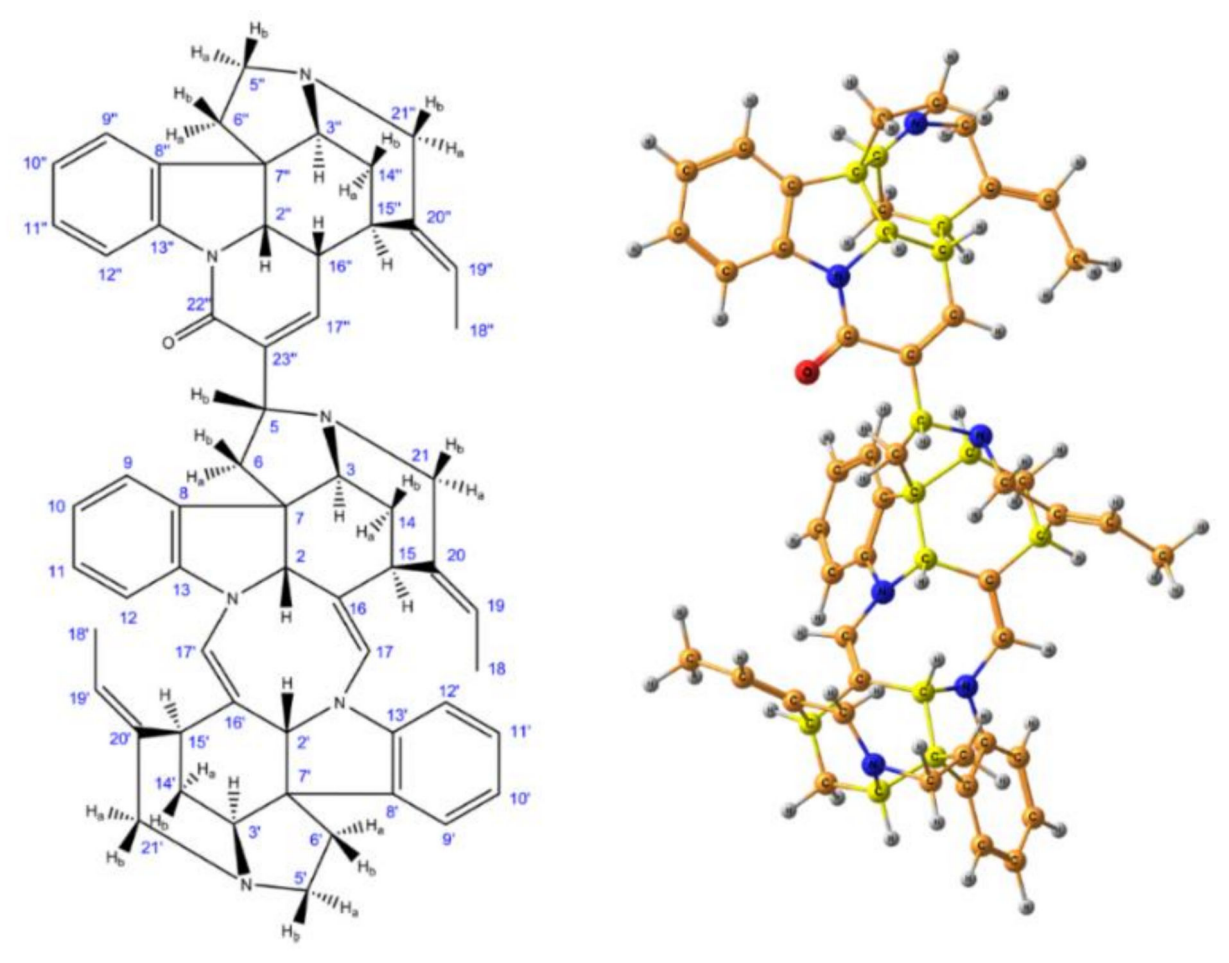
Enumeration of atoms (**left**) and stereochemical structure (**right**) of strychnohexamine. Element colors are as follows: protons—gray, carbons—dark yellow, nitrogens—blue, oxygen—red, while the chiral carbons are given in bright yellow. Reproduced from Semenov et al. [59] with the permission of John Wiley and Sons.

**Figure 3:**
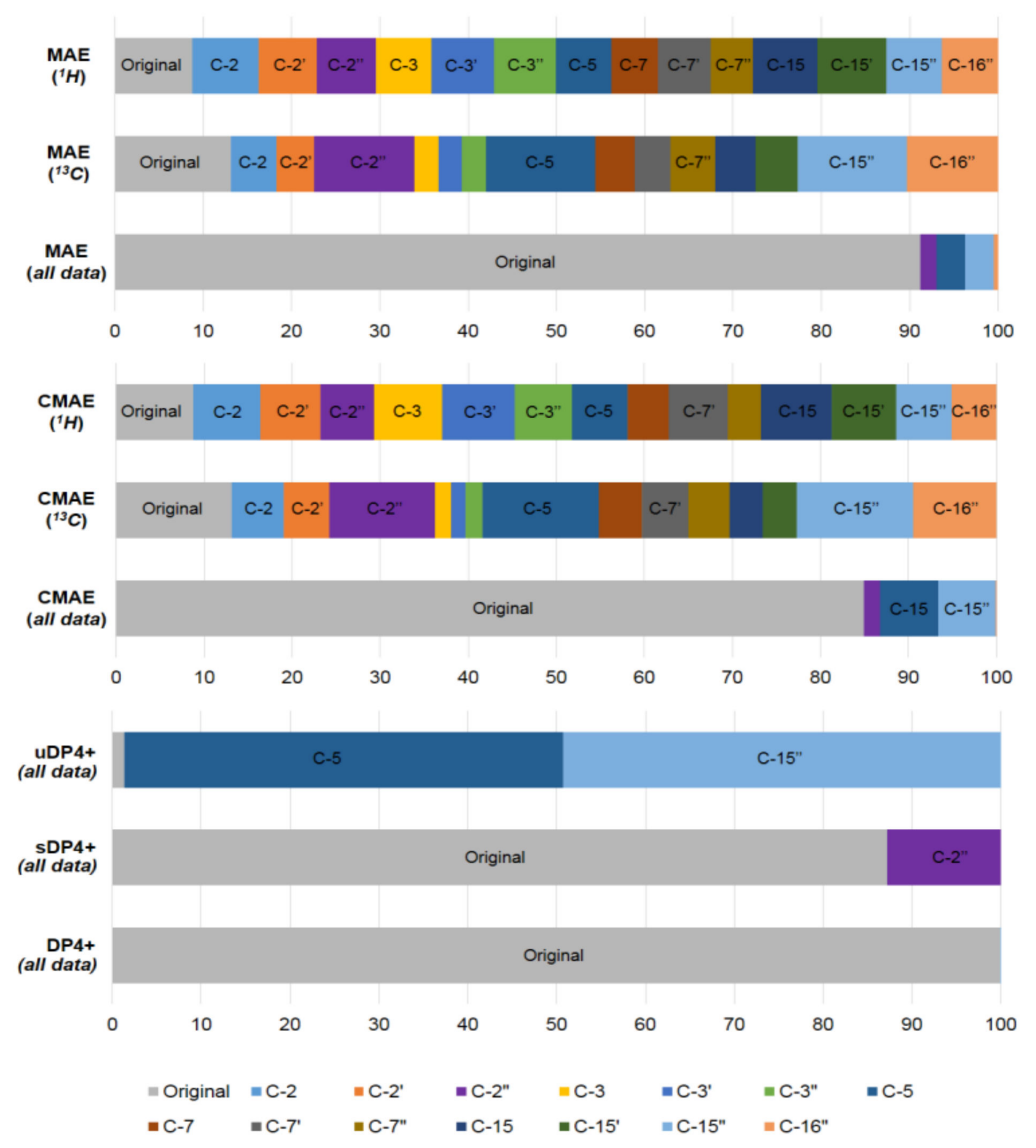
Graphs of the DP4+ probability distribution for each diastereomer of strychnohexamine (**1**) together with the probabilities derived from the performed correlation analysis and the values of the resulting MAE and CMAE. Reproduced from Semenov et al. [59] with the permission of John Wiley and Sons.

As a further example of the DP4+ application, in a very recent paper by Semenov and Krivdin [59] the graphs of the probabilities for each candidate diastereomer of strychnohexamine **1** (shown in Figure 2) were presented as illustrated in Figure 3. The latter were assigned based on the DP4+ results together with corresponding data provided by the Mean Absolute Error (MAE) and Corrected Mean Absolute Error (CMAE) analysis. As followed from the performed DP4+ analysis, the prevailing diastereomer of strychnohexamine was characterized by a 99.93% probability supporting its original structure.

#### 2.1.4. Locally Dense Basis Set Scheme

Within the Locally Dense Basis Set (LDBS) scheme applied for the calculation of chemical shieldings (chemical shifts) [60,61,62] and spin-spin coupling constants [63,64], a large high-quality basis set is placed on a particular atom (or group of atoms) of interest while much smaller basis sets are employed elsewhere in the molecule which results in a dramatic decrease of computational cost. In other words, within the LDBS approach, large basis sets are used on a particular atom or a small group of atoms of interest while much smaller basis sets are used in the rest of molecule, which preserves high accuracy of calculation at much less computational efforts.

In the earlier publications, the LDBS scheme was successfully applied for the calculation of chemical shifts at the Hartree-Fock and DFT levels in a number of simple benchmark molecules [60,61,62], in relatively large molecular systems [65], and even in the molecules of biological interest like dipeptides [66].

**Figure 4:**
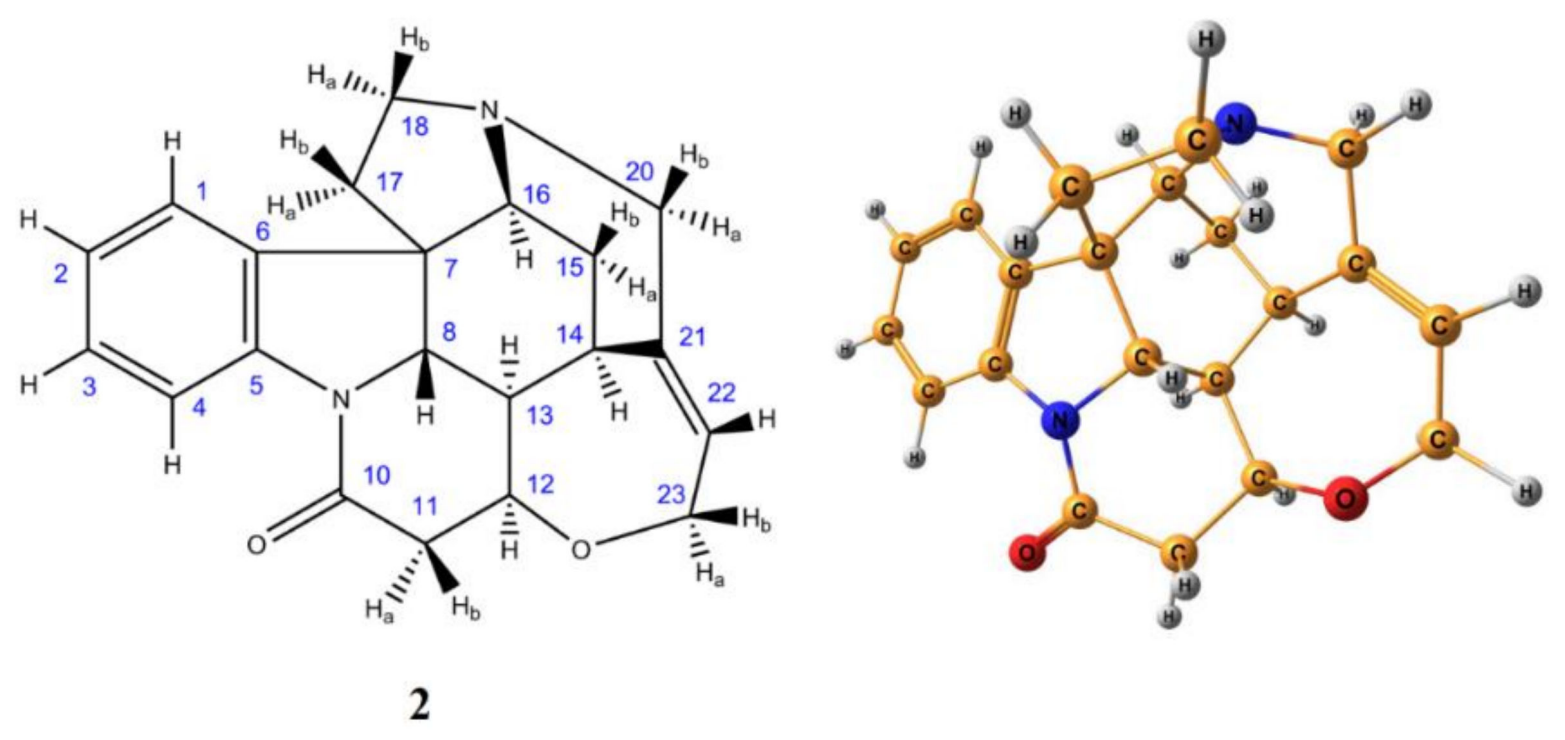
Enumeration of atoms (**left**) and stereochemical structure (**right**) of strychnine. Element colors are as follows: protons—gray, carbons—dark yellow, nitrogens—blue, oxygen—red. Reproduced from Semenov and Krivdin [67] with the permission of John Wiley and Sons.

**Figure 5:**
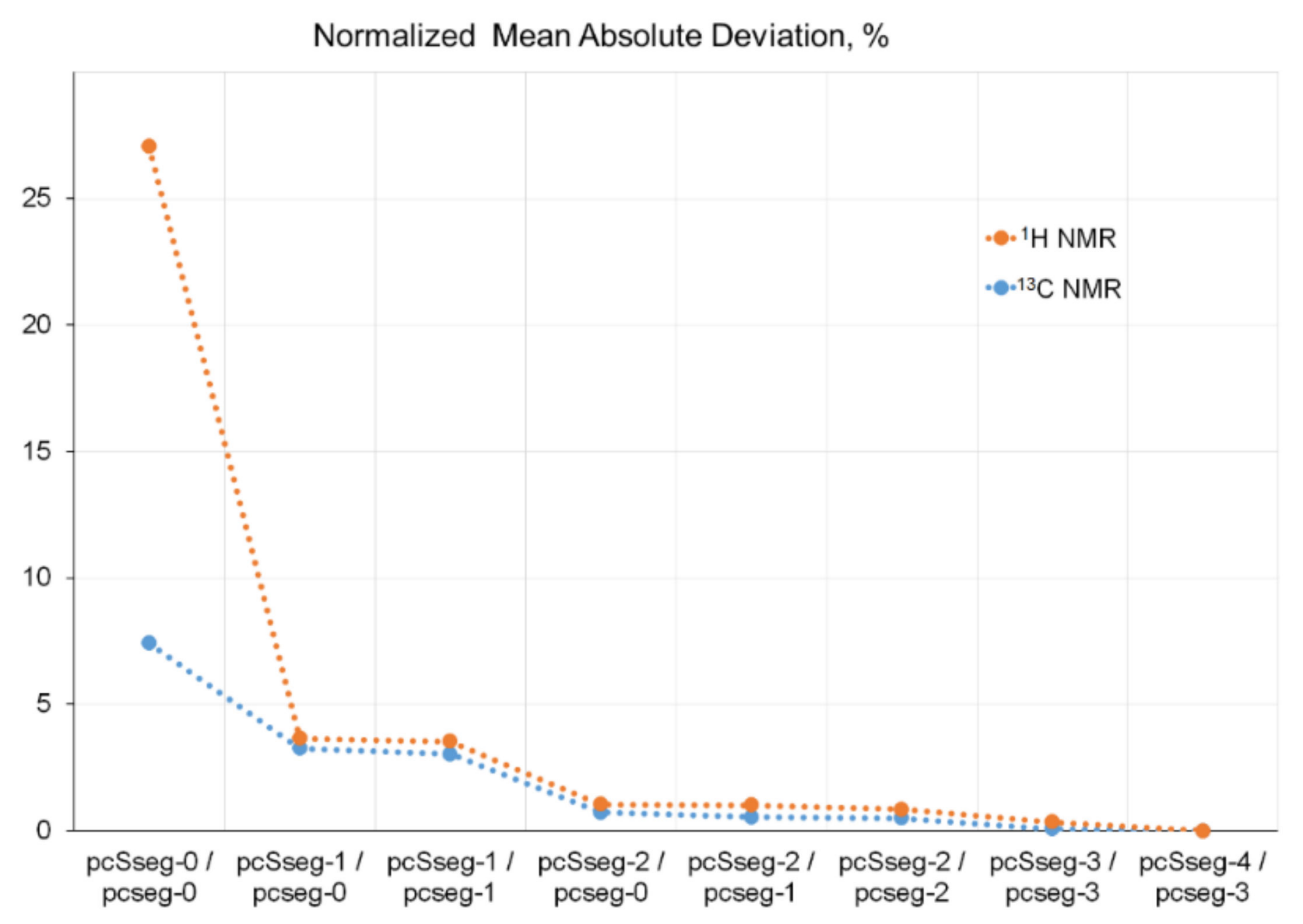
Normalized mean absolute deviations of ^1^H and ^13^C-NMR shielding constants of strychnine (**2**), calculated using different locally dense basis set schemes based on segmented Jensen’s basis sets. Reproduced from Semenov and Krivdin [67] with the permission of John Wiley and Sons.

**Figure 6:**
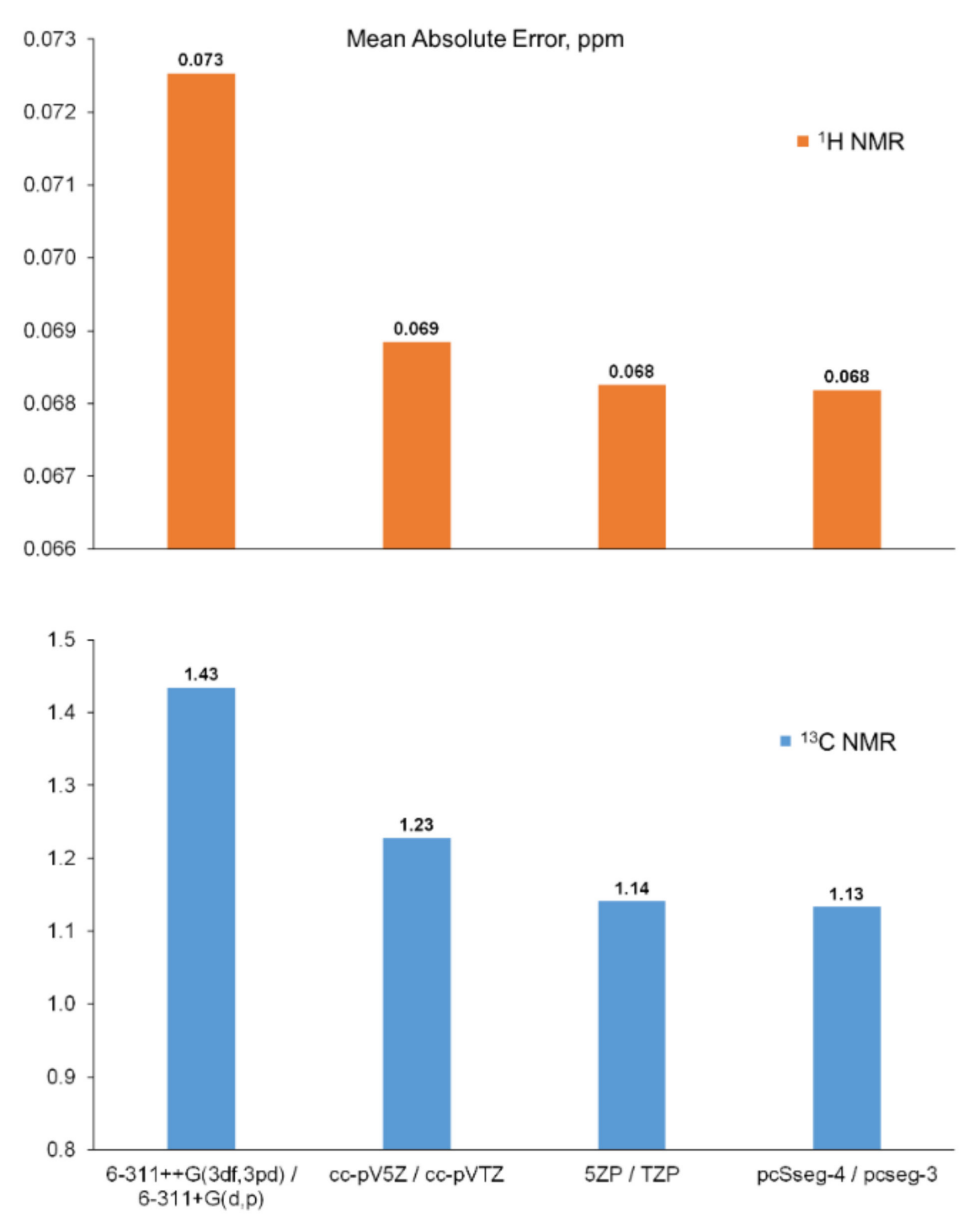
Mean absolute errors of ^1^H-NMR (**top**) and ^13^C-NMR (**bottom**) chemical shifts of strychnine (**2**) calculated with using the most extended locally dense basis set schemes, as compared to experiment. Reproduced from Semenov and Krivdin [67] with the permission of John Wiley and Sons.

As an example, various LDBS schemes were tested for the calculation of ^1^H and ^13^C-NMR chemical shifts of the benchmark strychnine (**2**) shown in Figure 4 [67] to find out the most efficient LDBS scheme based on the most popular basis sets of Pople, Dunning, Jorge, and Jensen. Those basis sets, which are currently most widely used for the calculation of chemical shifts, were examined as a function of the degree of their splitting, from double to quintuple quality. Shown in Figure 5 is the convergence of calculated ^1^H and ^13^C-NMR shielding constants of strychnine to the Complete Basis Set (CBS) limit exemplified for the family of Jensen’s basis sets. For all four types of examined basis sets, a regular approach of calculated ^1^H- and ^13^C-NMR shielding constants to their CBS limits was documented. The most effective results were demonstrated with Jensen’s basis sets, as illustrated in Figure 6. Indeed, it was found that the most accurate scheme was the combination of pcSseg4//pcseg-3, which was characterized by a MAE of only 0.07 ppm for the range of about 7 ppm for protons and that of only 1.13 ppm for ^13^C-NMR chemical shifts spread over the range of about 150 ppm for the molecule of strychnine.

As for spin-spin coupling constants, it is essential that using a cc-pVDZ basis set will surely yield results inferior to pcJ-n and pcJseg-n basis sets in spite of the fact that most people traditionally employ the former.

Different LDBS schemes were systematically employed for the calculation of NMR parameters at the DFT and non-empirical levels of theory in a number of recent publications by Rusakov et al. [68,69,70,71,72,73,74,75,76,77,78,79] and Semenov et al. [80,81,82,83,84,85], as well as in numerous publications by different authors, which are not cited herewith in full in view of their multiplicity.

#### 2.1.5. Solvent Effects

Nowadays, almost no one performs calculation of NMR parameters in gas phase, if compared to their experimental values measured in particular solvent. In practice, two basic approaches are usually applied to take into account solvent effects without inclusion solvent molecules in explicit way: (a)—the classical Tomasi’s Integral Equation Formalism Polarizable Continuum Model (IEF-PCM) [86,87,88,89] reviewed in [90] and (b)—the Conductor-like Polarizable Continuum Model (CPCM) [91,92,93,94,95].

The idea of the IEF-PCM approach is rather simple: solvent effects are simulated as an apparent charge distribution spread over the cavity surface without taking into account solute-solvent interactions at short distances, so that all solvent effects calculated within this scheme are constrained not to take into account any specific solvation effects. Very much the same, in the CPCM approach the solute molecule is embedded into a cavity surrounded by a dielectric continuum characterized by a dielectric constant *ε*. The accuracy of the latter depends on several factors with the most important one dealing with the proper boundary conditions on the surface of the cavity. CPCM defines the cavities as envelopes of spheres centered on atoms or atomic groups. Inside the cavity, the dielectric constant is the same as in vacuum; outside the cavity, it takes the value of a particular solvent.

**Figure 7:**
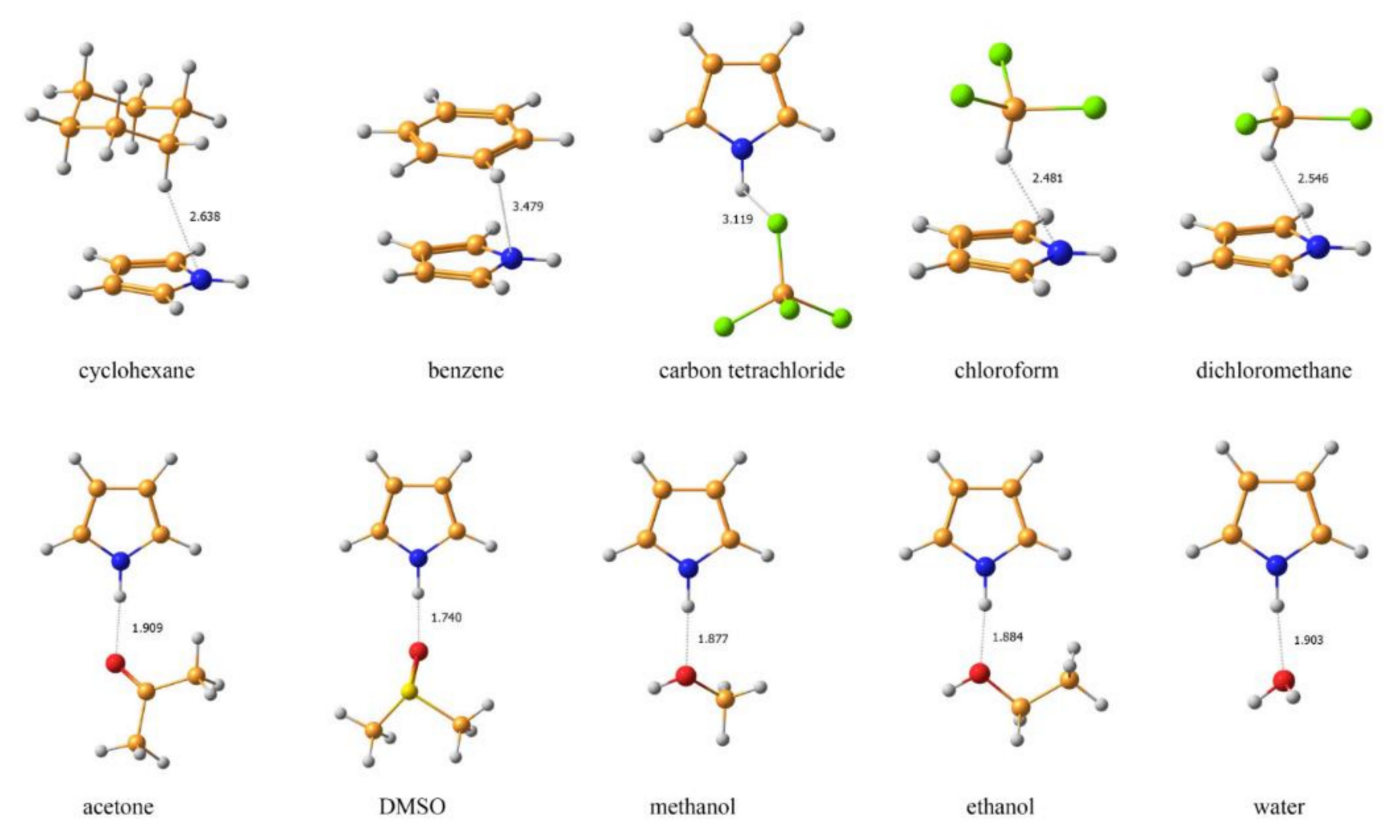
Equilibrium geometries of the SSM complexes of pyrrole with different solvents optimized at the MP2/6-311++G(d,p) level with taking into account bulk solvent effect within the IEF-PCM scheme. The most representative interatomic distances are given in Å. Reproduced from Semenov et al. [96] with the permission of John Wiley and Sons.

**Figure 8:**
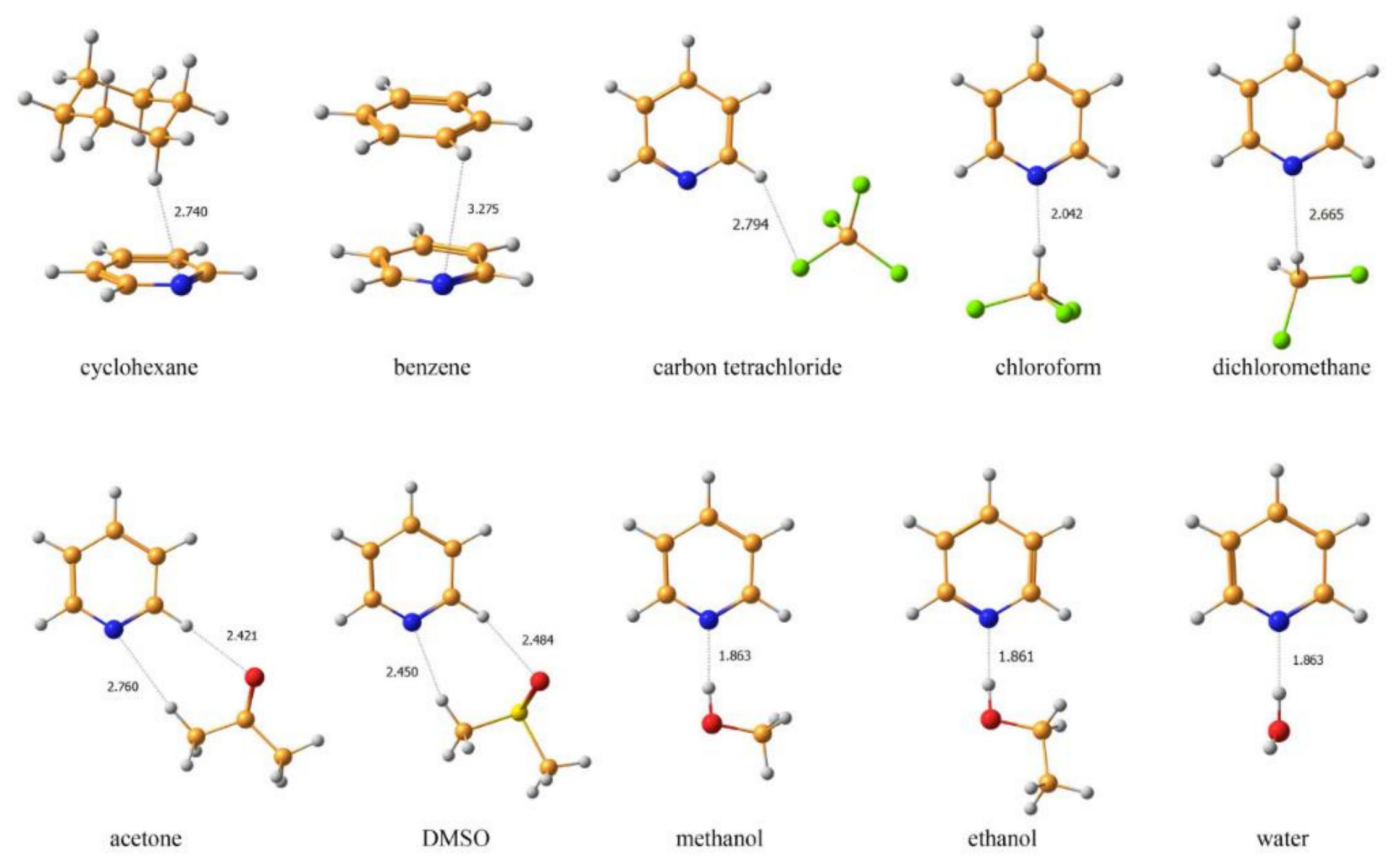
Equilibrium geometries of the SSM complexes of pyridine with different solvents optimized at the MP2/6-311++G(d,p) level with taking into account bulk solvent effect within the IEF-PCM scheme. The most representative interatomic distances are given in Å. Reproduced from Semenov et al. [96] with the permission of John Wiley and Sons.

**Figure 9:**
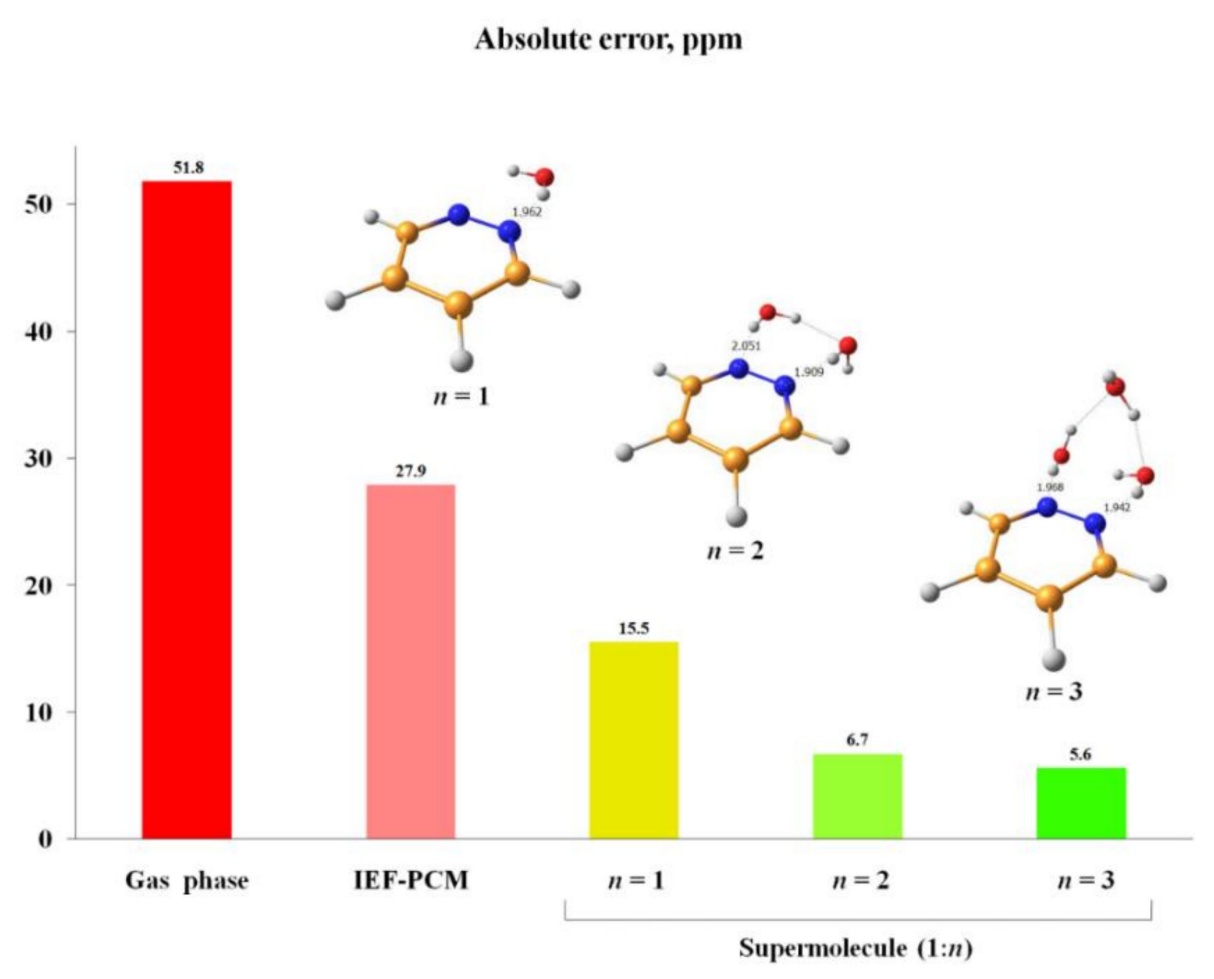
Absolute errors of the ^15^N-NMR chemical shift of pyridazine in water calculated at the GIAO-DFT-KT3/pcS-3//pc-2 level with using different solvation models to account for solvent effect. The most characteristic interatomic distances in the solvate complexes 1:1 (*n* = 1), 1:2 (*n* = 2) and 1:3 (*n* = 3) are given in Å. Reproduced from Semenov et al. [96] with the permission of John Wiley and Sons.

An alternative approach to take into account solvent effects is based on the inclusion of one, two or more molecules of solvent directly into calculation space and forming an explicit solvation shell, known as Supermolecular Solvation Model (SSM). As an example, in the paper by Semenov and coworkers [96] the ^15^N-NMR chemical shifts were calculated in a representative series of azoles and azines in a number of different solvents in gas phase and in the SSM media, as illustrated in Figure 7 and Figure 8. It was found that for polar solvents, the formation of the pyrrole complexes was due to a weak intermolecular bonding with participation of the pyrrole NH proton, while the pyridine complexes with polar protic solvents were formed due to the formation of the intermolecular hydrogen bonding involving pyridine nitrogen lone pair. It was also shown that in case of an obvious specific solvation of heterocycles containing two or more nitrogen atoms, *n* molecules of solvent were to be added into the calculation space where *n* should be not less than the number of nitrogen atoms in the heterocyclic moiety, as illustrated in Figure 9.

Four years later in parallel with those data, Caputo et al. [97] reported the results of their computational study of the NMR properties of glycine in water solution at the DFT level employing the B3LYP functional and Pople’s 6-31G(d,p) and Jensen’s pcSseg-2 basis sets. This level of theoretical treatment was used to describe solvent effects via either the PCM continuous solvation model or the PCM with additional explicit water molecules hydrogen-bonded to the solute. The authors observed that solvent caused considerable changes in the predicted magnetic shieldings and that the results depended significantly on the number of solvent molecules included in the calculation.

At the same time Lacerda et al. [98] presented a combined experimental and theoretical study of ^13^C- and ^1^H-NMR chemical shifts of the pyrrole derivatives. The authors investigated the importance of the solvation model, basis set, and quantum chemical method with the goal of developing a simple computational protocol, which allowed prediction of ^13^C- and ^1^H-NMR chemical shifts with sufficient accuracy for identifying such compounds in mixtures. It was found that common DFT level with the B3LYP functional was not sufficient for reproducing ^13^C-NMR chemical shifts, whereas already the simplest correlated MP2 level, lead to almost perfect agreement with the experimental data in liquid isotropic phase. It was demonstrated that taking into account solvent effects even at a simple PCM level generally improved the agreement of computational results with experiment. The only exception was the NH proton in the pyrrole moiety, which required inclusion of explicit solvent molecules in the calculation.

Finishing discussion of solvent effects, it should be noted in general that nowadays the quantum mechanics/molecular mechanics (QM/MM) calculations are also possible and are currently used for the computation of spin-spin coupling constants. Theoretical background and implementation of such calculations was presented in the early fundamental study [99]. A special attention was given to the role of explicit solvent polarization as well as the molecular consequences due to the hydrogen bonding. That model was shown to be generally applicable but implemented in that publication only at the DFT level for the liquid water and acetylene in aqueous solution. Good agreement between theory and experiment was obtained in both cases. Finally, spin-spin coupling constants across hydrogen bonds were discussed considering for the first time the role of an explicit solvent for spin-spin coupling constants.

#### 2.1.6. Vibrational Corrections

Manifestation and calculation of the vibrational (rovibrational) corrections to NMR parameters are very well documented and discussed in a number of earlier [100,101,102,103] and more recent [104,105] publications thoroughly discussed in two basic reviews [106,107]. Not surprisingly, taking into account molecular vibrational motion may plays a significant role in the calculation of NMR parameters. However, this fact is usually “omitted” by the authors since computation of vibrational corrections represents a very demanding task requiring evaluation of the parameters which are defined as the second and third derivatives of electronic potential energy (harmonic frequencies and anharmonic cubic force constants) together with corresponding gradients and Hessians with respect to the Cartesian displacement coordinates.

Usually vibrational effects in NMR properties are considered using the anharmonic vibrational wavefunction described within a second-order vibrational perturbation theory at a zero absolute temperature resulting in a Zero-Point Vibrational Correction (ZPVC) limit. Basically, ZPVC can be represented as consisting of two terms, harmonic and anharmonic, the latter reflecting the anharmonicity of the potential energy surface. Indeed, predicting ZPVC is an important step in the accurate evaluation of such molecular properties as NMR chemical shifts and spin-spin coupling constants. Theoretical details dealing with the vibrational corrections to NMR parameters are thoroughly discussed in a fundamental monograph on molecular electromagnetism by Sauer [8].

**Figure 10:**
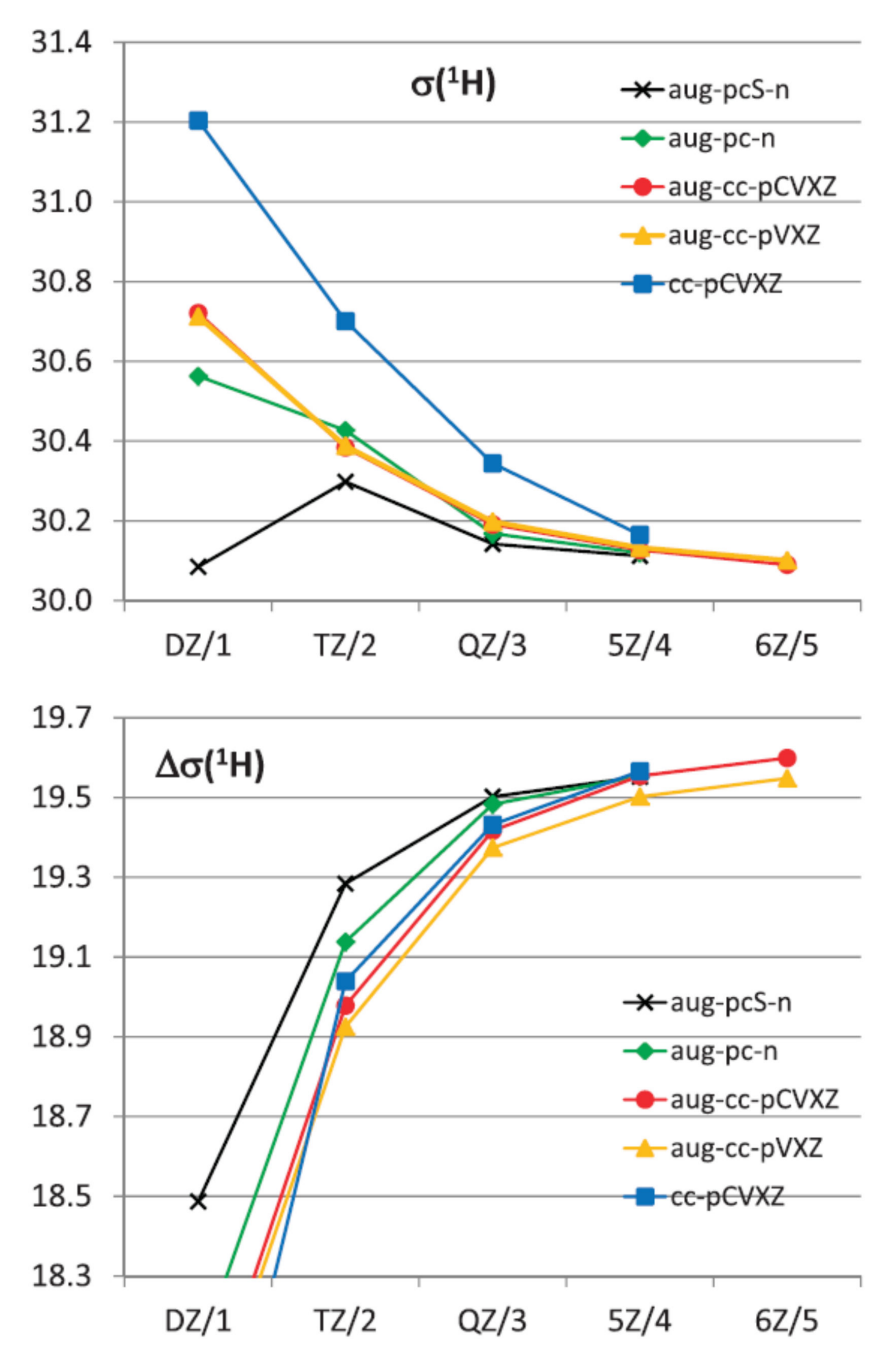
The ZPVC-averaged isotropic shielding (**top**) and shielding anisotropy (**bottom**) of hydrogen in the ^1^H-NMR spectrum (ppm) of water calculated at the CCSD(T) level as a function of the cardinal number X (or n) in the cc-pCVXZ, aug-cc-pVXZ, aug-cc-pCVXZ, aug-pc-n, and aug-pcS-n basis sets. Reproduced from Faber et al. [104] with the permission of Taylor & Francis.

The influence of the vibrational corrections to the values of isotropic shielding constants of hydrogen in ^1^H-NMR spectra of water calculated at the CCSD(T) level with different basis sets is exemplified in Figure 10 taken from a milestone publication in this field by Faber and coworkers [105]. This figure demonstrates the convergence of ZPVC towards the complete basis set limit for proton shielding constants and shielding anisotropies in the molecule of water. Shown herewith are the ZPVC-averaged isotropic shielding (top) and shielding anisotropy (bottom) of hydrogen in water calculated at the CCSD(T) level as a function of a cardinal number X (or n) in the cc-pCVXZ, aug-cc-pVXZ, aug-cc-pCVXZ, aug-pc-n and aug-pcS-n basis sets.

The basic conclusion of the performed study [104] was that for the total shieldings and anisotropies calculated with the largest basis sets either at equilibrium geometries or the vibrationally averaged values, the MP2 method overestimated the correlation effects while DFT underestimated them. The HF data behaved rather erratically, as compared to the CCSD(T) results leading to close agreement in the case of the ZPV averaged isotropic shielding of oxygen. With respect to the basis set dependence of both the equilibrium geometry and the vibrationally averaged values independent of the method employed, a rather slow convergence was established for the non-augmented basis sets, so that sufficient convergence was not achieved before the sextuple zeta level. Adding the diffuse functions significantly accelerated convergence apart for the anisotropy of the hydrogen shielding, and sufficient convergence was already achieved at the quintuple zeta level or for *n* = 3 in the case of the aug-pc(S)-n basis sets. While the aug-cc-p(C)VXZ results for the hydrogen shielding and all series of basis sets for the anisotropies exhibited a reasonable monotonic behavior which allowed for extrapolations, the oxygen shielding results approached the value of the largest basis in a way. The latter prevented a meaningful extrapolation of ^1^H and ^17^O-NMR shieldings to the CBS limit with the exception of the results of the two or three largest basis sets.

As for the ZPVCs to hydrogen and oxygen shieldings, it was observed that for the former, basically any method was good enough, while for the latter, there were significant differences for the levels of theory applied. While HF predicted a ZPV correction to the oxygen shielding close to the CCSD(T) result, the B3LYP and MP2 ZPVCs data were much worse. The main conclusion of that study was that one should not expect a priori that extrapolations to the CBS values of vibrational corrections were meaningful. Oscillatory convergence could occur, which inhibited extrapolations, since the commonly used forms of fit functions did not allow for such behavior.

In conclusion to the discussion of this section, it should be clearly understood that generally, vibrational effects do noticeably affect calculated values of NMR chemical shifts and spin-spin coupling constants. However, evaluation of vibrational corrections to NMR properties in the medium-sized organic molecules is enormously computationally demanding, so that the latter are usually not taken into account in most publications.

#### 2.1.7. Relativistic Effects

Relativistic effects play a major role in the calculation of NMR chemical shifts and spin-spin coupling constants of molecules containing heavy elements [23,108,109] which are usually not present in the molecules of carbohydrates and derivatives. However, for the sake of completeness, we will briefly discuss this item in the present review as well.

In general, relativistic effects in the values of NMR parameters include electron spin-orbit coupling (the interaction of the spin magnetic moment of an electron with the magnetic field induced by its own orbital motion) and scalar effects. The latter include the so-called Darwin term (relativistic fluctuation of an electron about its mean position) and mass-velocity corrections (relativistic increase in the mass of an electron with its velocity approaching the speed of light). These intermolecular effects dealing with the proximity of heavy nuclei to the magnetic isotopes strongly affect the values of NMR shielding constants of heavy nuclei and, on the other hand, light nuclei (like carbons and protons) in heavy environment. Accordingly, there are two most significant types of relativistic effects, namely the scalar effects, i.e., the ones which operators do not contain the electronic spin variable, and, on the other hand, spin-orbit relativistic effects, i.e., those which operators depend on electronic spin.

Scalar effects could be defined as the sum of the mass-velocity and Darwin corrections. In its turn, mass-velocity correction arises due to the relativistic increase of the electron mass, affecting the kinetic energy of an electron, while Darwin correction originates in the “smearing” of the moving electron, resulting in the change of its potential energy. On the other hand, spin-orbit effects originate in the interaction of an electron spin with its own angular momentum in relation to the position of the nucleus (known as the “one-electron effect”), or with its own angular momentum in relation to the position of another electron (known as the “two-electron effect”).

It should be noted that implementation of various LDBS schemes in view of the economy of computational resources is very effective in the enormously demanding two- and even more time consuming four-component relativistic calculations in compounds containing “heavy” elements beginning with the 4-th period. However, it should be emphasized that in a vast majority of cases relativistic effects may be safely ignored in the calculation of NMR parameters of carbohydrates.

## 3. Monosaccharides

Two recent comprehensive reviews from Serianni’s group dealing with the computational aspects [1] and stereochemical applications [2] of NMR chemical shifts and spin-spin coupling constants of saccharides together with a review by Toukach and Ananikov [7] have recently appeared. In the present review, we will not go into much detail, only covering in general the most illustrative examples in this field.

**Scheme 1:**
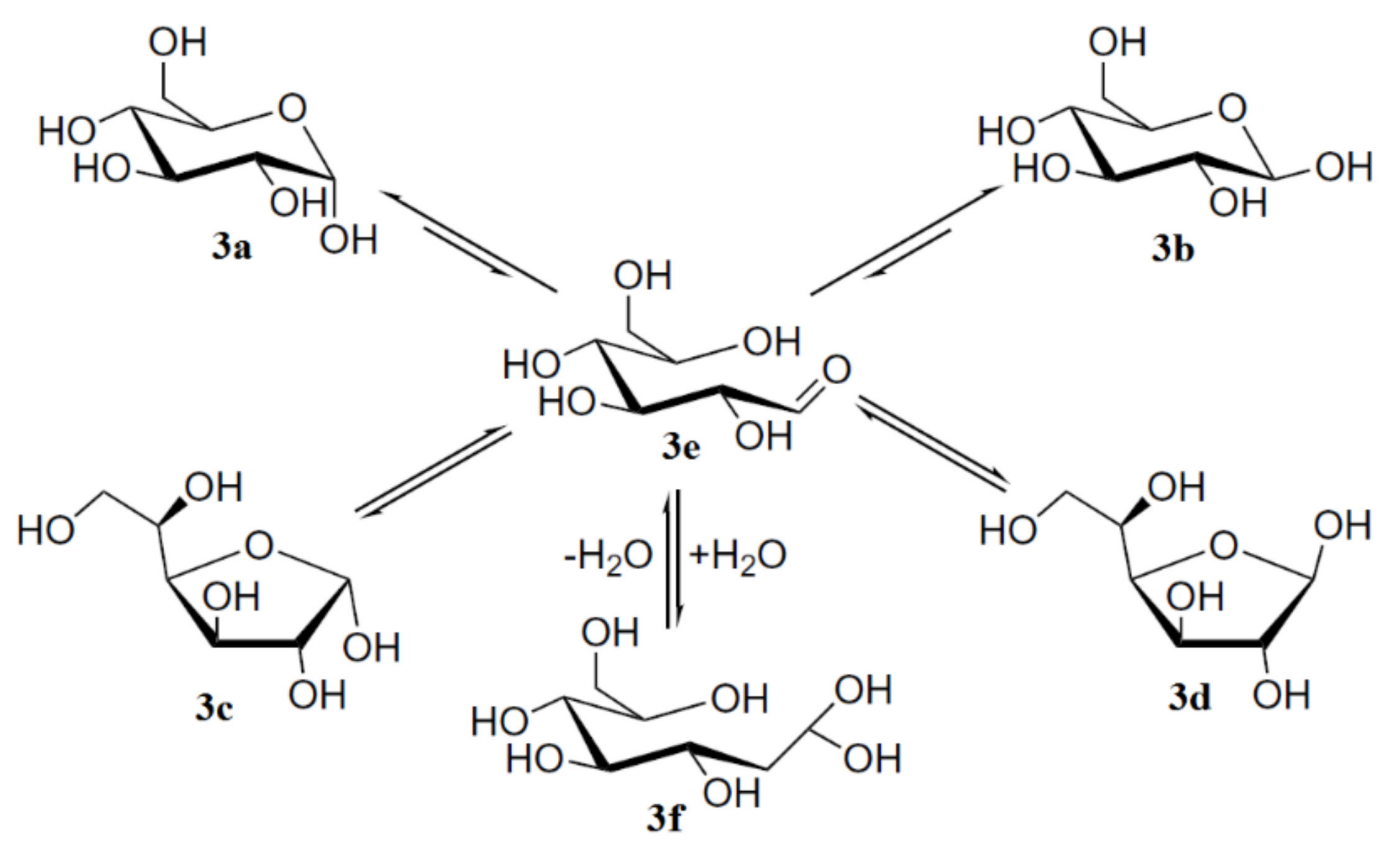
Mutarotation of D-glucose in solution. Reproduced from Roslund et al. [111] with the permission of Elsevier.

To begin with, it is well known that mutarotation of monosaccharides proceeds via the interconvertion of two hexopyranose and two pentofuranose forms through the open-chain intermediate which is exemplified below by the mutarotation of the most classical monosaccharide D-glucose, which was studied in much detail by Zhu and coauthors [110]. The results of that study demonstrated that concentrated solutions of aldohexoses in water represented almost all forms of D-glucose: α-pyranose, 37.63% (**3a**); β-pyranose, 61.96% (**3b**); α-furanose, 0.108% (**3c**); β-furanose, 0.28% (**3d**); together with the traces of the aldehyde form, 0.0040% (**3e**) and the hydrate form, 0.0059% (**3f**) shown in Scheme 1, the latter retrieved from Roslund et al. [111].

**Figure 11:**
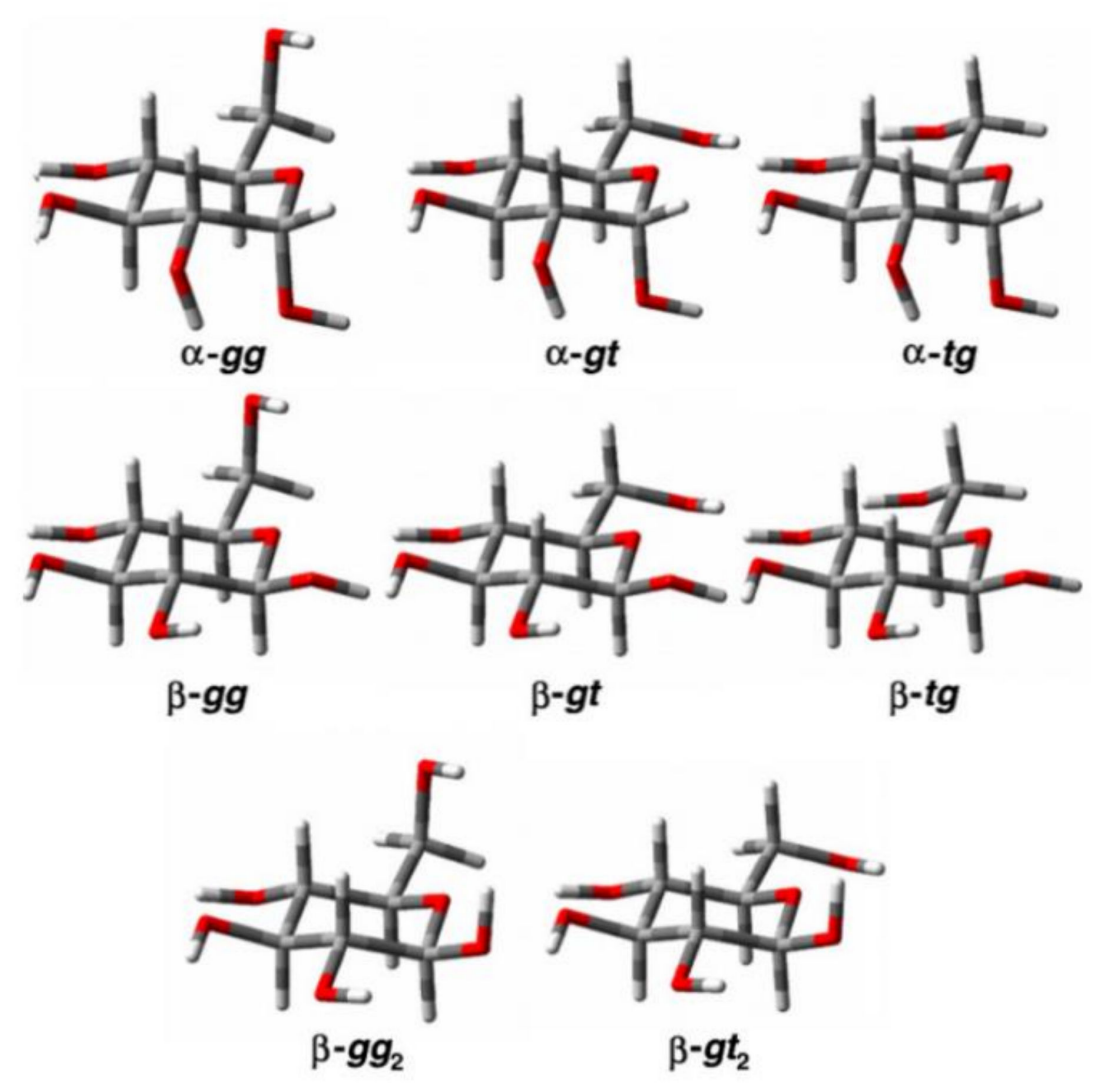
The conformers of α- and β-D-glucopyranose. Reproduced from Roslund et al. [111] with the permission of Elsevier.

In the cited study of the mutarotation of D-glucose in solution [111], the ^1^H and ^13^C nuclear shieldings and proton-proton coupling constants were calculated at the B3LYP/pcJ-2 level for the optimized structures of α- and β-D-glucose. Those structures were averaged in solution for a set of three conformers of α-D-glucose (α-gg, α-gt, and α-tg) and five conformers of β-D-glucose (β-gg, β-gt, β-tg, β-gg_2_, and β-gt_2_) shown in Figure 11, which were established earlier by Da Silva and coworkers [112].

**Figure 12:**
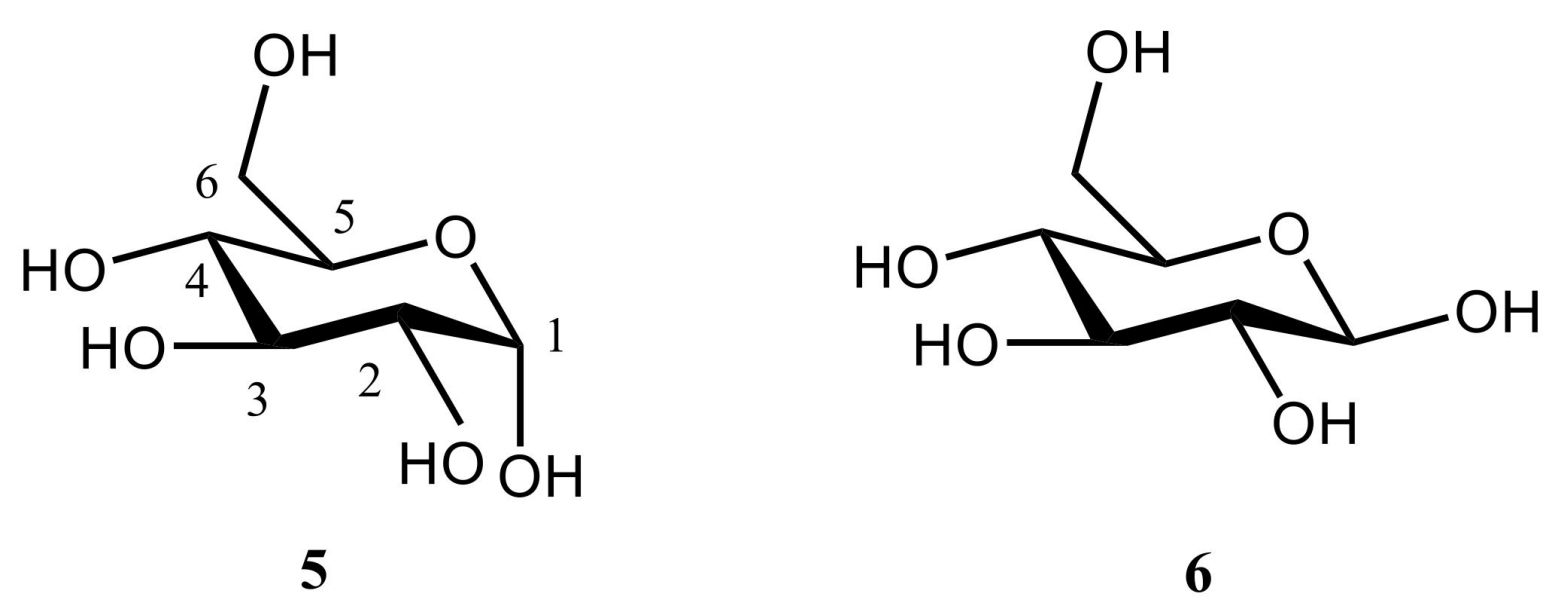
Anomeric forms of D-glucopyranose: α-anomer (**left**); β-anomer (**right**).

The correlation between the population weighted averages of the calculated ^1^H-NMR chemical shifts and corresponding experimental values given in Table 1 reached at in that study [111] was surprisingly good considering the fact that solvent effects were not taken into account in the geometry optimizations and NMR calculations. The largest Mean Absolute Deviations (MAD) between calculated and experimental values of ^1^H-NMR chemical shifts were less than 0.25 ppm (7%), and the overall mean absolute deviation was only 0.1 ppm (2.6%). Although the calculated ^13^C-NMR chemical shifts correlated well with the experimental values, the calculated chemical shifts of α and β anomers of D-glucopyranose shown in Figure 12 (accordingly, **5** and **6**) were about 10 ppm (some 13–14%) larger than corresponding experimental values.

**Scheme 2:**
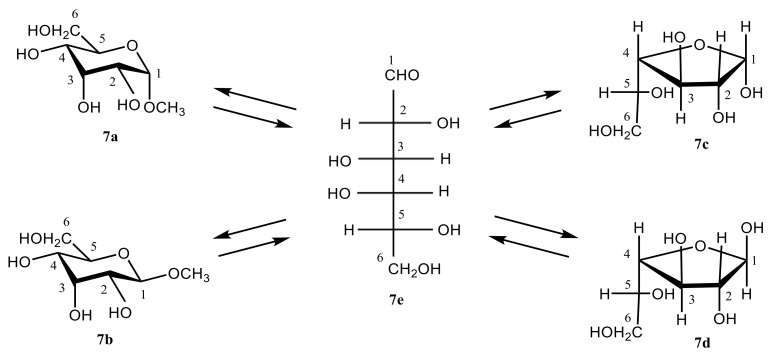
Mutarotation of D-galactose studied by Zrelov et al. [113]. Among monosaccharides, glucose plays an outstanding role due to its unique fundamental properties which are outlined below: (a)—aqueous solutions of glucose undergo mutarotation, leading to a mixture of α- and β-anomers with the heavily overlapping ^1^H and ^13^C-NMR spectra; (b)—its nonexchangeable protons lie in rather similar chemical environments and therefore give rise to a crowded ^1^H and ^13^C-NMR spectra which are difficult to interpret; and (c)—most importantly, hydroxyl groups of glucose anomers are located in such a way that an effective intramolecular hydrogen bonding is possible [114].

Much later, related studies were performed for some other monosaccharides, exemplified with D-galactose studied recently by Zrelov et al. [113]. The latter was shown to undergo interconversion of two hexopyranose forms of D-galactose, **7a** and **7b**, its two pentofuranose forms, **7c** and **7d**, and an open-chain intermediate **7e**, as illustrated in Scheme 2.

Keeping this in mind, Bagno and coworkers in their scrupulous study of α-D-glucose [114] performed computation of its ^1^H- and ^13^C-NMR chemical shifts in water through the combination of Molecular Dynamics (MD) and DFT calculations. Resulting structures were optimized with a B3LYP functional using Pople’s 6-31G(d,p) or 6-31+G(d,p) and Dunning’s cc-pVTZ or aug-cc-pVTZ basis sets.

**Figure 13:**
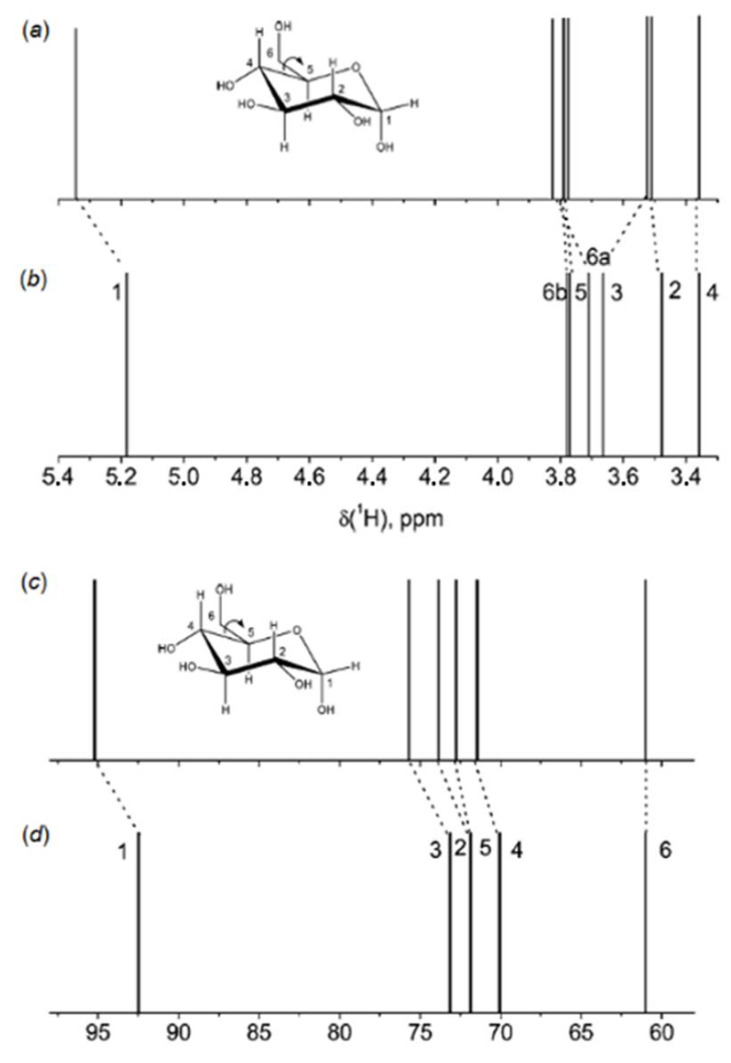
^1^H and ^13^C-NMR stick spectra of α-D-glucose in water: (**a**,**c**)—calculated; (**b**,**d**)—experimental. Calculated spectra were shifted to match the most shielded signals. Reproduced with minor editing privilege from Bagno et al. [114] with the permission of the American Chemical Society.

**Figure 14:**
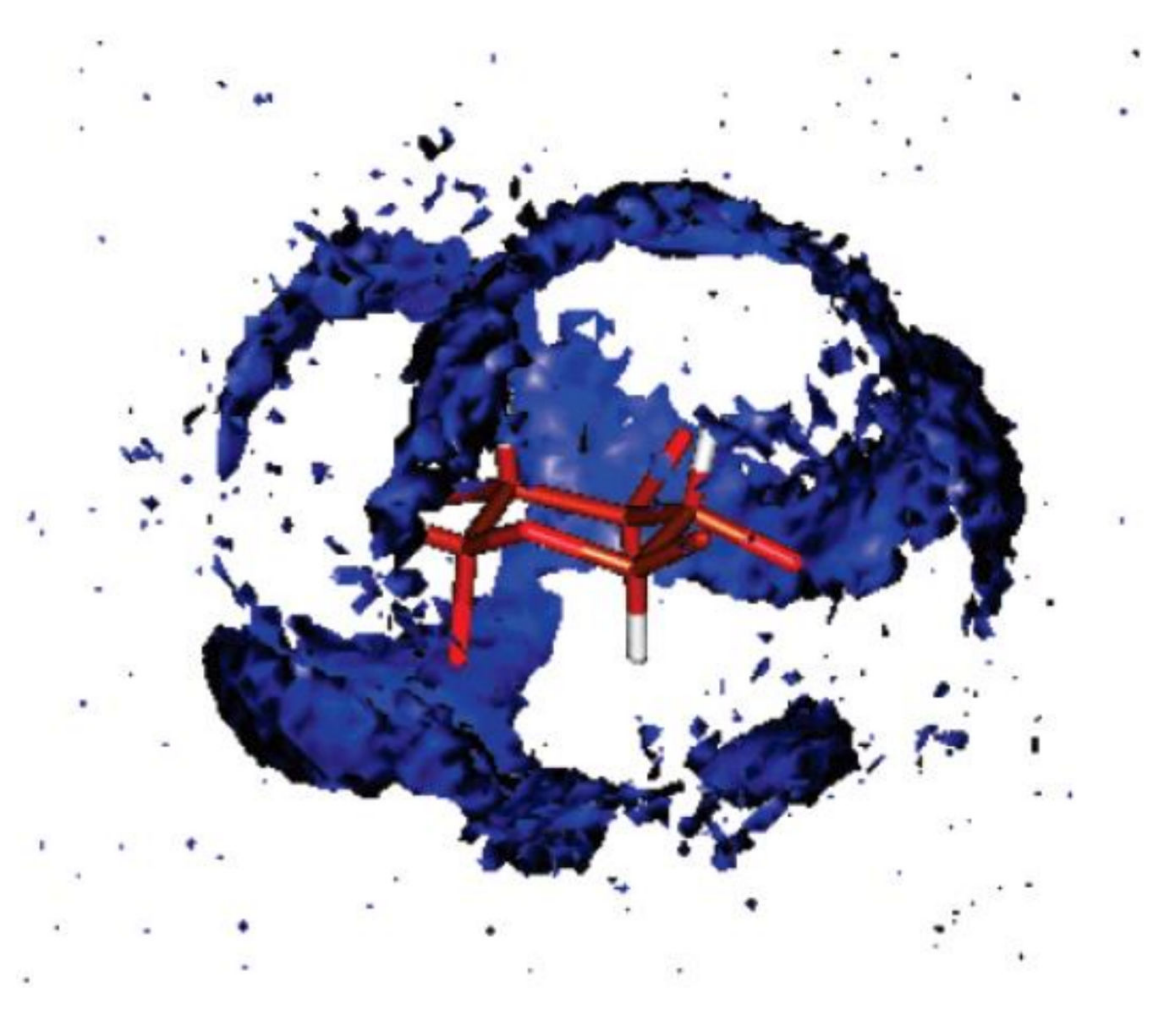
Spatial distribution function of the oxygen of water around glucose representing the probability of finding the oxygen atom of a water molecule at a given point in space around glucose. For clarity, the hydroxyl hydrogen and the hydroxymethylene hydrogen and oxygen atoms have been removed. This view shows the hydroxymethylene carbon and the ring oxygen at the forefront. Reproduced from Bagno et al. [114] with the permission of the American Chemical Society.

Further calculations of ^1^H- and ^13^C-NMR chemical shifts were carried out at the same level. The accuracy of the performed calculations was estimated by the authors as 0.1 ppm for protons and 1 ppm for carbons. A good agreement of the B3LYP calculations of ^1^H and ^13^C-NMR chemical shifts of α-D-glucose in water with taking into account solvent effects within the IEF-PCM formalism against experiment had been obtained, as illustrated in Figure 13. However, much better results following from the MD calculations with taking into account solvent effects of water in an explicit way as illustrated in Figure 14 were reached at in the same study [114].

**Figure 15:**
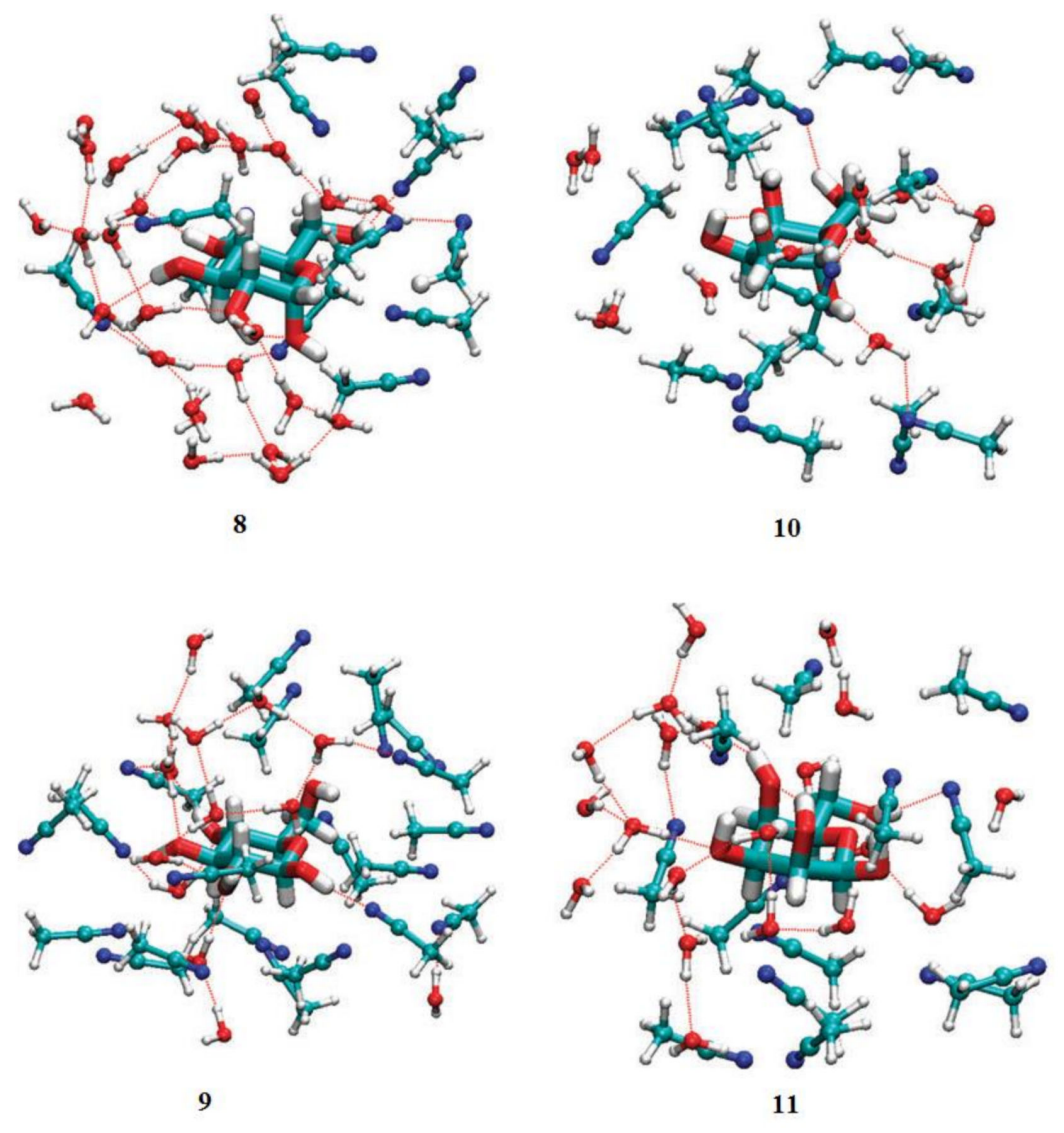
Snapshots of the solvation shell of four sugars obtained from the MD runs: α-glucose (**8**), β-glucose (**9**), α-talose (**10**), and β-talose (**11**). Reproduced with minor editing privilege from Bagno and Saielli [115] with the permission of the Royal Society of Chemistry.

Three years later the same principle authors [115] continued their studies dealing with the solvation of carbohydrates and performed relevant calculations on α- and β-glucose (**8** and **9**, respectively) and α- and β-talose (**10** and **11**, respectively) in mixtures of water and acetonitrile. The structure of the solvation shell, obtained by means of molecular dynamics simulation, has been analyzed using radial and spatial distribution functions. In agreement with available experimental data, water was found to preferentially solvate the sugars. The micro-heterogeneity of the mixture, with clusters of hydrogen-bonded water molecules and clusters of the dipole-dipole interacting acetonitrile molecules, favored the solvation of the carbohydrates by the water clusters, as shown in Figure 15 retrieved from that publication.

**Figure 16:**
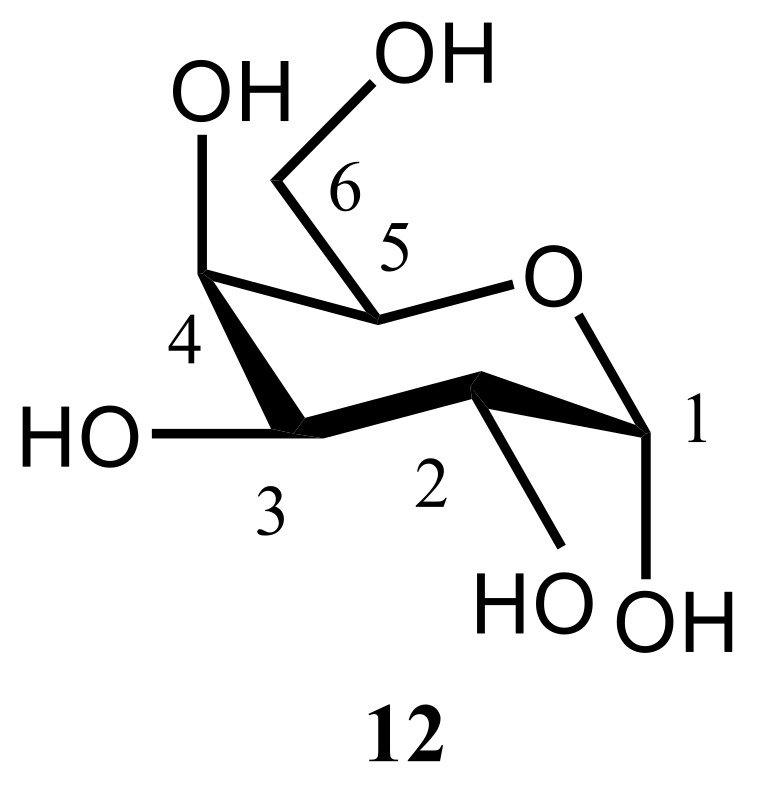
The structural formula of α-D-galactose.

**Figure 17:**
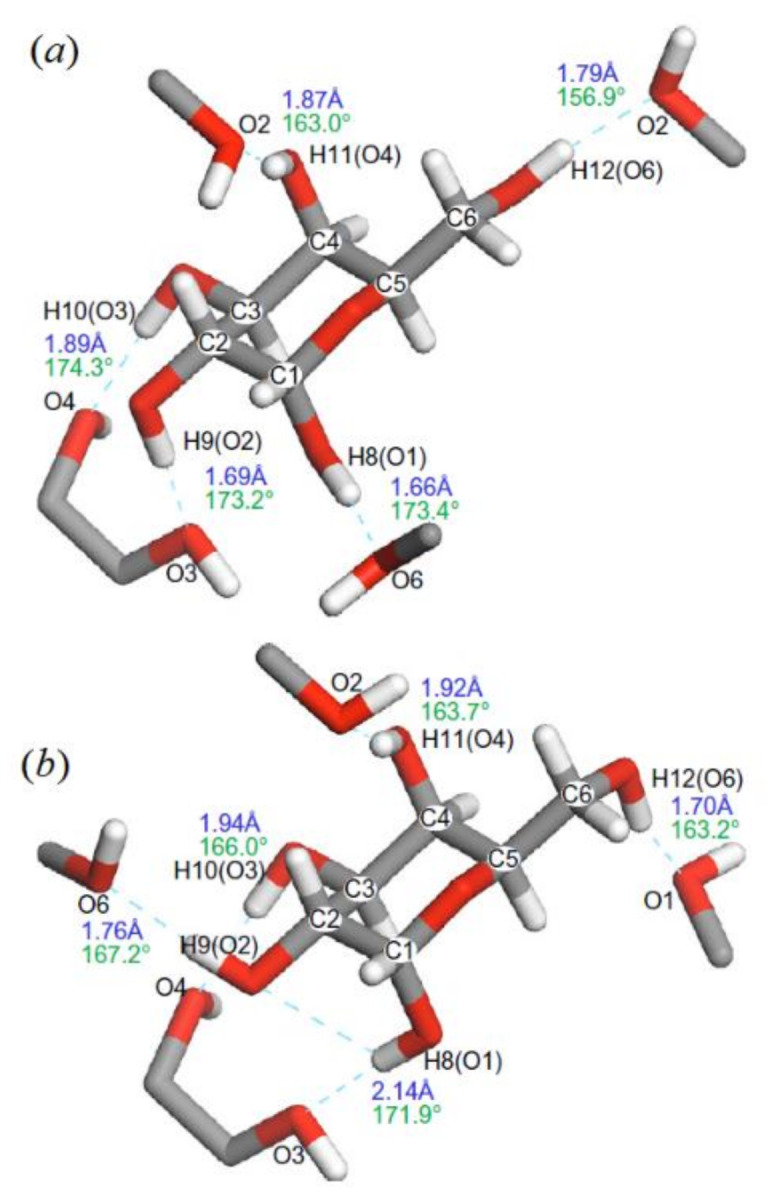
α-D-galactose with hydrogen atom positions optimized with the PBE exchange-correlation functional: (**a**)—Kouwijzer’s model (Model A) and (**b**)—Sheldrick’s model (Model B). The hydrogen bond networks are shown by dashed lines with calculated bond length (blue) and O–H∙∙∙O bond angles (green). Reproduced from Kibalchenko et al. [118] with the permission of Elsevier.

In the related study of the same period of time, Kibalchenko and coauthors [116] performed first principles calculations together with the solid-state NMR experiments to distinguish between possible hydrogen bonding networks in α-D-galactose **12** (Figure 16), so to say “in vivo”. Two theoretical models, namely those based on the X-ray structures of Kouwijzer (Model A) and Sheldrick (Model B) represented in Figure 17 were employed in those calculations.

In the latter (Model B), the network was a closed loop in which the longest hydrogen bond (2.13 Å) involved anomeric hydroxyl O^1^−H linked to the O^3^ atom, and the shortest (1.70 Å) linked the primary alcohol O^6^−H to the anomeric oxygen O^1^. In Model A, the O^1^‒H∙∙∙O^6^ hydrogen bond was still the longest (2.02 Å), but O^1^ was not a hydrogen bond acceptor, and the O^1^‒H∙∙∙O^6^ distance (1.69 Å) was the shortest in the hydrogen bonding network.

All ^13^C-NMR shielding parameters were calculated using the Gauge Including Projector Augmented Waves (GIPAW) approach, see original paper by Pickard and Mauri [117]. GIPAW is a DFT based method used to calculate magnetic resonance properties, exploiting the full translational symmetry of crystals. In this method, the use of pseudopotentials and plane waves provides an excellent balance of speed and accuracy. GIPAW presents a theory for the all-electron magnetic response within the pseudopotential approximation and is well known in the solid state NMR for its application to the calculation of the first-principles NMR chemical shifts. A Hamiltonian constructed using GIPAW has the required translational invariance in the presence of a magnetic field. In the original paper [117], the gauge-including projector augmented wave method was applied when describing the original projector augmented-wave method and extending it to the case of a uniform applied magnetic field. In fact, GIPAW is an extension to the projector augmented-wave method, which is valid for systems in the nonzero uniform magnetic fields in the gauge-including projector augmented-wave approximation. Implementation of GIPAW into a parallelized planewave pseudopotential code allows the calculation of NMR chemical shifts in large, low-symmetry extended systems.

As an example, Szeleszczuk and coauthors [118] demonstrated a convenient method for the indirect crystal structure verification of methyl glycosides using the GIPAW method. In that paper, instead of using various time consuming methods of conformational search, the crystal structures of methyl glycoside acetates were used for the desired crystal structure preparation and optimization. Due to the high sensitivity of chemical shifts to the local atom neighborhood, the method of structure verification based on the GIPAW calculations was found to be very promising. Since performed calculations included both the symmetry and periodicity of the crystal structures, the results depended upon the correct representation of intermolecular interactions, especially hydrogen bonding which is known to be dominant in the crystal structures of carbohydrates with freely rotating hydroxyl groups. It was found that the NMR-based method of crystal structure verification could provide information about the fragments of carbohydrate that need to be reoriented in order to obtain the correct crystal structure. Finally, in spite of not taking into account temperature effects for the correct structures, the correlations between experimental and theoretical chemical shifts were found to be very reliable. It was thus concluded that temperature effects were not essential in the case of methyl glycosides, which may not be so in the case of other classes of saccharides.

Coming back to the paper by Kibalchenko and coauthors [118], a partial geometry optimization in that study was carried out prior to calculating the NMR parameters. The isotropic chemical shifts (represented here with ^13^C-NMR chemical shifts in Table 2) were obtained from the isotropic average of the shielding tensors evaluated with the PBE and KT3 functionals. Based on those results, it was concluded that the Kouwijzer structure for α-D-galactose (Model A) was much closer to experiment, as compared to Model B.

Also, as was commented by one of the referees, for the calculation of the NMR parameters of carbohydrates in solid state, some more specific functionals (other than those used for the liquid state calculations) have been proven to be significantly more accurate, such as PBE with dispersion correction (PBE + TS).

For the nuclei with a spin of more than 1/2 exhibiting a quadrupole moment, the Electric Field Gradient (EFG) occurs, which results in an essential peak broadening in the solid-state NMR spectra. In this case, the EFG calculations should be performed in order to determine the electric field gradients for each atom in the system, especially in terms of the quadrupole interactions. Indeed, “a quadrupolar nucleus is efficiently relaxed by a non-uniform electric field that is a product of the solute molecules interaction with the dipolar solvent. This relaxation is dependent on the interaction of the electric field gradient at the nucleus. When the nucleus is in a molecule that is surrounded by a non-spherical electron density distribution, it creates a gradient. The field gradient, *q*, describes the electron charge cloud’s deviation from spherical symmetry. The value of *q* is found to equal zero if the groups around the quadrupolar nucleus have a cubic symmetry, such as in the T_d_ point group. However, if a non-cubic molecule has a threefold or higher symmetry axis, the deviation from spherical symmetry is expressed as a magnitude of *q*. The two parameters, *q*, the field gradient, and η, the asymmetric parameter, become necessary only if the molecule’s point group’s highest symmetry axis is a threefold symmetry or less. Depending on the molecule, certain cancellations can take place leading the asymmetric parameter, η, to equal zero. This is caused by a combination of very specific bond angles and charge distribution in the molecule being analyzed. Ultimately, the effectiveness of the relaxation is dependent on the magnitude of the electric field gradient, *q*. Linewidth broadening in the NMR spectrum is consequential of the rapid nuclear quadrupole relaxation of the quadrupole nucleus. Consider an analogous situation: chemical exchange. It is known that when the nuclei’s spin state rapidly changes it causes broadening in the spectrum. Similarly, the nuclear quadrupole relaxation rates of a quadrupolar nucelus corresponds to an intermediate rate of chemical relaxation.The apparent broadening effect also influences the spectra of the other nuclei attached to the quadrupolar nucleus, including protons. In some cases, the rapid nucleur quadrupole relaxation times (T_1_) can cause extensive homogenous broadening (consequential of readily relaxing nuclei) rendering the proton signal of the quadrupolar nucleus completely unobservable in the ^1^H-NMR spectrum. T_1_ is determined by two factors: the electric quadrupole moment (*Q*) and the presence of the electric field gradient (*q*) across the nucleus. A common approach to resolving quadrupolar effects on the spectra of solution state NMR is elevating temperatures while collecting NMR data. The molecular reorientational correlation times are then shorter than the normal time scale, so the homogenous broadening of the line can be reduced. Unfortunately, the temperature required to create this motional tapering is unfeasibly high for many samples that would deem this technique necessary” [119].

Coming to the calculation of ^1^H-^1^H spin-spin coupling constants of monosaccharides, it should be recalled that their stereochemical applications were historically limited to the implementation of four vicinal proton-proton couplings, namely, ^3^*J*(H-1,H-2), ^3^*J*(H-2,H-3), ^3^*J*(H-3,H-4), and ^3^*J*(H-4,H-5), which are most critical to the conformation of the hexapyranosyl moiety. One of the most illustrative examples of stereochemical dependence of these coupling constants is the assignment of a highly preferred ^4^C_1_ conformation of methyl β-D-glucopyranoside in aqueous solution (see below). This assignment was based on the magnitudes of the most informative vicinal proton-proton coupling constants of the pyranose ring, calculated theoretically and compared to experiment.

**Figure 18:**
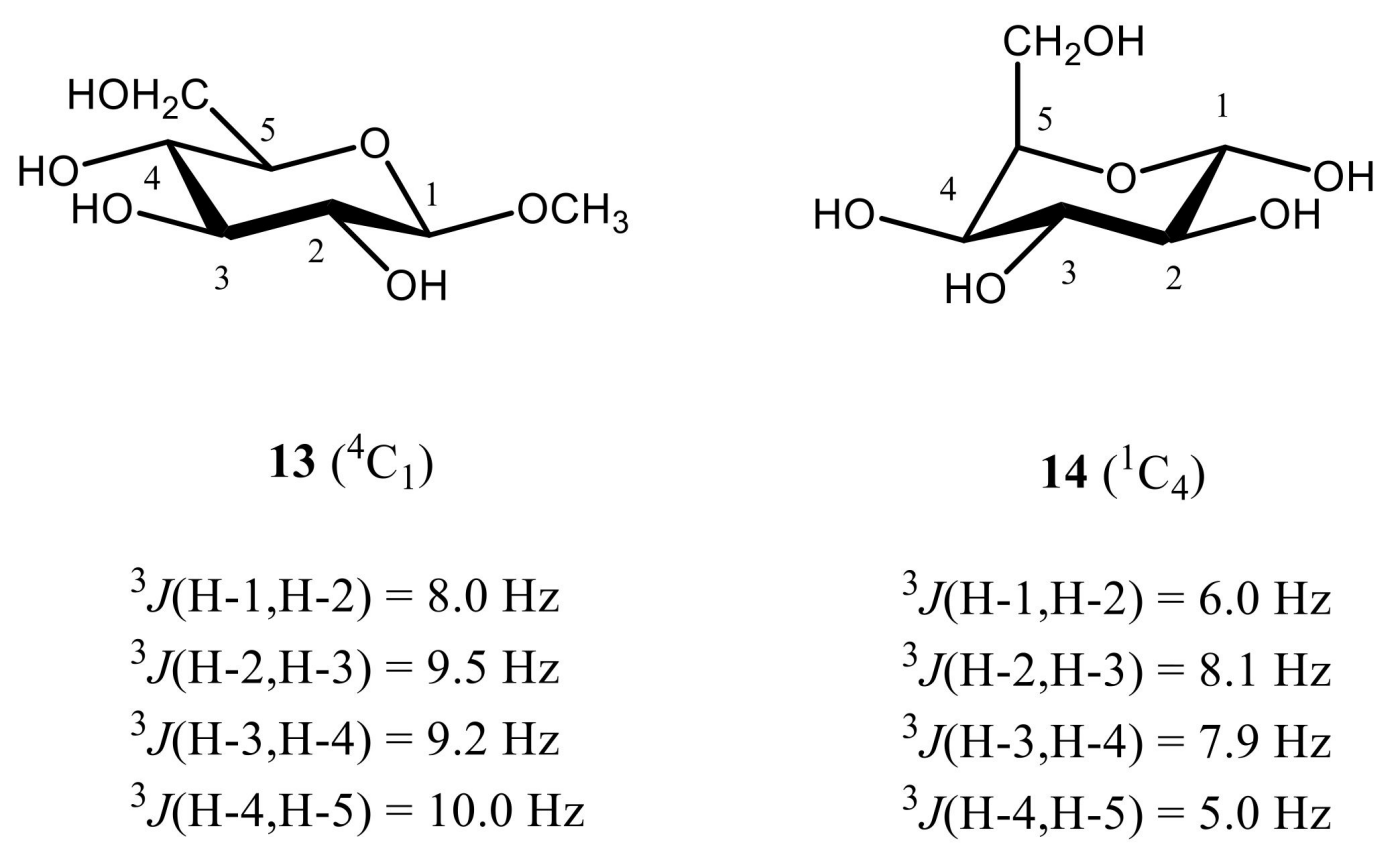
Most informative vicinal proton-proton spin-spin coupling constants in the representative β-D-glucopyranoside (**13**) and α-D-idohexopyranose (**14**), providing accordingly, ^4^C_1_ and ^1^C_4_ conformations. Adopted from the review [1].

As follows from numerous calculations and experimental measurements, all of those couplings are essentially large (8 Hz or larger), which indicates on the fact that all coupled protons are in the axial positions of the pyranose ring characterized by their mutual transoidal orientations. Such a behavior markedly contrasts to that of α-D-idohexopyranose, where corresponding ^3^*J*_H,H_ couplings range from about 5 to 8 Hz, which suggests a highly preferable alternative ^1^C_4_ conformation, as compared to a classical ^4^C_1_ one. The latter is characterized by a highly energetically unfavorable axial orientation of all four hydroxyl groups at C-1, C-2, C-3, and C-4 positions of the pyranose ring and equatorial orientation of the CH_2_OH group at C-5. On the contrary, in the alternative ^1^C_4_ conformation of all four hydroxyl groups occupy favorable equatorial orientations while only CH_2_OH group is in the unfavorable axial orientation, as illustrated below for methyl β-D-glucopyranoside (**13**) and α-D-idohexopyranose (**14**), see Figure 18.

It thus follows that in monosaccharides, four ^3^*J*_H,H_ couplings are especially sensitive to the pyranosyl ring conformation, namely ^3^*J*(H-1,H-2), ^3^*J*(H-2,H-3), ^3^*J*(H-3,H-4), and ^3^*J*(H-4,H-5), which is mostly representative for the considered β-D-glucopyranoside providing mostly ^4^C_1_ conformation and α-D-idohexopyranose existing mainly in the alternative ^1^C_4_ conformation.

In some cases, geminal, vicinal, and long-range (over more than three bonds) ^1^H-^1^H spin-spin couplings can be utilized for this purpose based on their structural and conformational dependencies, which can be used, as an example, in the conformational analysis of *O*-glycosidic linkages in polysaccharides. In total, as many as 68 one-bond, geminal, and vicinal coupling constants (namely, 14 ^n^*J*_CC_, 41 ^n^*J*_CH_, and 13 ^n^*J*_HH_,) were reported for β-D-glucopyranoside, which redundantly describe configuration and conformation of the glucopyranosyl moiety. However, only four most informative proton-proton couplings are usually employed in the stereochemical analysis of carbohydrates, while the rest of those coupling constants are used only sporadically.

It should also be noted that ^2^*J*_H,H_ couplings are affected by the conformation of the C–O bond adjacent to the exocyclic hydroxymethyl carbon while ^3^*J*_H,H_ are strongly influenced by the relative arrangement of the hydroxyl groups attached to carbons bearing coupled hydrogens [120,121,122]. In some pyranosyl ring conformations, longer-range four-bond ^1^H-^1^H coupling constants depend on the geometry of the corresponding four-bond H-C-C-C-H coupling path [123] as well as on the relative arrangement of hydroxyl groups along the coupling pathway, which was well reproduced in the performed DFT calculations. These trends provided an additional guiding thread to the conformational behavior of the pyranose ring.

**Scheme 3:**
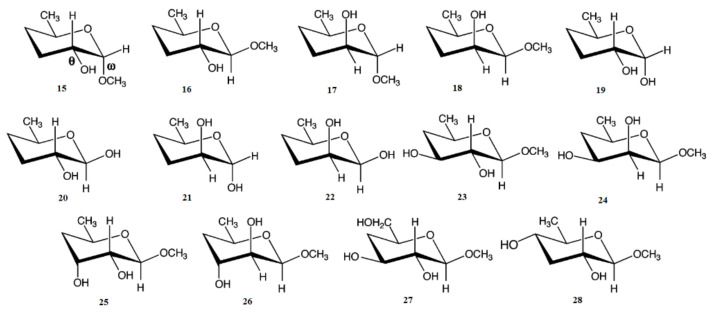
Model compounds chosen to represent α- and β-D-aldohexopyranoses and methyl α- and β-D-aldohexopyranosides. Reproduced from Zhao et al. [124] with the permission of the American Chemical Society.

**Figure 19:**
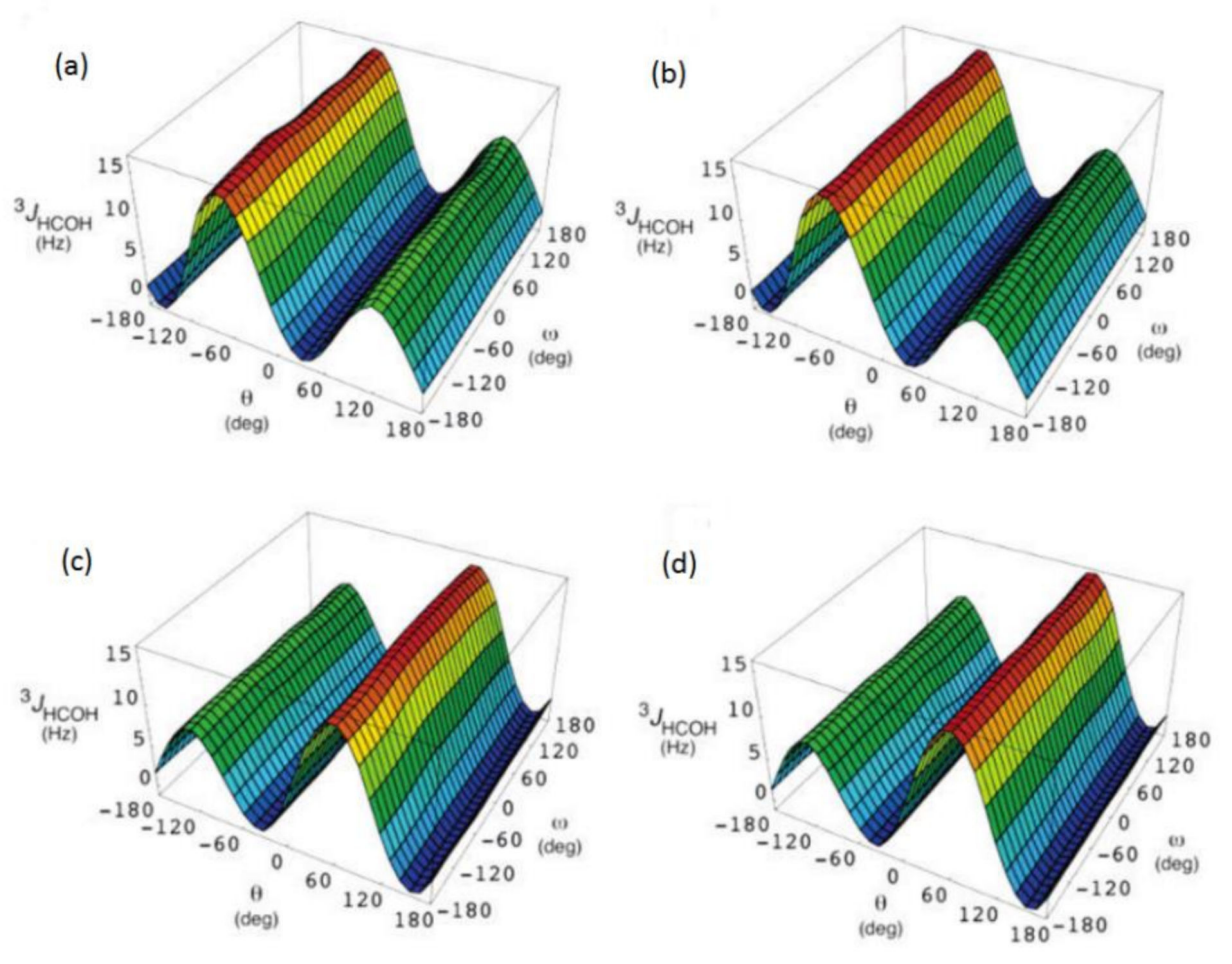
Dependences of calculated ^3^*J*(H-2,O-2H) on the dihedral angles ω and θ in (**a**)—compound **15**, (**b**)—compound **16**, (**c**)—compound **17**, and (**d**)—compound **18**. Reproduced from Zhao et al. [124] with the permission of the American Chemical Society.

**Figure 20:**
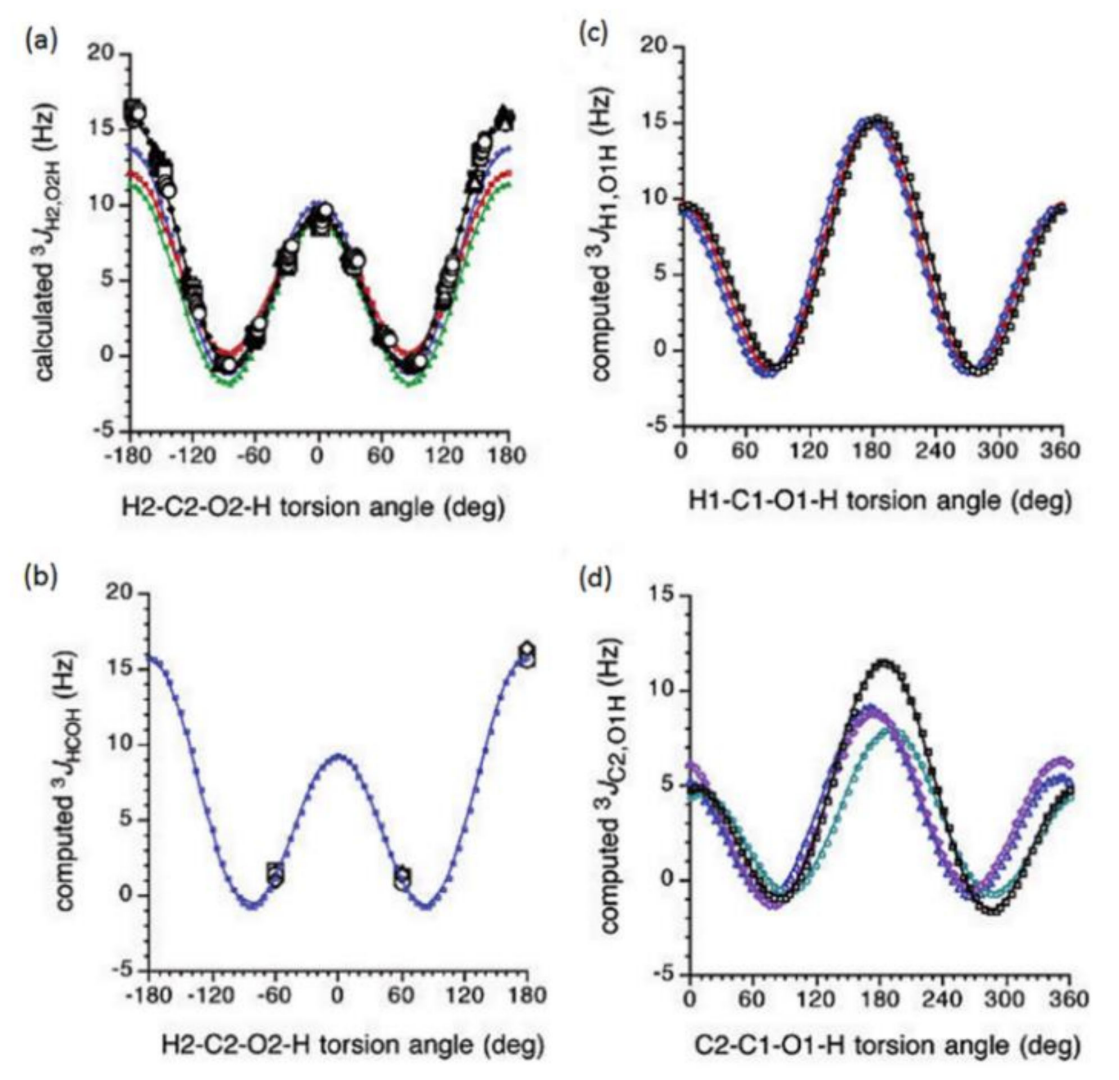
Dihedral angle dependences of vicinal ^1^H-^1^H spin-spin coupling constants in model compounds chosen to mimic methyl α- and β-D-aldohexopyranosides: (**a**)—Dependence of ^3^*J*_H2,O2H_ on the H^2^-C^2^-O^2^-H torsion angle θ in **15**–**18**; (**b**)—Dependence of ^3^*J*_H2,O2H_ on the H^2^-C^2^-O^2^-H torsion angle in **15**–**18**; (**c**)—Dependence of ^3^*J*_H1,O1H_ on the C^1^-O^1^ bond torsion in **19**–**21** (blue triangles) and **20**–**22** (black squares); (**d**)—Dependence of ^3^*J*_C2,O1H_ on the C^1^-O^1^ torsion angle in **19** (green circles), **20** (blue triangles), **21** (black squares), and **22** (purple diamonds). Reproduced with minor editing privilege from Zhao et al. [124] with the permission of the American Chemical Society.

In the earlier classical study in this area, Zhao and coworkers [124] performed a comprehensive theoretical study of the dihedral angle dependences of vicinal ^1^H-^1^H spin-spin coupling constants based on their pronounced Karplus dependences in the series of model compounds **15–28** shown in Scheme 3, representing methyl α- and β-D-aldohexopyranosides and corresponding α- and β-D-aldohexopyranoses. The detailed analysis of calculated one- and two-dimensional dihedral angle dependences of vicinal ^1^H-^1^H spin-spin coupling constants in these compounds performed in that study at the DFT level are presented in Figure 19; Figure 20. Those results demonstrated that ^3^*J*_HCOH_ depended primarily on the H-C-O-H dihedral angle and that it was essentially unaffected either by the conformation at the adjacent C−O bond or by the type of the carbon atom bearing hydroxyl group.

In general, those findings provided an essential support for the use of a generalized Karplus equation to interpret ^3^*J*_HCOH_ coupling constants in the broad scope of saccharides. However, it was found that separate equations were needed to treat ^3^*J*_H1,O1H_ because those couplings were subjected to the additional effect of the internal electronegative substituents. The latter effect caused phase shifting of the corresponding Karplus curves, which was due to the nonequivalent values of the gauche couplings. It was demonstrated that ^3^*J*_H,H_-based analysis of H^1^−C^1^−O^1^−H dihedral angles was very likely to reflect glycoside conformations. Furthermore, a high presence of the OH groups in those structures resulted in the unique chemical and physical properties of those compounds due to their ability to form intra- and intermolecular hydrogen bonds, which should lead in the final diagnosis to a more straightforward analysis of their C−O rotamer populations [124].

**Figure 21:**
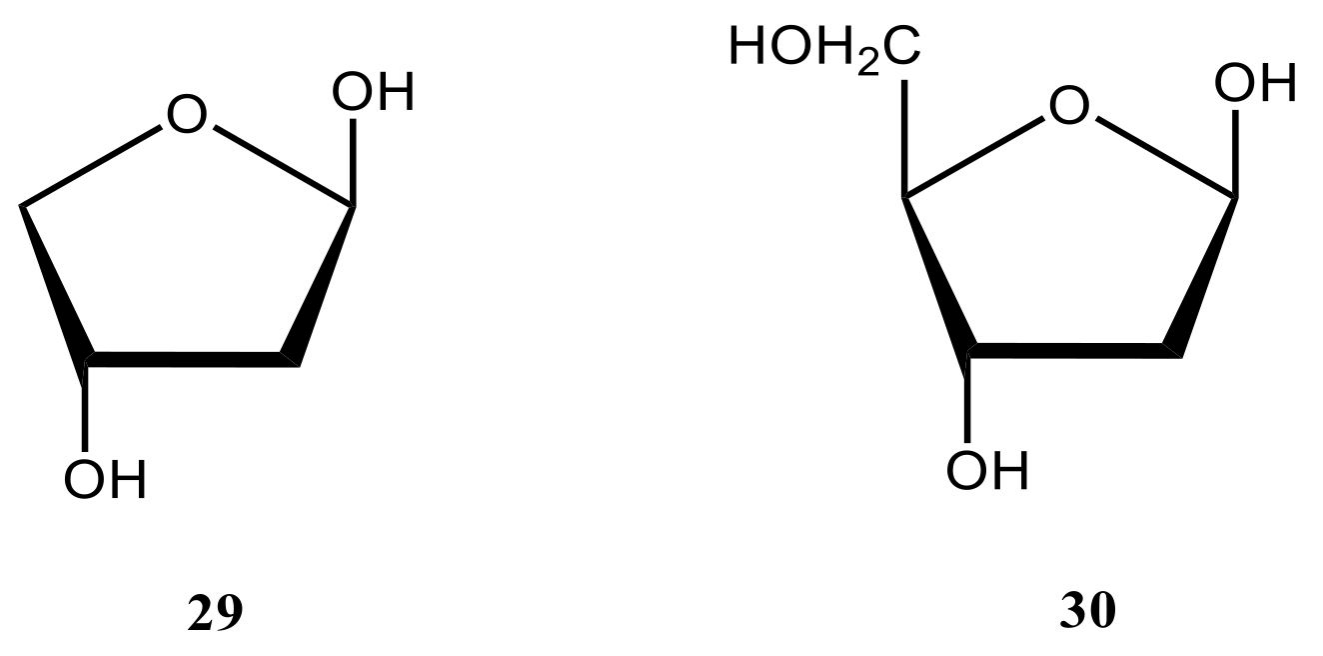
The structural formulas of 2-deoxy-β-D-erythro-pentofuranose (**30**) and its model form (**29**).

Coming to the substituent and stereochemical effects on carbon-hydrogen coupling constants in carbohydrates, it should first be recalled that the one-bond couplings drastically depend on their stereochemical structure and, on the other hand, on carbon *s*-character or the length of the corresponding carbon-hydrogen bond [125]. Thus in the cited paper, one-bond ^13^C-^l^H coupling constants have been studied in the furanose cycle of 2-deoxy-β-D-glycerotetrofuranose (**29**), a restricted model of 2-deoxy-β-D-erythro-pentofuranose (**30**) met in DNA, as a function of ring geometry in order to assess their utility as conformational probes. The ab initio calculations were conducted in that study on ten envelope forms and the planar form of **29** (modelling that of **30**) (Figure 21) at the MP2 level with Pople’s 6-31G(d) basis set. Computed ^1^*J*_CH_ values were found to be sensitive to the orientation of the C‒H bond, with larger couplings observed when a C‒H bond occupied the quasi-equatorial position.

**Scheme 4:**
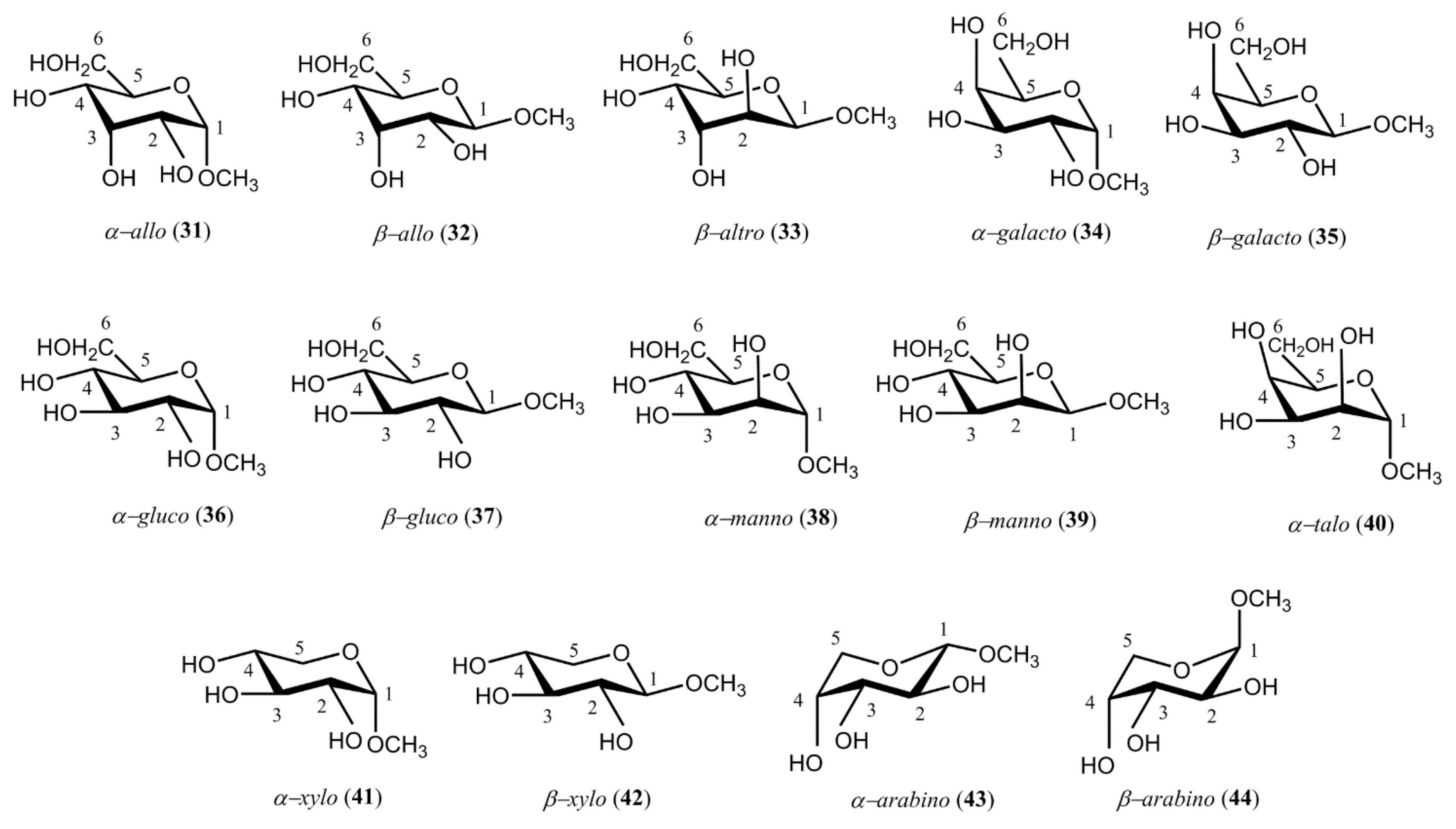
Representative structures of methyl D-aldopyranosides studied by Podlasek and coworkers [126].

At the same period of time, Podlasek et al. [126] performed a fundamental survey of carbon-hydrogen coupling constants in the representative series of methyl D-aldopyranosides **31**–**44**. Those compounds favored ^4^C_1_ conformations for methyl α-D-allopyranoside (**31**), methyl β-D-allopyranoside (**32**), methyl β-D-altropyranoside (**33**), methyl α-D-galactopyranoside (**34**), methyl β-D-galactopyranoside (**35**), methyl α-D-glucopyranoside (**36**), methyl β-D-glucopyranoside (**37**), methyl α-D-mannopyranoside (**38**), methyl β-D-mannopyranoside (**39**), methyl α-D-talopyranoside (**40**), methyl α-D-xylopyranoside (**41**), and methyl β-D-xylopyranoside (**42**). On the other hand, methyl α-D-arabinopyranoside (**43**) and methyl β-D-arabinopyranoside (**44**) preferred the ^1^C_4_ conformation, see Scheme 4.

The general findings of the performed study [126], exemplified here for the one-bond carbon-hydrogen coupling constants involving C-1 and C-2 in pyranoses, are outlined below.

One-bond carbon-hydrogen coupling constants involving C-1:

One-bond coupling constants ^1^*J*(C-1,H-1) of equatorial C−H bonds are about 10 Hz larger than those of axial bonds. Based on that tendency,

One-bond carbon-hydrogen coupling constants involving C-2:

The difference between ^1^*J*(C-2,H-2) of equatorial and axial C−H bonds (ca. 3.5 Hz) is much less pronounced than in the case of ^1^*J*(C-1,H-1) (ca. 10 Hz), so that those coupling constants are insufficient for a reliable use in assigning configuration at C-2 of aldopyranosyl rings. Geminal couplings ^2^*J*(C-2,H-3) fall within two groups, one showing large absolute values of 4.9 ± 0.6 Hz (α-allo, β-allo, β-arabino, α-galacto, β-galacto, and β-gluco pyranosides) which are negative in sign, and the other yielding smaller values of 1.2 ± 0.4 Hz (α-manno-, β-manno-, and α-talopyranosides) being probably positive in sign as well. Vicinal couplings ^3^*J*(C-2,H-1) and ^3^*J*(C-2,H-3) provide no pronounced Karplus type dependence on dihedral angles which is probably due to the oxygen lone pair effect.

Experimental trends highlighted in that publication [126] were substantiated by numerous calculations from this group, which were reviewed later [1,2].

**Figure 22:**
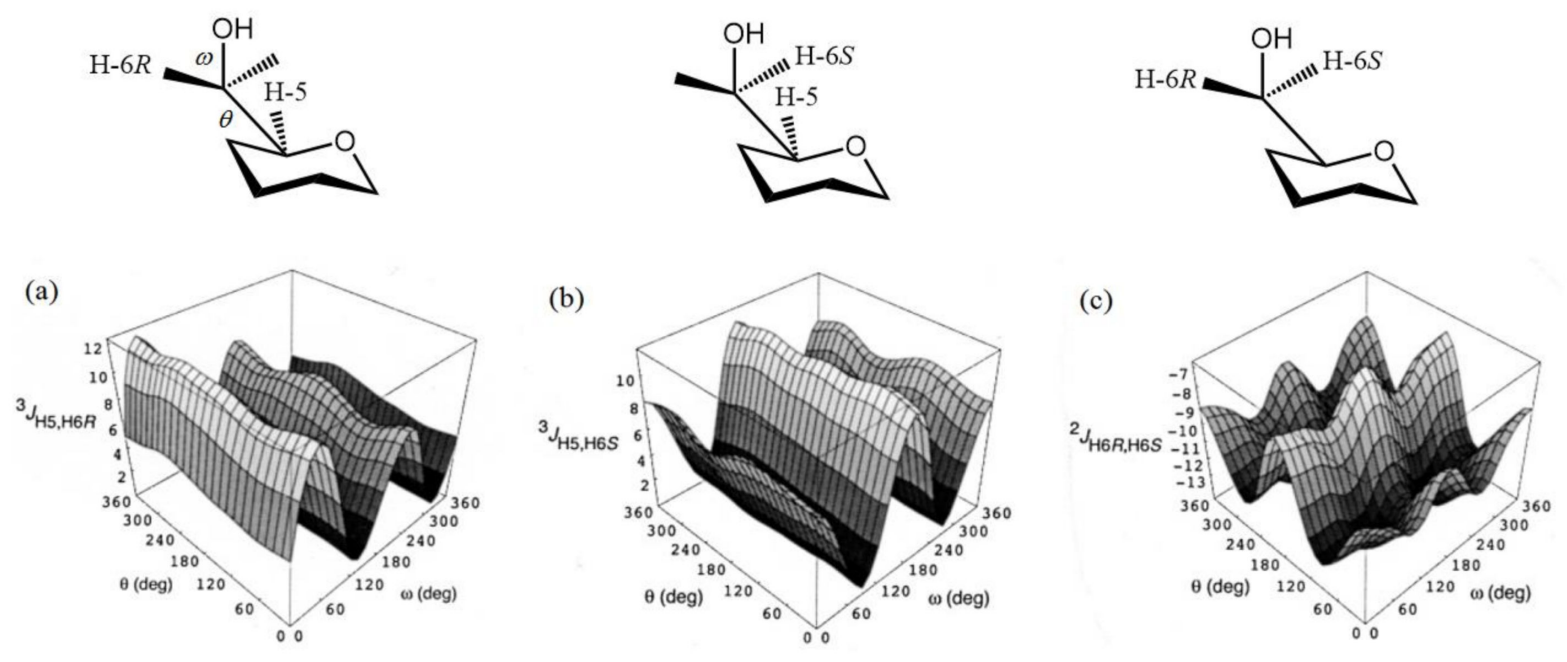
Hypersurfaces of ω and θ dihedral angles calculated for (**a**)—^3^*J*_H5,H6*R*_, (**b**)—^3^*J*_H5,H6*S*_, and (**c**)—^2^*J*_H6*R*,H6*S*_ in the restricted compound modelling hexopyranoside cycle. Reproduced with minor editing privilege from Thibaudeau et al. [127] with the permission of the American Chemical Society.

In a more recent paper by Thibaudeau et al. [127], the dihedral angle dependencies of ^2^*J*_C,H_ and ^3^*J*_C,H_ in the compound modelling hexopyranoside cycle, (see Figure 22) were established at the B3LYP/6-31G(d) level and performed calculations were substantiated by measuring those couplings in the ^13^C-labeled 4,6-*O*-ethylidene derivatives of D-glucose and D-galactose with known constrained dihedral angles. In this respect, methyl gluco- and galactopyranosides **34**–**37** shown in Scheme 4 were prepared as the singly ^13^C-enriched samples. The latter were used for the experimental measurement of their geminal and vicinal *J*_C,H_ and *J*_C,C_ couplings involving carbons and hydrogens near to the hydroxymethyl group, as compared to the performed calculations.

**Figure 23:**
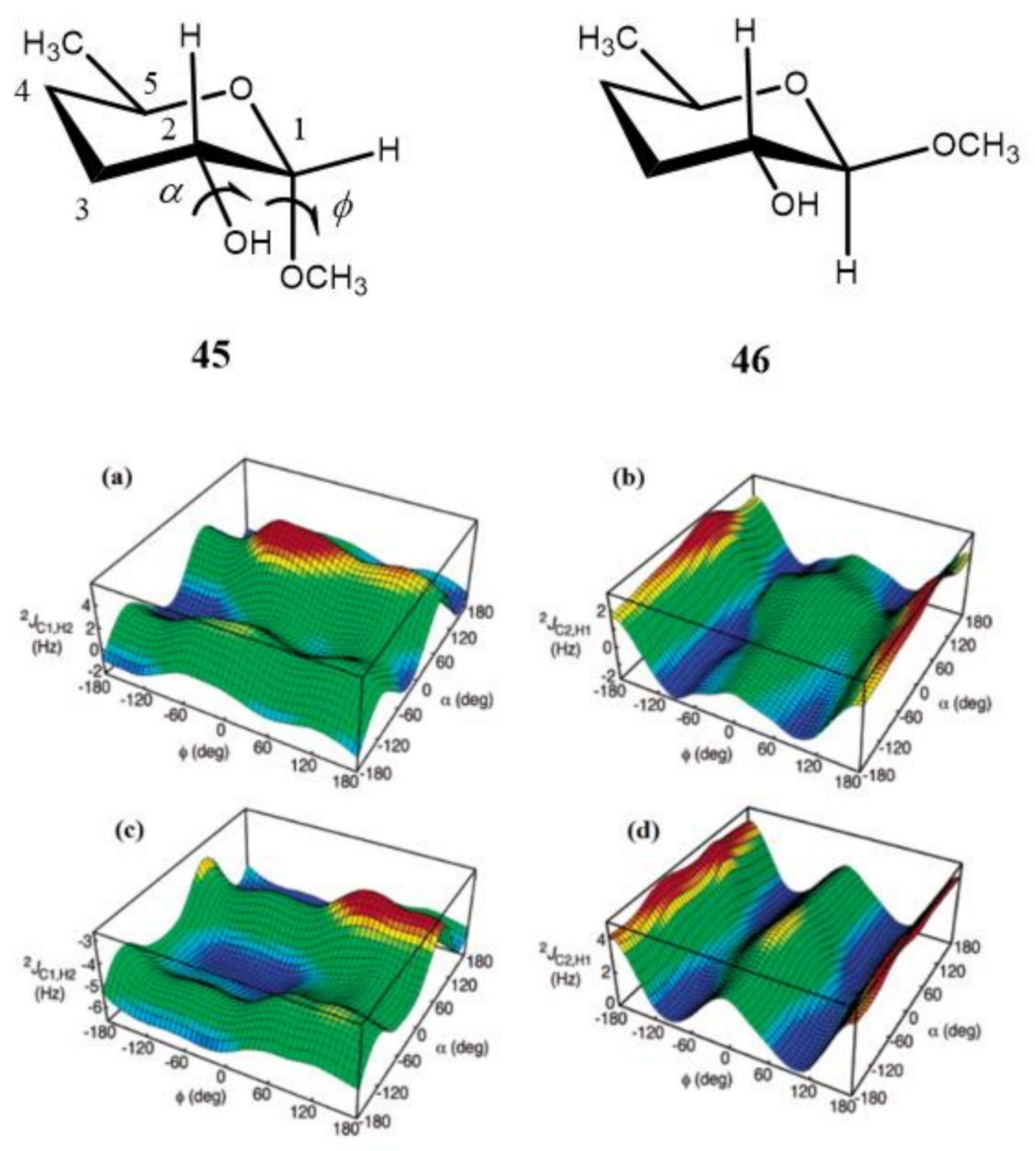
Computed hypersurfaces showing dependencies of geminal carbon-hydrogen couplings on the dihedral angles φ and α for: (**a**)—^2^*J*(C-1,H-2) and (**b**)—^2^*J*(C-2,H-1) in compound **45**; (**c**)—^2^*J*(C-1,H-2) and (**d**)—^2^*J*(C-2,H-1) in compound **46**. Reproduced from Klepach et al. [128] with the permission of the American Chemical Society.

It was found by the same principal authors [128] that ^2^*J*(C-2,H-1) of the aldopyranosyl ring in carbohydrates displayed a systematic dependence on the dihedral angles in glycosides regardless of the relative configuration at C^1^ and C^2^. This finding followed from the computed hypersurfaces in the model compounds **45** and **46** showing the dihedral angle dependences of geminal carbon-hydrogen couplings presented in Figure 23. It was also demonstrated that the effect of the C–O bond rotation on ^2^*J*(C-1,H-2) and ^2^*J*(C-2,H-1) of the aldopyranosyl rings depended significantly on the oxygen lone pair orientation providing essential stereochemical dependence. In general, reported trends in carbon-hydrogen coupling constants of pyranoses suggested their important application to the conformational studies of the glycosidic C–O torsion angles of saccharides.

**Figure 24:**
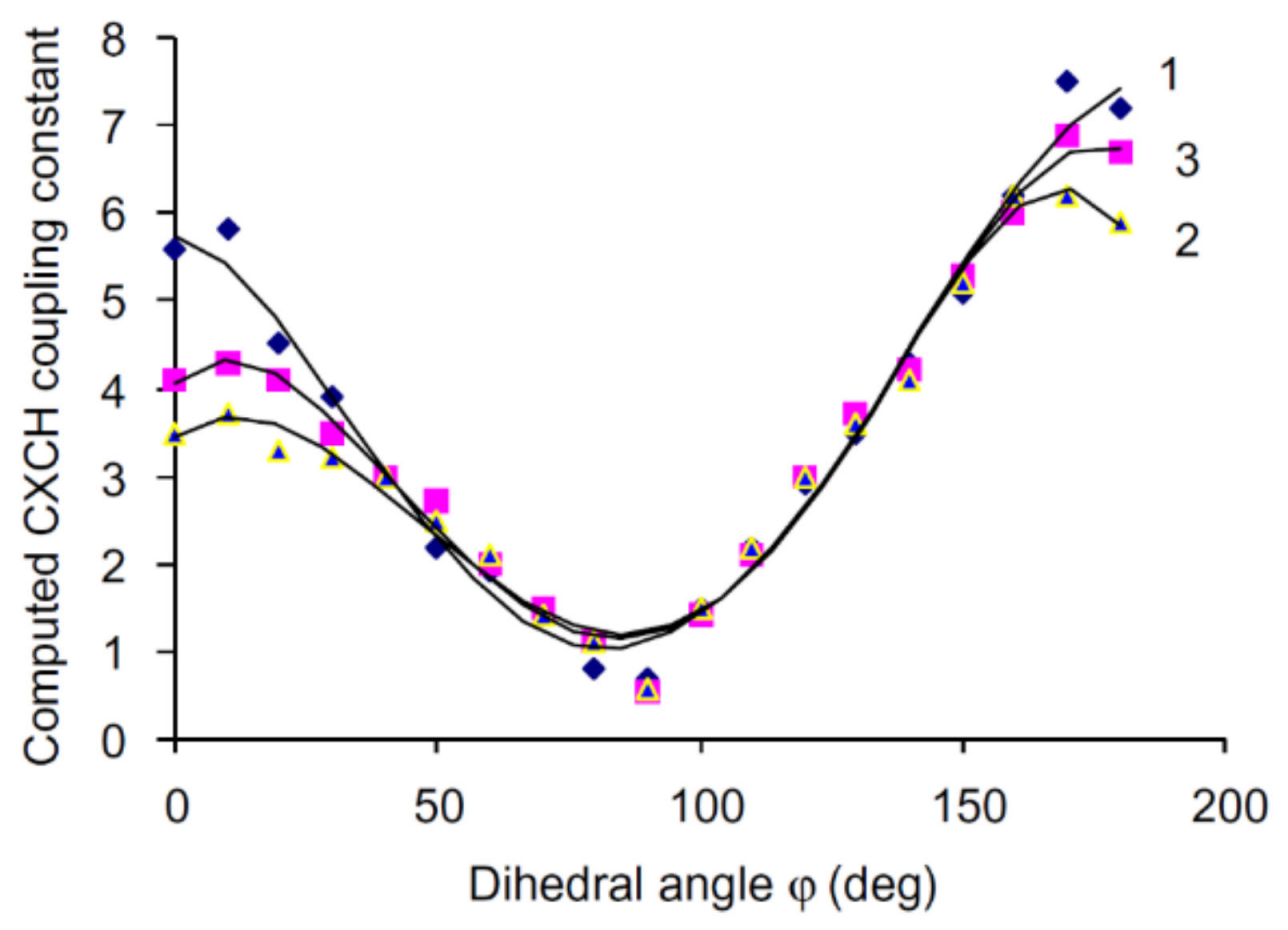
Dihedral angle dependence of vicinal carbon-hydrogen coupling constants calculated at the B3LYP/6-311++G(d,p) level in (**1**)—*O*-glycosides; (**2**)—thioglycosides; (**3**)—glycosides. Reproduced from Tafazzoli and Ghiasi [129] with the permission of Elsevier.

It has long been established that vicinal carbon-hydrogen couplings followed a well-known Karplus dependence on the dihedral angle. To test this dependence between ^3^*J*_C,H_ and torsion angle around the anomeric center of carbohydrates, the conformations of 24 derivatives of glucose and galactose were scrutinized by Tafazzoli and Ghiasi [129]. The authors performed calculations of ^3^*J*_C,H_ in that series at the B3LYP level using the 6-311++G(d,p) basis set with taking into account solvent effects of water within the IEF-PCM approach. It was found that those couplings provided a classical Karplus-type dependence with a well-defined minimum at φ ≈ 90°, as illustrated in Figure 24 for glycosides, *O*-glycosides and thioglycosides.

Carbon-carbon coupling constants over one and two bonds (^1^*J*_C,C_ and ^2^*J*_CCC_) were shown to be sensitive to the presence and orientation of the hydroxyl groups attached to the coupled carbons depending on the rotation around central C–C bond, demonstrating their well-defined Karplus-type dependence [130]. The latter originates in the rotation around both C–O bonds reflecting the lone-pair orbital interactions with the C–C bond, which was essentially stronger than the C–C bond rotational effect. A significant new finding of that study was that ^1^*J*_C,C_ depended on the internal rotation around the C–O bond, reaching maximum when the hydroxyl proton was anti to carbon atom and a minimum in gauche conformation.

On the other hand, the ^2^*J*_CCC_ values in a HO–C–C(OH)–C–OH coupling pathway in aldopyranosyl rings depended markedly on the relative orientations of the hydroxyl groups attached to the terminal coupled carbons (configurational effect) [131] and on the orientation of a hydroxyl substituent attached to the central carbon (conformational effect) [132]. In the former paper, interpretation of ^2^*J*_CCC_ values was performed and stereochemical behavior of ^2^*J*_COC_ has also been examined. That study provided results which were found to be useful in the conformational analysis of the O-glycosidic linkages in oligosaccharides. Performed calculations of ^2^*J*_C,C_ values in compounds modelling aldopyranosyl rings [131,132] were found to be in a fair agreement with available experiment.

**Figure 25:**
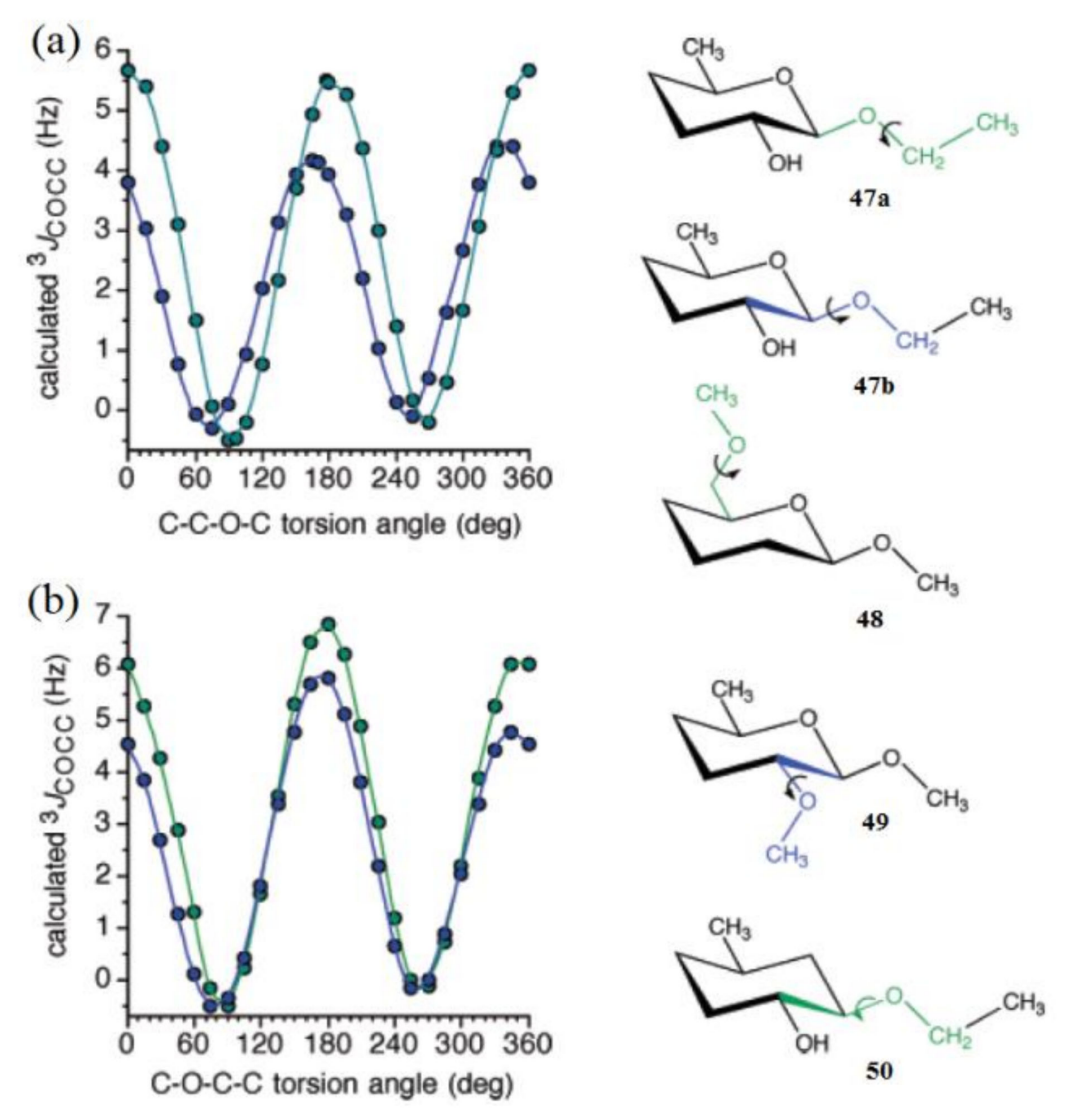
(**a**)—Calculated ^3^*J*_COCC_ values in compounds **47a** (green symbols) and **47b** (blue symbols) as a function of the central C–O bond torsion. Pertinent coupling pathways are highlighted in green in **47a** and blue in **47b**. (**b**)—Calculated ^3^*J*_COCC_ values in compounds **48** (green symbols) and **49** (blue symbols) as a function of the central C−O bond torsion. The corresponding coupling pathways are highlighted in green in **48** and in blue in **49**. Reproduced with minor editing privilege from Zhao et al. [133] with the permission of the American Chemical Society.

Much later, Zhao et al. [133] calculated the dependence of ^3^*J*_COCC_ on the central bond torsion angle in the model structures **47–50** shown in Figure 25. For structures **47a** and **47b**, the overall shapes of the two curves were the same, but two differences were noteworthy: curve amplitude was slightly reduced for compound **47b**, and more importantly, the curve was phase shifted to the left, yielding maximum at about 165°, as compared to the dihedral angle of 180° for compound **47a** (Figure 25a). The C−O−C−C coupling pathway in **3** involved an exocyclic primary alcoholic carbon, whereas that in **4** corresponded to an alternate pathway involving C^1^-C^2^ cyclic bond, and both curves showed a coupling maximum at about 180° (Figure 25b).

Concerning the C−O−C−C coupling pathways, the curve amplitude was found to be slightly larger for compound **48** as compared to **49**. An illustrative comparison was made between the structures **47b** and **50** where both coupling pathways were identical**,** except that in **50** the ring oxygen was absent, and thus the pathway lacked an internal electronegative substituent. The corresponding curve for compound **5** displayed a maximum at about 180°, as was observed also for **47a**, **48**, and **49**, which provided evidence that internal electronegative substituents were responsible for the observed phase shifting.

**Scheme 5:**
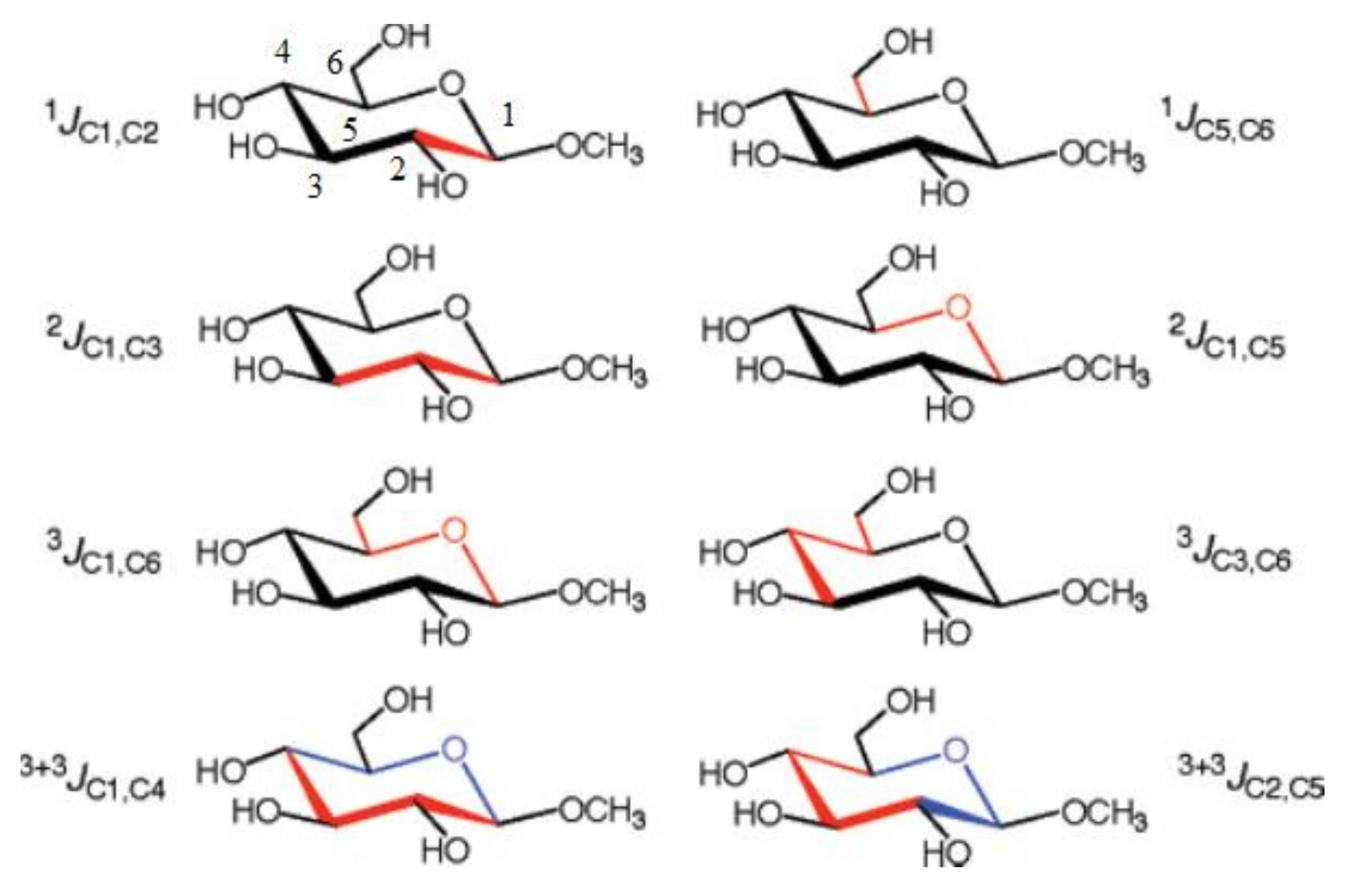
Representative ^1^*J*_C,C_, ^2^*J*_C,C_, ^3^*J*_C,C_, and ^3+3^*J*_C,C_ coupling constants in methyl α-D-glucopyranoside. The corresponding coupling pathways are shown in red and in red and blue for the dual-pathway coupling constants. Reproduced with minor editing privilege from Bose-Basu et al. [134] with the permission of the American Chemical Society.

More recently, Bose-Basu and coauthors [134] performed a systematic study of stereochemical behavior and structural factors of the one-bond, geminal, and vicinal carbon-carbon coupling constants in the series of aldohexopyranoses and aldohexopyranosides, exemplified below with the representative coupling pathways (related to the corresponding coupling constants) in methyl α-D-glucopyranoside, (Scheme 5). All calculations were performed at the DFT level with a classical B3LYP functional using the original [5s2p1d|3s1p] basis set [135] on B3LYP/6-31G(d) geometries.

In the course of that study [134] it was found that generally geminal couplings, ^2^*J*_C,C_, depended on the orientation of the C‒O bonds attached to the terminal coupled carbons. At this, ^2^*J*_CCC_ coupling constants (those transmitted across carbon atom) were shown to be also affected by the intervening carbon origin and, on the other hand, by the internal rotation around the C−O bond. Vicinal ^3^*J*(C-1,C-6) and ^3^*J*(C-3,C-6) couplings demonstrated a classical Karplus-type dependence, but also were affected by the in-plane terminal hydroxyl substituents. For both types of couplings, rotation around the terminal C^5^-C^6^ bond affected vicinal coupling due to the alternating in-plane and out-of-plane oxygen atom O^6^. The values of ^3^*J*(C-3,C-6) were shown to be dependent on the configuration at the C^4^ atom. Both vicinal couplings, ^3^*J*(C-1,C-6) and ^3^*J*(C-3,C-6), were influenced by the remote stereochemical effects involving C^1^ and C^3^ atoms. As a general result of that survey, new structural correlations have been found for ^2^*J*(C-3,C-5), which, like ^3^*J*(C-3,C-6), demonstrated a “remote” dependence on the configuration at the anomeric center. Finally, investigation of the dual pathway ^13^C−^13^C couplings, ^3+3^*J*(C-1,C-4) and ^3+3^*J*(C-2,C-5), revealed an important general internal electronegative substituent effect on vicinal carbon-carbon couplings in saccharides.

**Figure 26:**
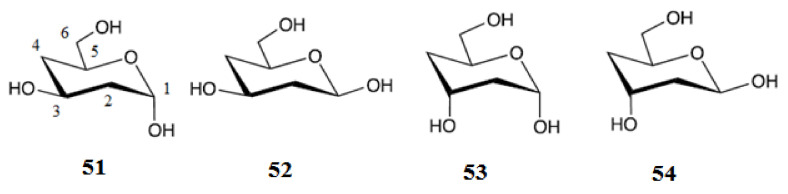
Four model carbohydrates used for the establishment of the dihedral angle dependencies of ^3^*J*(C-1,C-6) and ^3^*J*(C-3,C-6) coupling constants.

**Figure 27:**
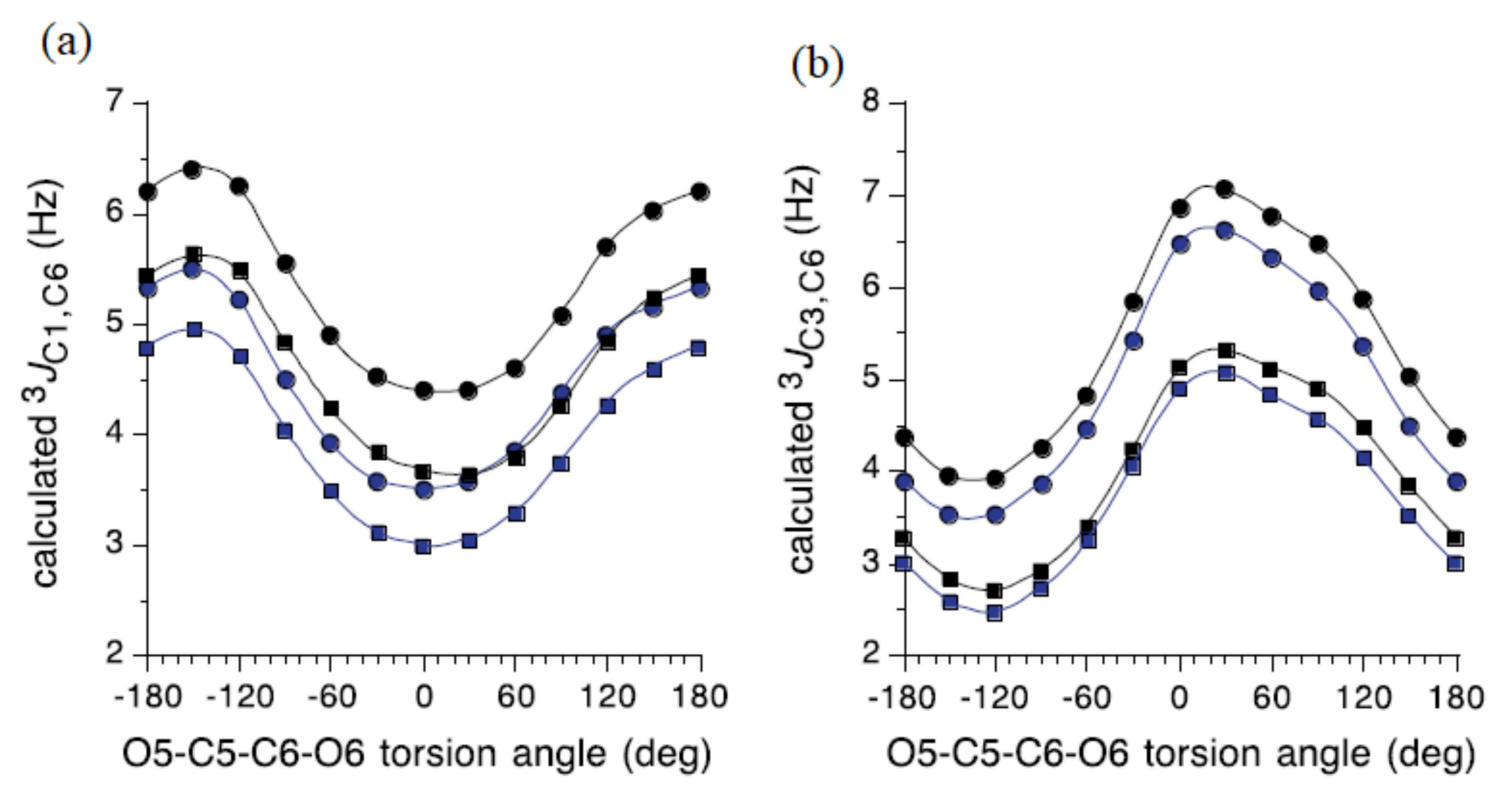
(**a**)—The effect of the exocyclic CH_2_OH conformation on calculated ^3^*J*(C-1,C-6) coupling constant in the model carbohydrates **51** (blue circles), **52** (black circles), **53** (blue squares), and **54** (black squares); (**b**)—The effect of the exocyclic CH_2_OH conformation on calculated ^3^*J*(C-3,C-6) values, same symbols. Reproduced from Bose-Basu et al. [134] with the permission of the American Chemical Society.

The influence of the C^5^−C^6^ bond conformation on ^3^*J*(C-1,C-6) and ^3^*J*(C-3,C-6) for the model carbohydrates **51–54** (Figure 26) reached at by Bose-Basu et al. [134] is shown in Figure 27. It was found that ^3^*J*(C-1,C-6) was minimal at the O^5^−C^5^−C^6^−O^6^ dihedral angle of 0° and maximal at 180°, as shown in Figure 27a. For this dependence, the curve amplitudes were about 2 Hz, confirming the effect of the O^6^ orientation on ^3^*J*(C-1,C-6). On the other hand, results of the DFT calculations of ^3^*J*(C-3,C-6) shown in Figure 27b demonstrated that this coupling was maximal at the O^5^−C^5^−C^6^−O^6^ dihedral angles of 60° (O^6^ in-plane) and -120° (C^4^ and O^6^ eclipsed). These computational data also revealed the dependence of ^3^*J*(C-3,C-6) on the anomeric configuration, with α-anomers yielding slightly smaller couplings, in agreement with the experimental findings. It was also established that configuration at the C^3^ carbon influenced the ^3^*J*(C-1,C-6) value, while the configuration at C^1^ influenced the ^3^*J*(C-3,C-6) coupling constant.

**Figure 28:**
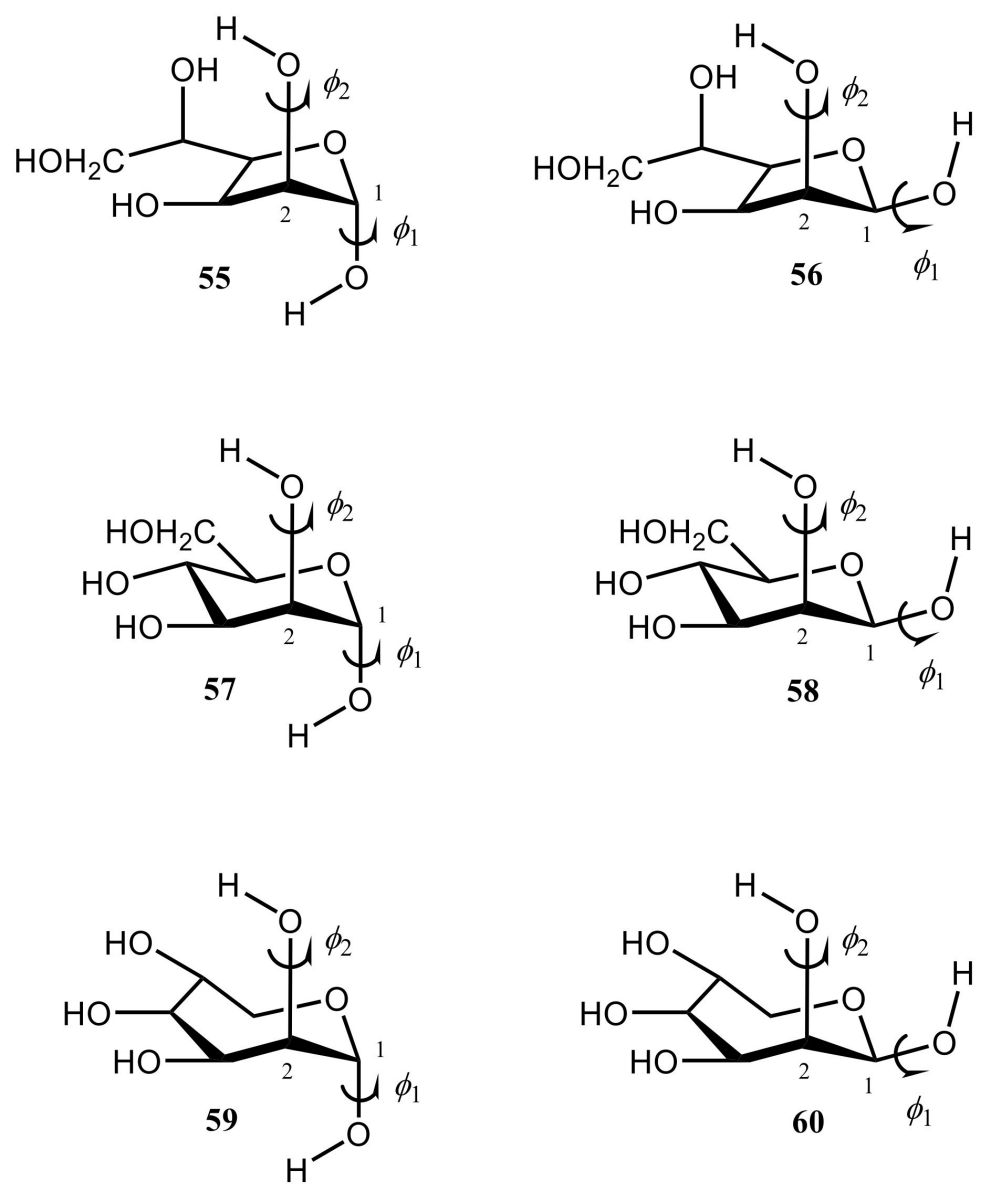
Structural formulas of α- and β-D-mannofuranoses (**55** and **56**), α- and β-D-mannopyranoses (**57** and **58**), and α- and β-D-mannoseptanoses (**59** and **60**).

**Figure 29:**
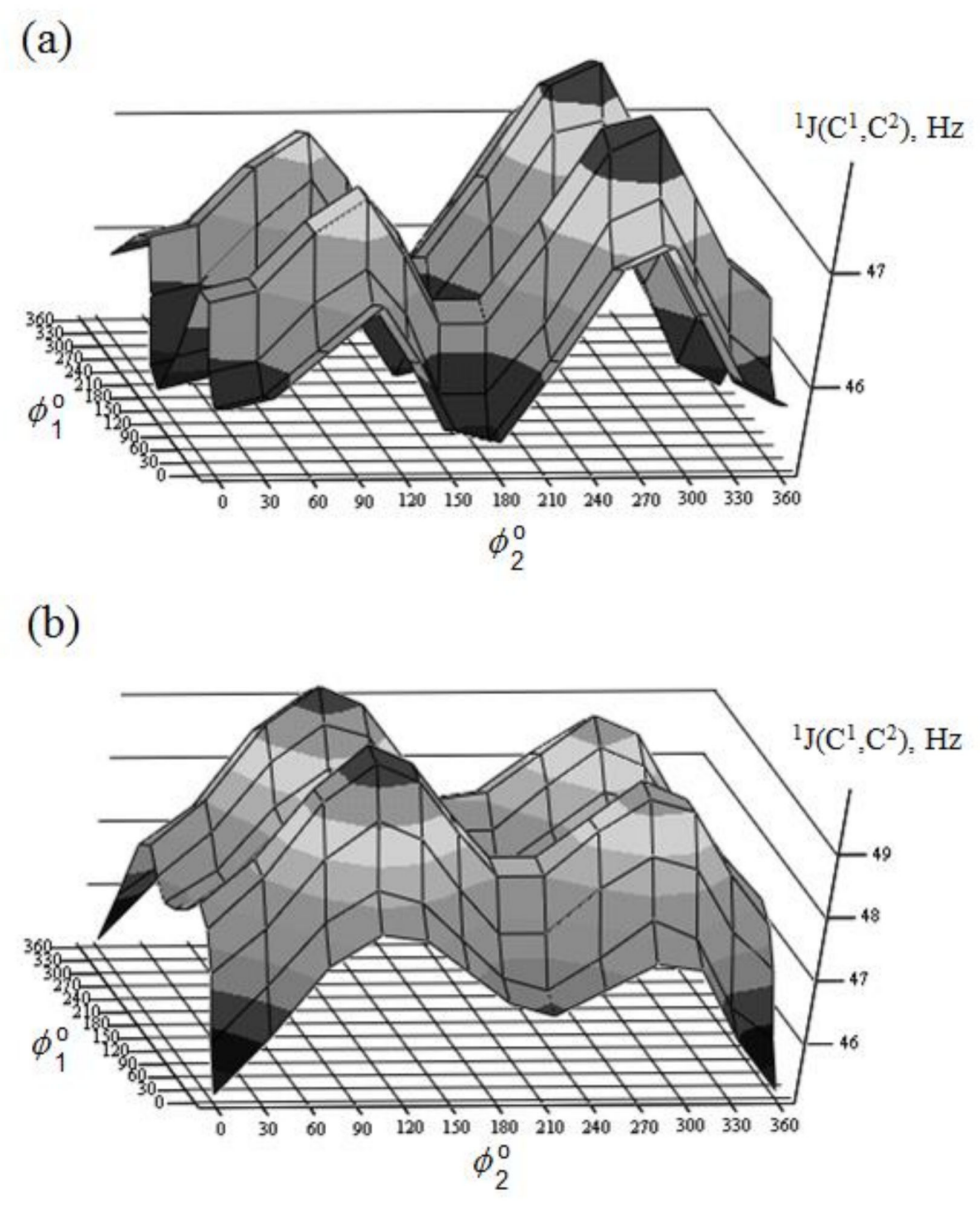
Rotational surface of ^1^*J*(C-1,C-2) of (**a**)—α-D-mannofuranose and (**b**)—β-D-mannofuranose. Reproduced from the PhD Thesis by Danilova [136].

**Figure 30:**
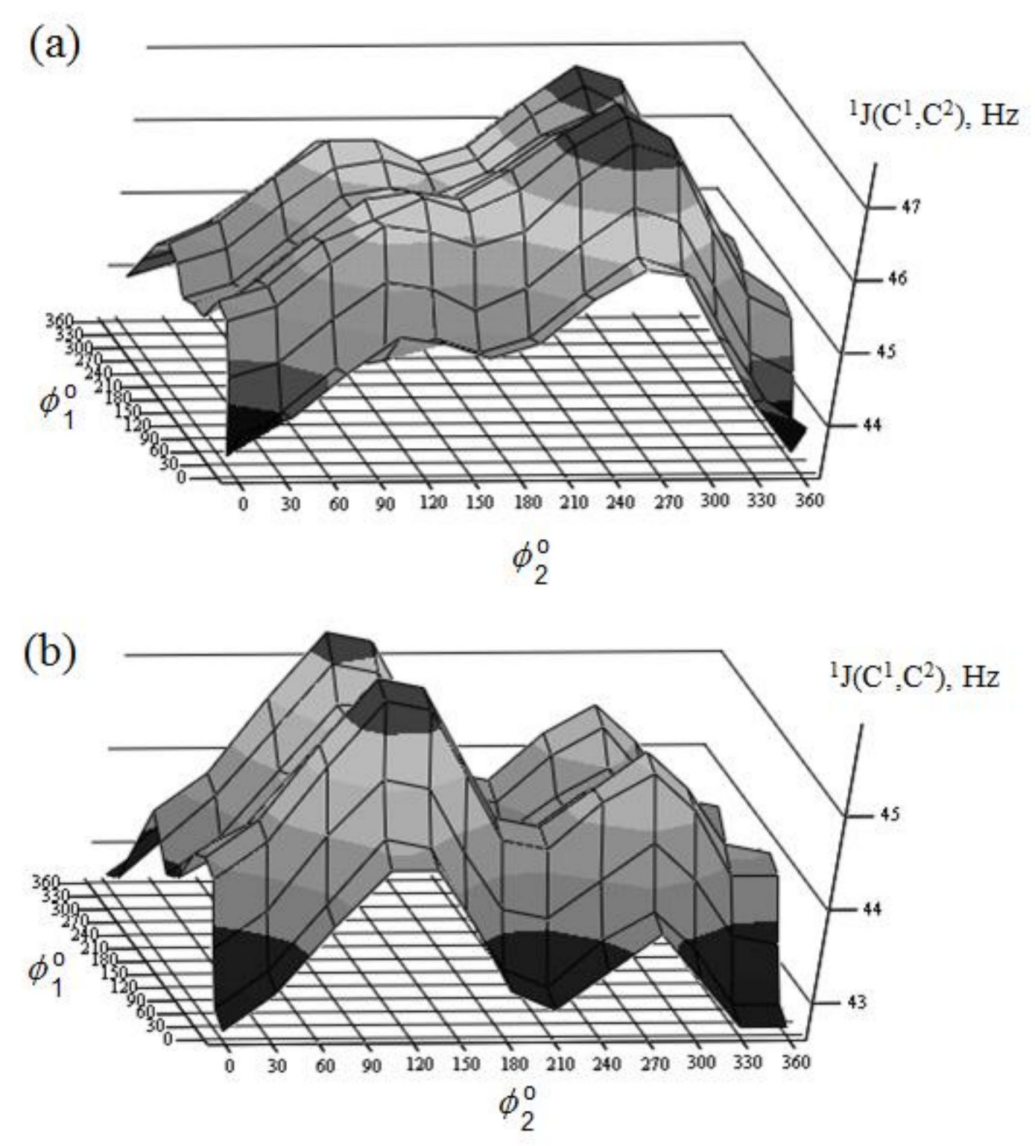
Rotational surface of ^1^*J*(C-1,C-2) of (**a**)—α-D-mannopyranose and (**b**)—β-D-mannopyranose. Reproduced from the PhD Thesis by Danilova [136].

**Figure 31:**
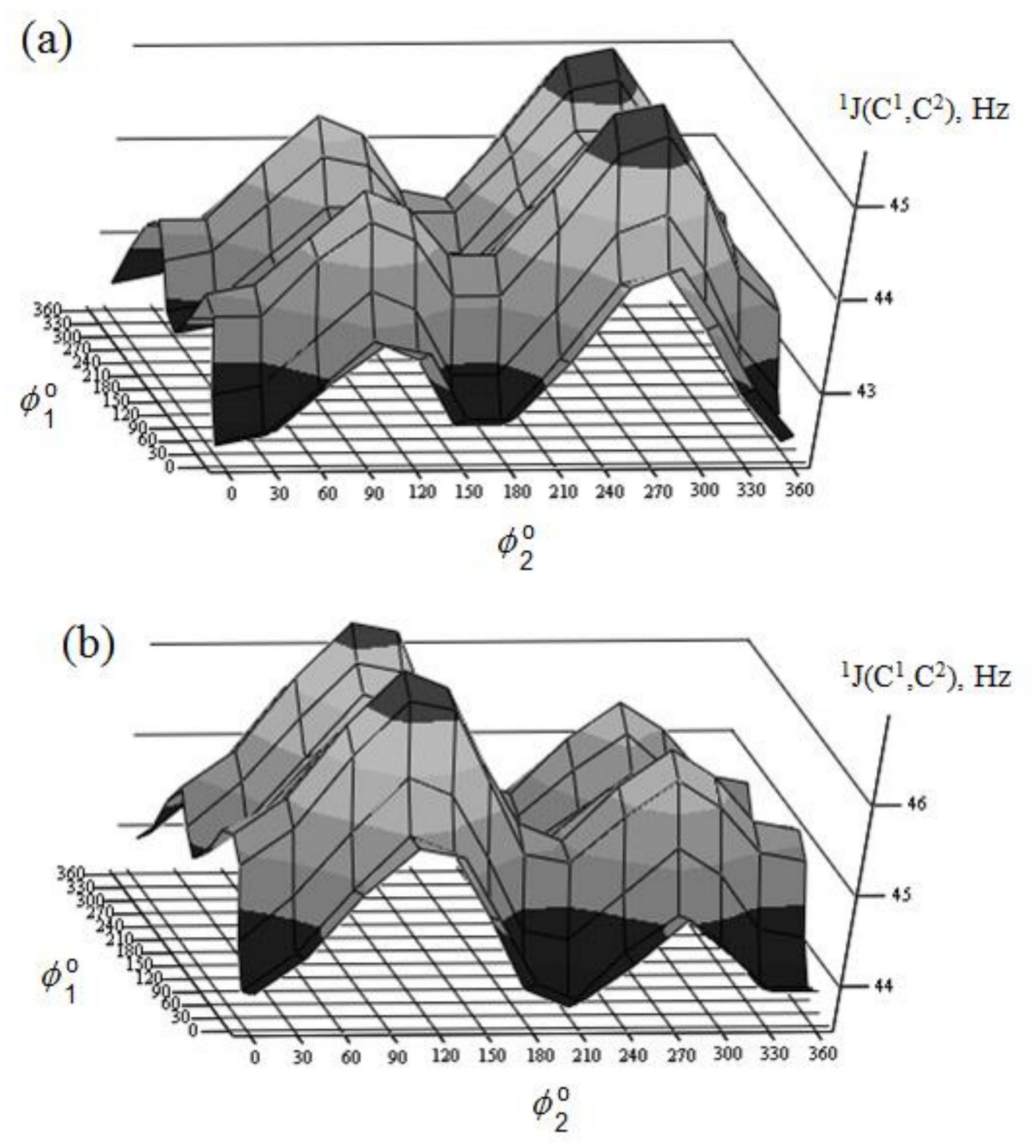
Rotational surface of ^1^*J*(C-1,C-2) of (**a**)—α-D-mannoseptanose and (**b**)—β-D-mannoseptanose. Reproduced from the PhD Thesis by Danilova [136].

Danilova et al. [136,137,138,139,140] performed scrupulous SCPT INDO study of the rotational surfaces of ^1^*J*_1,2_ carbon-carbon coupling constants in the series of α- and β-D-mannofuranoses (**55** and **56**), α- and β-D-mannopyranoses (**57** and **58**), and α- and β-D-mannoseptanoses (**59** and **60**), see Figure 28, Figure 29, Figure 30 and Figure 31.

All rotational surfaces were characterized by the well-defined four maxima and four minima, which was due to the fact that rotation of the hydroxyl groups exercised an appreciable effect on ^1^*J*(C-1,C-2) coupling constants in the furanose, pyranose, and septanose forms of carbohydrates. Undoubtedly, those dependences originated in the well-known oxygen lone pair effect on ^1^*J*_C,C_ [141], which should be taken into account in the conformational analysis of the cyclic forms of carbohydrates based on the stereospecificity of carbon-carbon coupling constants involving anomeric carbon. Stereochemical dependences of ^1^*J*(C-1,C-2) of monosaccharides are illustrated in Table 3 for the D-forms of the most common hexopyranoses.

A nice agreement of calculated couplings with experiment was observed in that study. As one can see in Table 3, the range of calculated ^1^*J*(C-1,C-2) coupling constants depends on the stereochemical structure of the pyranose ring varying within ca. 5 Hz from 43.4 Hz in α-D-allose to 48.7 Hz in α-D-idose. It should be emphasized that any data on experimental measurement of ^1^*J*(C-1,C-2) relates to the equilibrium of the normal and alternative conformers (shifted to one of the forms in each particular case), so that a correct comparison of calculated and experimental values could be performed only when one of the conformers essentially dominates.

## 4. Di- and Polysaccharides

We start this section with the discussion of the most illustrative example of heparin oligomers. It is well known that heparin, a well-known glycosaminoglycan, is composed of the repeated disaccharide sequences of L-iduronic acid and D-glucosamine linked through the (1→4)-glycosidic bonds. Heparin itself is known mainly for its anticoagulant properties and its biological activity, which is due to its unique pentasaccharide sequence. A number of papers, mainly those produced by Hricovíni and coauthors (which are referenced and discussed below), are devoted to the computational, structural, and conformational studies of heparan sulfate glycosaminoglycans. The latter are the linear polyanions containing dimeric repeating units of hexosamine and uronic acid. In fact, the sequence diversity that characterizes heparin and heparan sulfate is generally due to a series of distinct disaccharides combined in the oligomer unit. Those structures arise from different combinations of twelve possible α-D-glucosamine residues with one of the four possible β-D-gluco- or α-L-idopyranuronic acids. The sequence differences are mainly due to the combinations of sulfated substituents in the 2-*O*-, 3-*O*-, and 6-*O*-endo and -exo positions in the cycles of uronic acid or glucosamine residues. As a matter of fact, heparin itself contains 2-*O*-sulfated iduronic acid in excess of 60% of the total uronic acids. Those basic features of heparin oligomers are discussed in much detail in the early review by Guerrini, Hricovíni, and Torri [142].

**Scheme 6:**
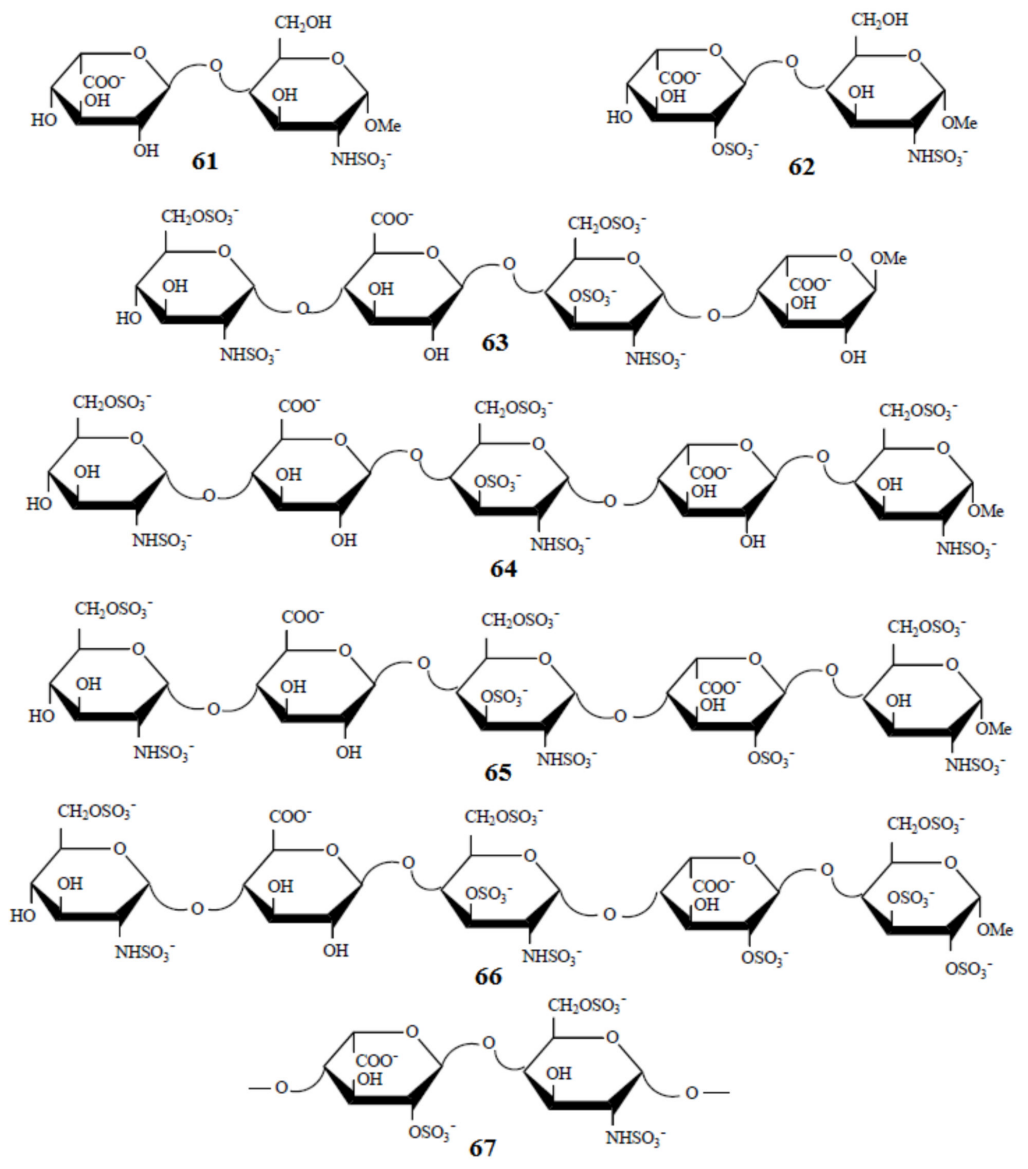
Heparin oligosaccharides containing IdoA residue differently substituted and located in distinct sequences. Reproduced with minor editing privilege from Guerrini [142] with the permission of Bentham Science Publishers Ltd.

Of particular interest are the heparin oligosaccharides containing idose residue differently substituted and located in distinct sequences, as illustrated in Scheme 6 for the representative dimeric (**61** and **62**), tetrameric (**63**), pentameric (**64**, **65**, and **66**), and polymeric (**67**) heparin oligosaccharides studied by Hricovini and coauthors. To differentiate those structures, computed and experimental proton-proton coupling constants in the idose residue in several heparin oligosaccharides were established to be highly informative in respect of the structural recognition of different forms of heparin. In the non-sulfated structures, the ^3^*J*_H,H_ couplings were found to be relatively small, and this fact was in agreement with the predominance of the ^1^*C*_4_ form. On the other hand, considerably larger couplings supported the preference of the ^2^*S*_0_ form of some heparin oligosaccharides.

**Figure 32:**
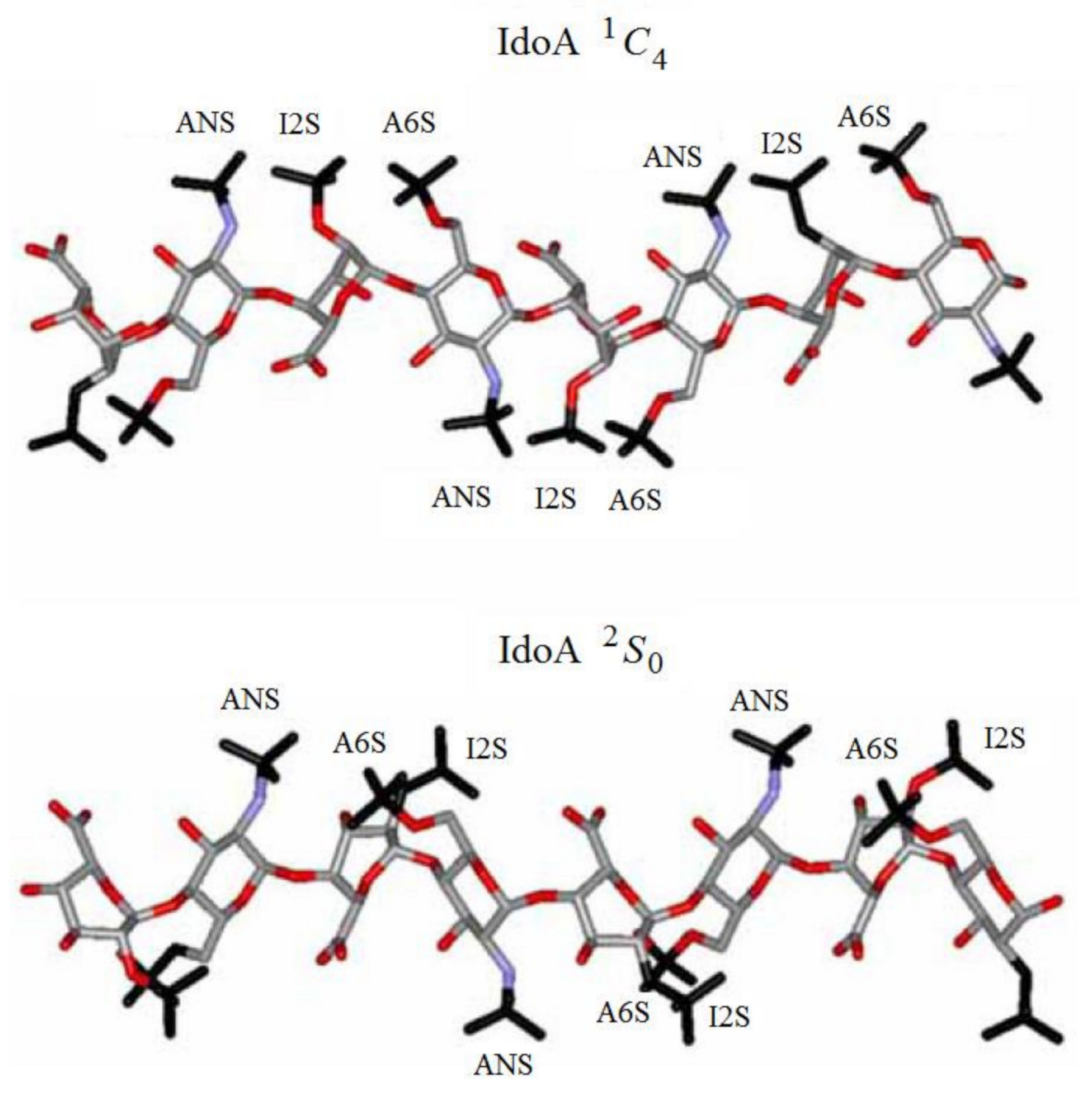
Structural differences of heparin chains associated with distinct conformations of iduronic acid residues. For designations on the figure, see original publication. Reproduced with minor editing privilege from Guerrini [142] with the permission of Bentham Science Publishers Ltd.

Indeed, a strong influence of the sulfation of the neighbouring residues was evident in the chemically modified oligosaccharides, where conformational equilibrium was shifted nearly completely towards the skewed ^2^*S*_0_ form. On the other hand, in the ^1^*C*_4_ conformation, the sulfate groups are more dispersed, covering a relatively wide range of space on two sides of the molecule. As a result, all sulfate groups in the ^2^*S*_0_ form are arranged into four well-resolved, symmetrically oriented clusters as shown in Figure 32.

**Figure 33:**
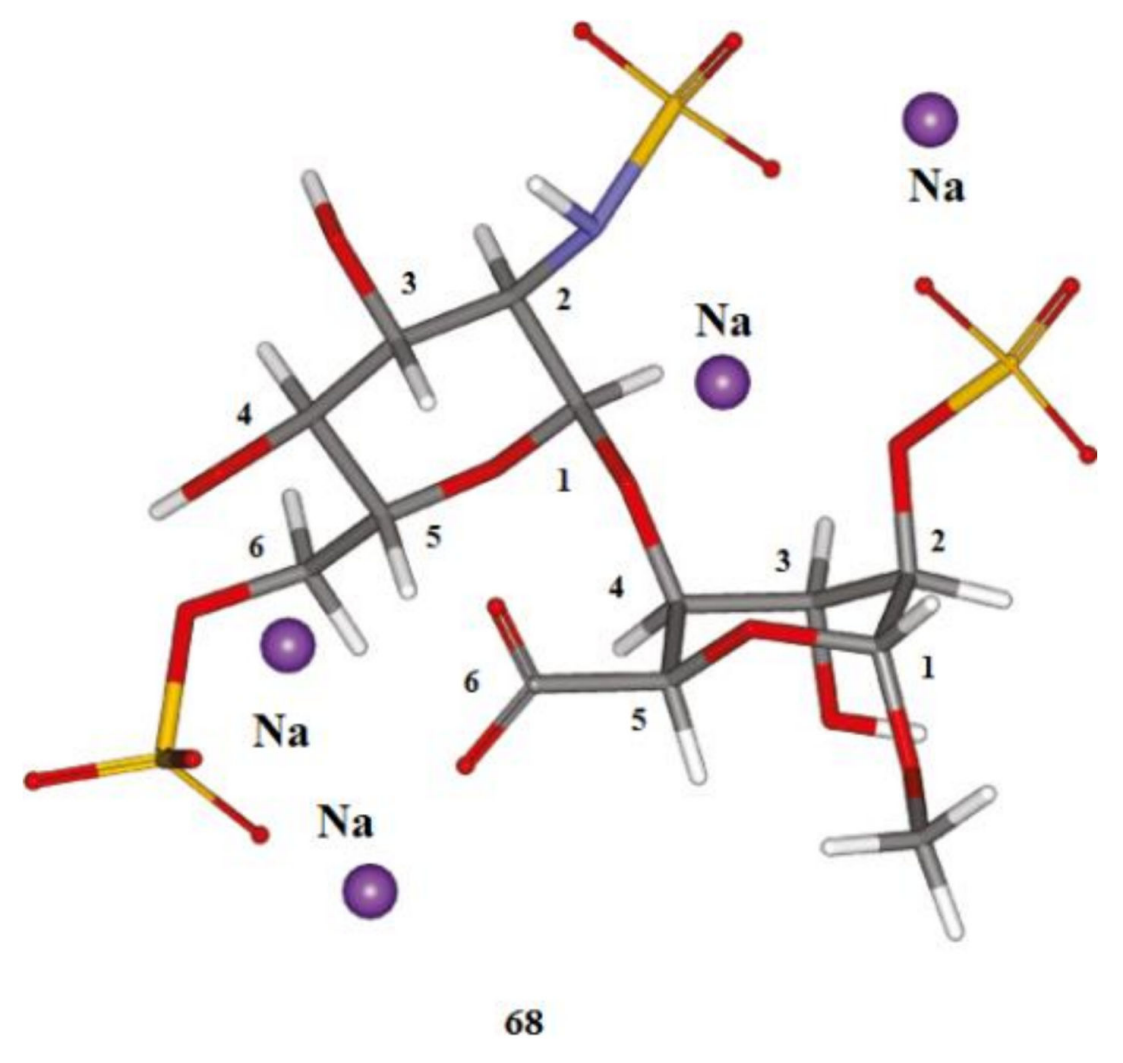
The structure of heparin disaccharide—methyl *O*-(2-deoxy-2-sulfamino-6-*O*-sulfo-α-D-glucopyranosyl)-(1→4)-2-*O*-sulfo-α-L-idopyranoside uronate tetrasodium salt. Element colors: hydrogen—light gray, carbon—gray, nitrogen—violet, oxygen—red, sulphur—yellow. Reproduced with minor editing privilege from Hricovíni [144] with the permission of the American Chemical Society.

To begin with, we will shortly concentrate on the publications provided by Hricovíni and coauthors on the computational NMR studies of different heparin oligosaccharides. In this line, Hricovíni and coauthors [143,144] performed B3LYP/6-311++G(d,p) calculation of vicinal ^1^H-^1^H and one-bond ^13^C-^1^H spin-spin coupling constants of heparin disaccharide, which is methyl *O*-(2-deoxy-2-sulfamino-6-*O*-sulfo-α-D-glucopyranosyl)-(1→4)-2-*O*-sulfo-α-L-idopyranoside uronate tetrasodium salt (**67**) shown in Figure 33. Calculations in that study were performed at the DFT level using B3LYP and M05-2X functionals in combination with Pople’s 6-311++G(d,p) basis set. Solvent effects were treated by the use of multiple water molecules included into computational space in an explicit way. Interesting to note that solvent-induced variations of the computed indirect one-bond carbon-proton coupling constants were found to be of up to 17 Hz (!) between isolated and solvated states. Performed calculations indicated that only two chair forms contributed to the conformational equilibrium of heparin disaccharide.

**Figure 34:**
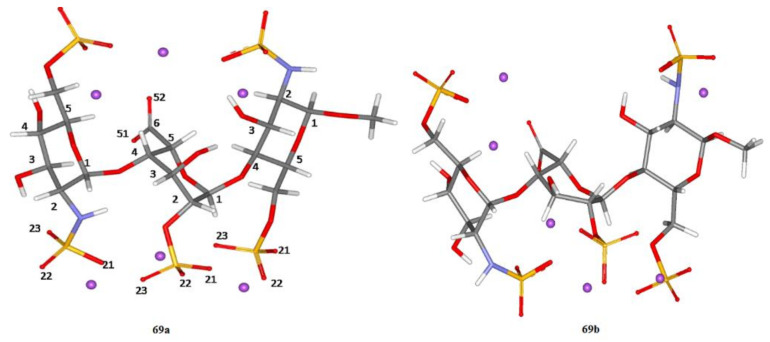
Structure of heparin trisaccharide A. The two forms, **69a** and **69b**, correspond to the different conformations of the IdoA2S residue (central residue). In **69a**, the IdoA2S residue is in the ^1^*C*_4_ conformation; in **69b**, the IdoA2S residue is in the ^2^*S*_0_ conformation. Both GlcN,6S residues are in the ^4^*C*_1_ conformation. Violet dots represent sodium ions. Solvent (water) molecules are not shown for clarity. Reproduced from Hricovíni and coauthors [145] with the permission of the American Chemical Society.

In the next paper [145] from this series, the fully optimized structures of two heparin trisaccharide forms **69a** and **69b** (differing from each other in the conformation of the central iduronic acid residue) were obtained at the B3LYP/6-311+G(d,p) level, as shown in Figure 34. In that study, solvent effects were taken into account either via a continuum solvent model or in an explicit way with putting of as many as 57 molecules of water into calculation space.

Reported vicinal proton-proton coupling constants were evaluated with using various functionals (B3LYP and M0-2X) and basis sets (6-311+G(d,p), TZVP, and DGDZVP) and then compared to their experimental values. It followed that the double zeta DGDZVP basis set performed well for most coupling constants and even outperformed (in the meaning of deviation from experiment) the triple zeta TZVP basis set. In fact, this relatively simple basis set provided most satisfactory data, nearly as good as the data obtained by more rigorous approach based on the 6-311+G(d,p) basis set. Computed vicinal proton-proton coupling constants ^3^*J*_H,H_ in heparin trisaccharide showed that the best agreement with experiment was achieved when Pople’s 6-311+G(d,p) basis set was employed. Other basis sets, less demanding on computer time and memory, DGDZVP and TZVP, also gave acceptable data for most coupling constants. Performed theoretical analysis showed that stereoelectronic effects considerably influenced the ^3^*J*(H-C-C-C) and ^3^*J*(H-C-O-C) values. In particular, it was shown that the effect of oxygen lone pairs in the coupling path had a significant influence on ^3^*J*(H-C-C-C) and ^3^*J*(H-C-O-C) coupling constants differing significantly from each other mainly due to the presence of oxygen in the latter coupling path.

**Figure 35:**
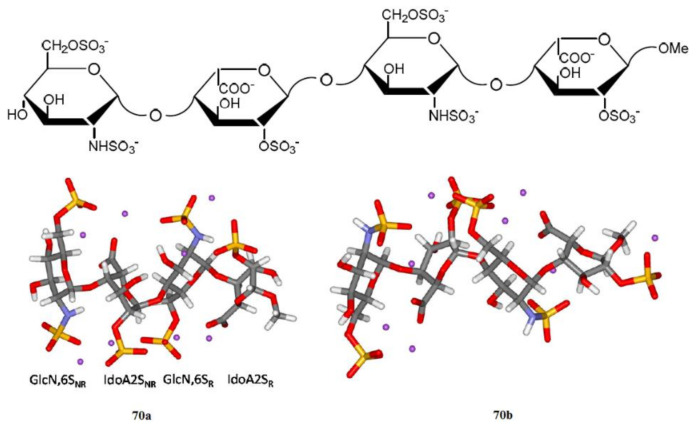
The DFT optimized structure of the heparin tetrasaccharide. The two forms, **70a** and **70b**, have different conformations of the IdoA2S residue. The IdoA2S residues are in the ^1^*C*_4_ conformation in **70a** and in the ^2^*S*_0_ conformation in **70b**. The GlcNS,6S residues are in the ^4^*C*_1_ conformation. Violet dots represent sodium ions. Solvent (water) molecules are not shown for clarity. The fully optimized molecular structures of two predominant tetrasaccharide conformations of the sulphated iduronic acid residue were obtained at the B3LYP/6-311+G(d,p) level and with applying explicit water molecules to simulate the presence of a solvent. Reproduced from Hricovíni and coauthor [147] under an open access Creative Commons Attribution 4.0 International Public License. Vicinal proton-proton coupling constants of heparin tetrasaccharide presented in Table 4 showed that the best agreement of the performed calculations with experiment was obtained with a weighted average of ^1^*C*_4_ and ^2^*S*_0_ conformations of 67:33 for the IdoA2S forms. Detailed analysis of the Fermi-contact terms of ^3^*J*(H-C-C-H) demonstrated that important contributions originated from the lone pairs of the neighbouring oxygen atoms. Performed calculations also showed that magnitude of the diamagnetic spin-orbit contributions were sufficiently large as compared to the total values of proton-proton coupling constants.

Shown in Figure 35 is a related structure of two forms of heparin tetrasaccharide studied by the same principal author with colleagues [146,147]. Performed calculations provided detailed information on molecular structure and spin-spin coupling constants of heparin tetrasaccharide consisting of four units, GlcNS,6S_NR_, IdoA2S_NR_, GlcNS,6S_R_, and IdoA2S_R_, representing the predominant heparin repeating-sequence.

In addition to those results, performed DFT calculations provided insight into the formation of intra- and intermolecular hydrogen bonds and ionic interactions. In particular, it was shown that the H-bond network consisted of intra- and interresidue intramolecular hydrogen bonds that affected the overall molecular structure. Indeed, two interresidue bonds were found in **70a** while neither of those bonds was present in **70b**.

**Figure 36:**
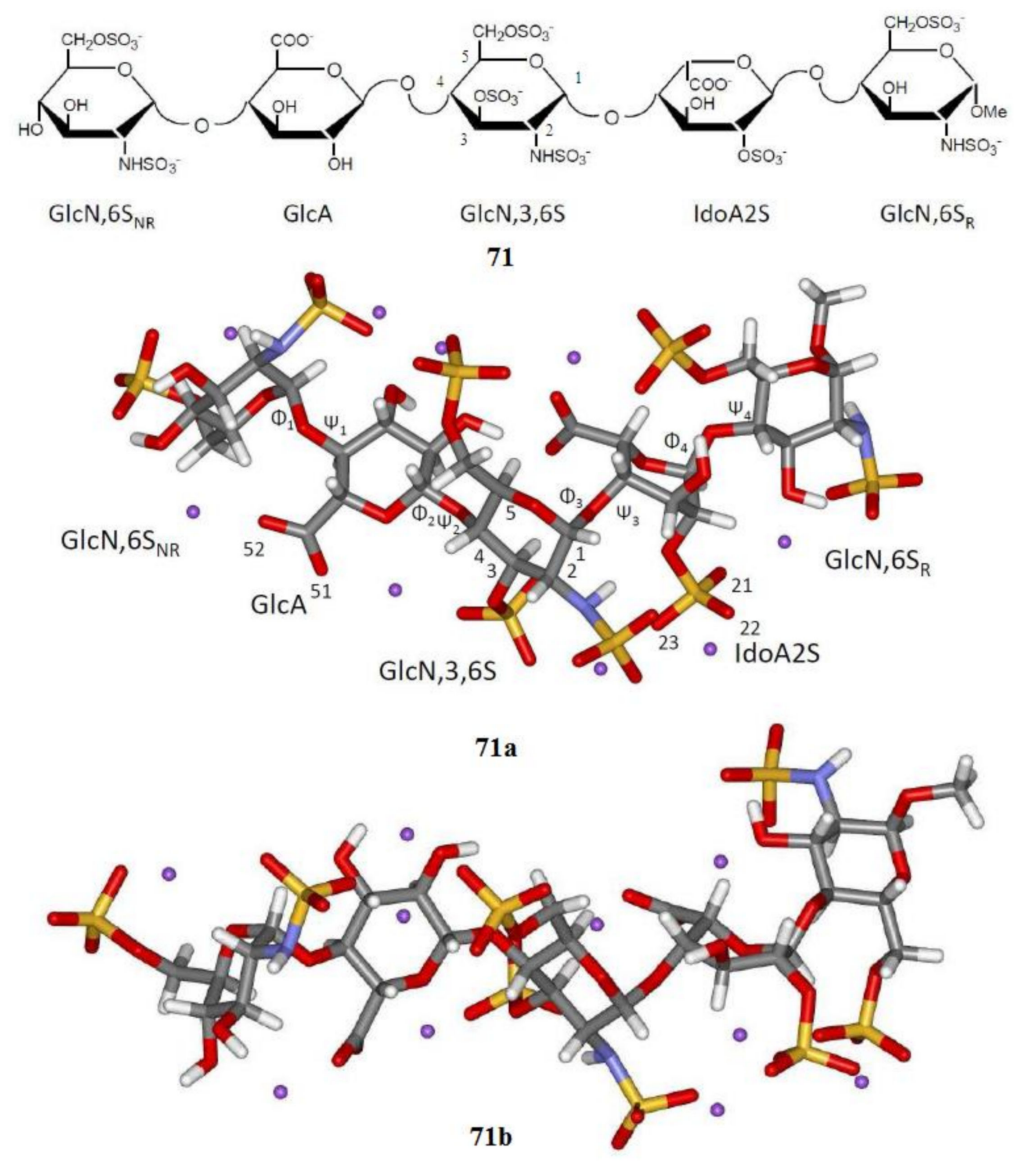
Chemical structure of heparin pentasaccharide (**71**). The IdoA2S residue is in the ^1^*C*_4_ conformation in **71a** and in the ^2^*S*_0_ conformation in **71b**. Other residues are in the ^4^*C*_1_ conformation. Violet dots represent sodium ions. Solvent water molecules are not shown for clarity. Reproduced from Hricovini [148] with the permission of the American Chemical Society.

Finally, performed DFT calculations as compared to experiment were applied to the analysis of heparin pentasaccharide (**71**) shown in Figure 36 [148]. In that paper, the fully optimized molecular geometries of two pentasaccharide conformations **71a** and **71b** (presented in the same figure) were obtained at the B3LYP/6-311+G(d,p) level in the presence of water solvent, the latter included in an explicit form. The averaged vicinal proton-proton coupling constants computed at the DFT level (which are exemplified in Table 5) demonstrated that the best agreement with experiment was obtained with a weighted average of ^1^*C*_4_:^2^*S*_0_ = 15:85 conformations of the sulfated L-iduronic acid forms indicating that this ratio was shifted towards the ^2^*S*_0_ form.

A detailed analysis of calculated spin-spin coupling constants, including their individual contributions, performed in that study [146] demonstrated that the Fermi-contact contributions to ^3^*J*_H,H_ coupling constants arouse not only from the electrons on atoms along the coupling path, but from the lone pairs of oxygen atoms at the glycosidic linkages as well. It was shown that the spin-orbit contributions to some ^3^*J*_H,H_ were comparable in magnitude with their Fermi terms. The values of the diamagnetic spin-orbit terms primarily depended on the contributions including those from molecular orbitals in the neighborhood of the coupled nuclei as well as from the more remote orbitals. For carbon-proton coupling constants, the Fermi-contact terms were the dominant contributions, and the contributions of spin-orbit terms were substantially smaller for most of ^3^*J*_H,H_ couplings.

Geometry optimization in the performed study of heparin pentasaccharide [146] was carried out at the B3LYP/6-311+G(d,p) level, while spin-spin coupling constants were calculated with the same functional and the DGDZVP basis set. The geometry optimization was performed for two conformations of the IdoA2S residue**,** whereas all other residues were adopted in the ^4^*C*_1_ conformation. Solvent effects were taken into account in an explicit way for as many as 98 water molecules in view of the fact that hydration of polar groups in heparin pentasaccharide was better described in that way than with a continuum IEF-PCM model. The initial positions of solvating water molecules were based on coordinates of oxygen atoms in water molecules in the published crystal data of sulfated monosaccharides.

**Figure 37:**
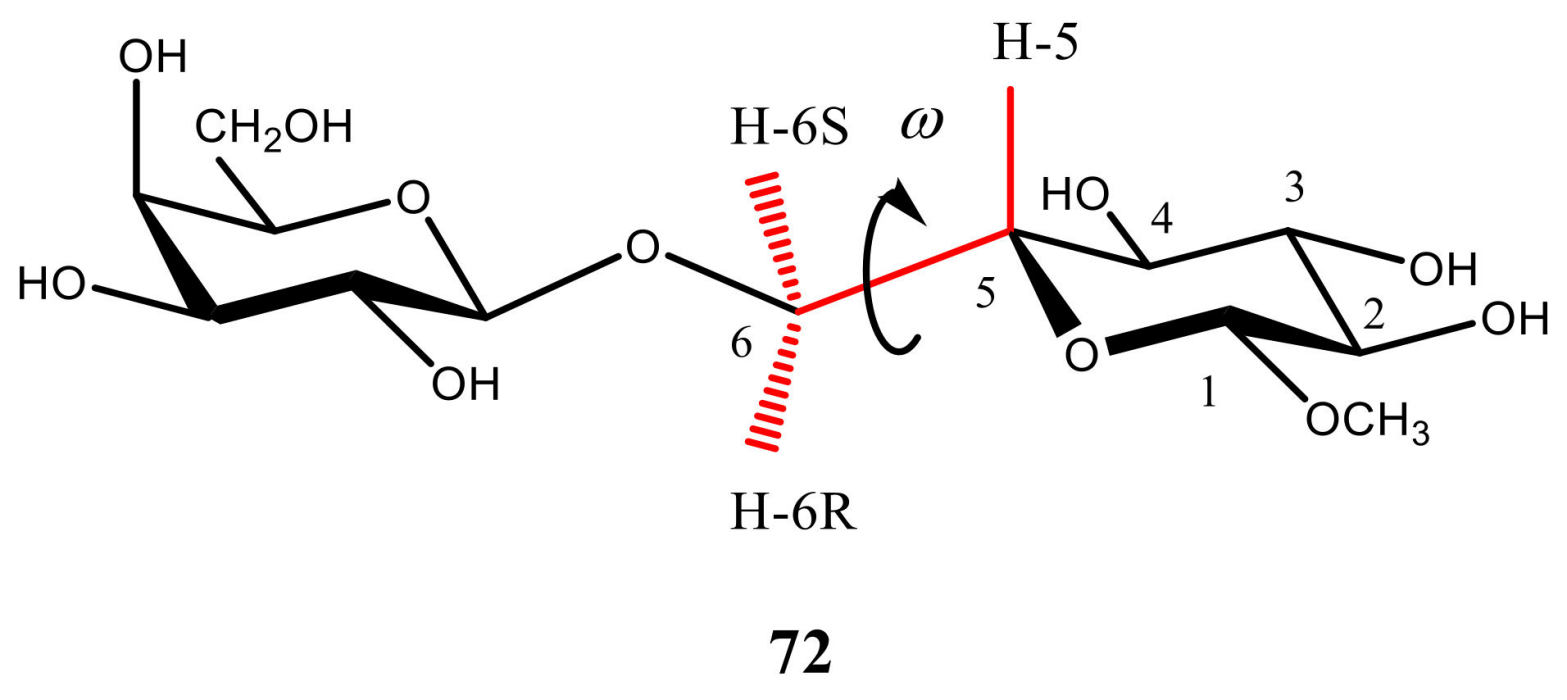
A representative structure of methyl β-D-galactopyranosyl-(1→6)-β-D-glucopyranoside used for the investigation of the Karplus dependence of ^2^*J*(H-6*R*,H-6*S*), ^3^*J*(H-5,H-6*R*), and ^3^*J*(H-5,H-6*S*).

The next illustrative example (which is retrieved from the fundamental review [1]) deals with a marked Karplus dependence of ^2^*J*(H-6*R*,H-6*S*), ^3^*J*(H-5,H-6*R*), and ^3^*J*(H-5,H-6*S*) which could be used to establish conformational behavior of a representative disaccharide, methyl β-D-galactopyranosyl-(1→6)-β-D-glucopyranoside (**72)**, Figure 37, in respect to the internal rotation around its C(5)-C(6) bond.

Indeed, the fractional populations of rotamers dealing with the internal rotation around the C(5)-C(6) bond of **72** could be easily determined through the Karplus analyses of its ^2^*J*(H-6*R*,H-6*S*), ^3^*J*(H-5,H-6*R*), and ^3^*J*(H-5,H-6*S*) coupling constants demonstrating marked Karplus-type behavior. In general, the Karplus-type dependences of ^2^*J*_H,H_ and ^3^*J*_H,H_ in carbohydrates and oligosaccharides enable a straightforward assignment of their rotational conformations based on the DFT calculations of their dihedral angle dependencies in combination with experiment.

**Figure 38:**
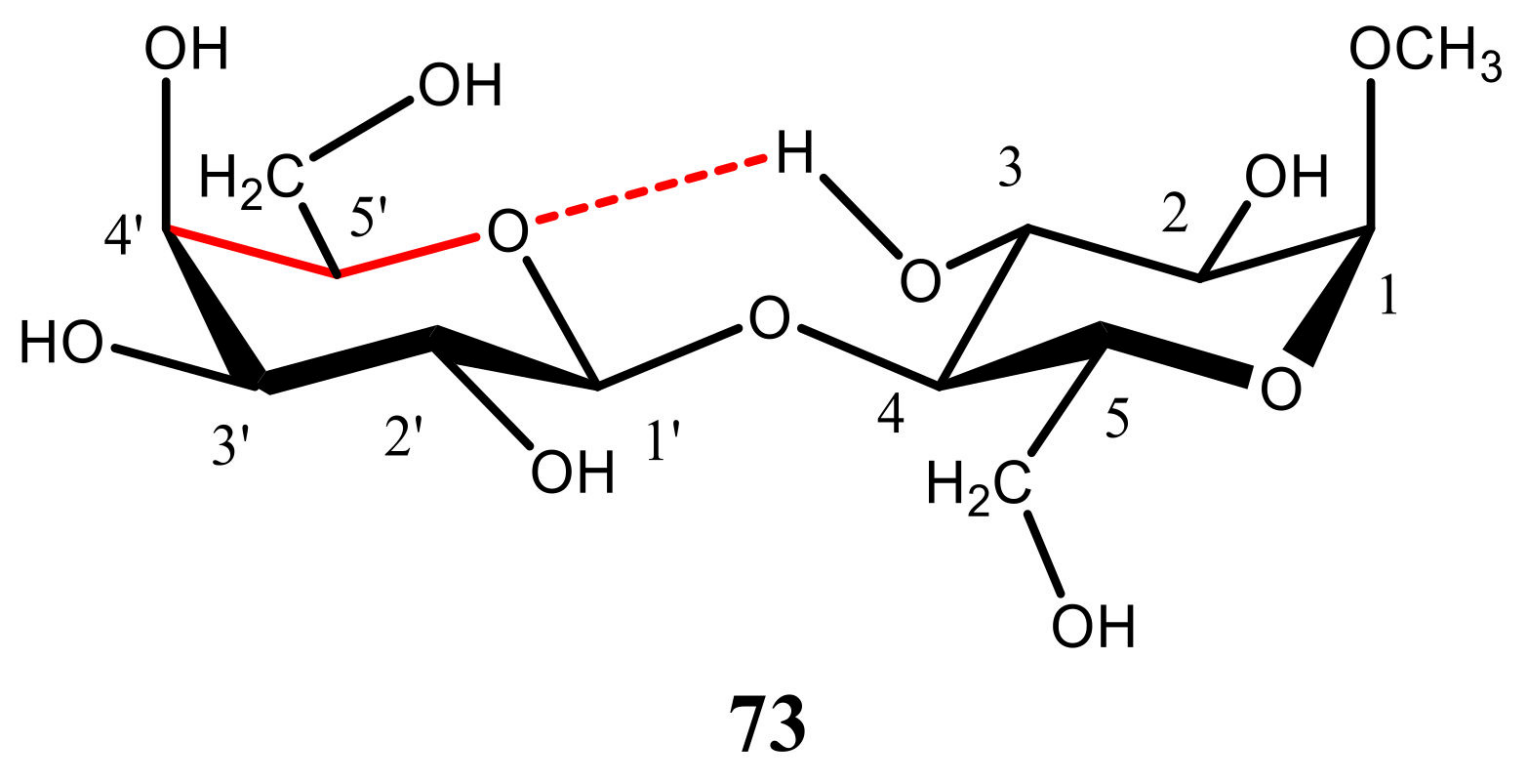
A representative structure of methyl α-lactoside methyl β-D-galactopyranosyl-(1→4)-α-D-glucopyranoside used to find the evidence of the intramolecular hydrogen bonding involving the hydroxyl group at the C^3^ and O atoms of the adjacent pyranosyl ring.

Zhang et al. [149] tried to find the evidence of the intramolecular hydrogen bonding involving the hydroxyl group at the C^3^ and O atoms of the adjacent pyranosyl ring in the experimental and the B3LYP computed values of the inter-ring ^3h^*J*_C-4′,H_ coupling constant, as exemplified below for methyl α-lactoside methyl β-D-galactopyranosyl-(1→4)-α-D-glucopyranoside (**73**). However, computed values of ^3h^*J*_C-4′,H_ in **73** (Figure 38) and related structures appeared to be next to negligible (ca. 0.1 Hz), and no presence of that coupling was observed experimentally.

**Figure 39:**
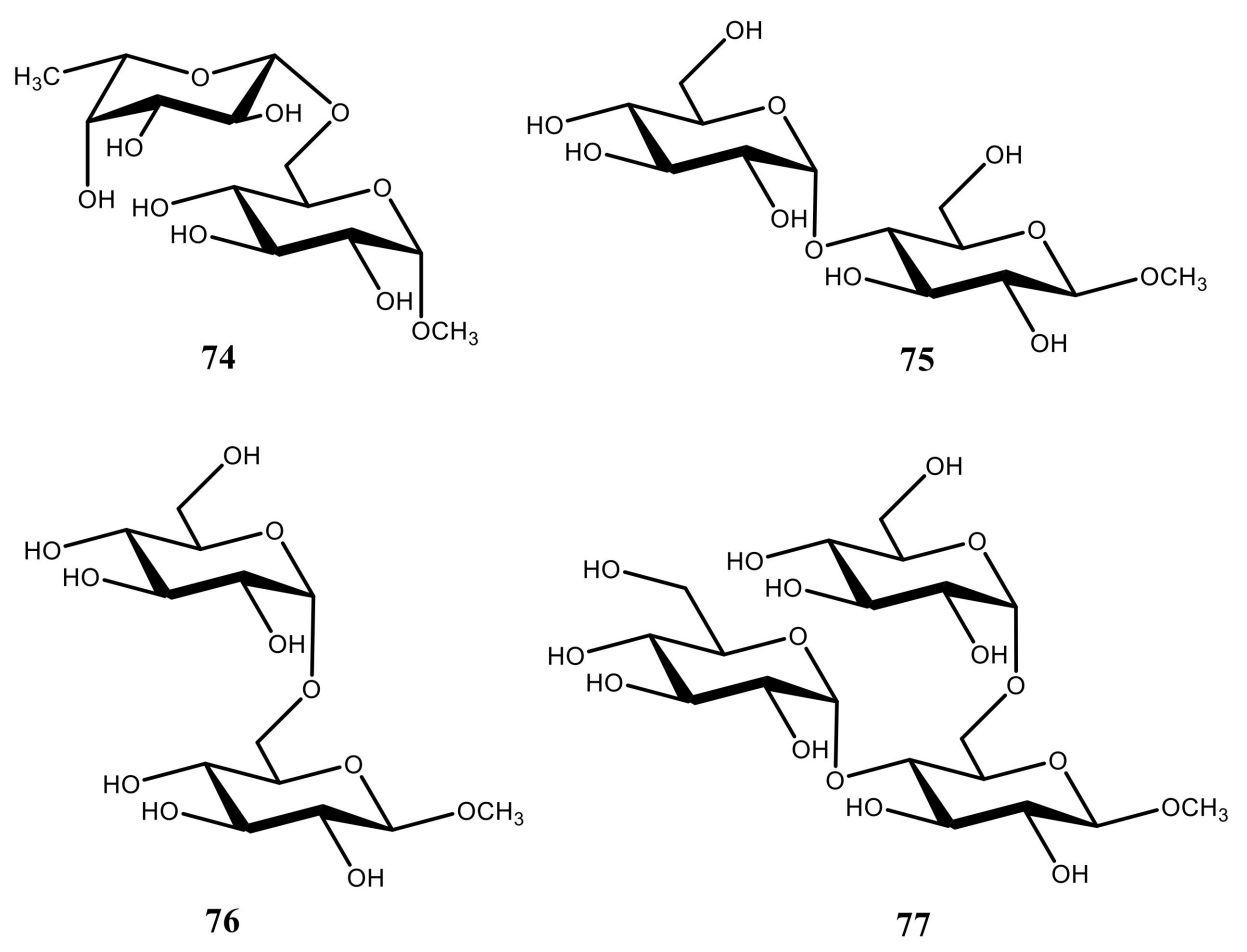
Chemical structures of the representative disaccharides **74**–**76** and trisaccharide **77** providing a preference for the gauche orientation between O^6^ and C^5^.

In one of the early publications by Stenuntz et al. [135], one-bond carbon-hydrogen couplings, ^1^*J*_C,H_, were calculated at the B3LYP/6-31G(d) level to estimate the percentages of the C^5^-C^6^ and C^6^-O^6^ *trans* rotamers in the series of representative disaccharides **74**–**76** and trisaccharide **77** (see Figure 39). It was found that most of those compounds exhibited a preference for the gauche orientation between O^6^ and C^5^, and as the steric requirements of the substituent at O^6^ increased, the percentage of *trans* rotamer had also increased.

Given the variety of different structural factors influencing ^1^*J*_C,H_ in that series, it was not possible to derive an accurate generalized equation relating ^1^*J*_C,H_ in hydroxymethyl fragments of the carbohydrate scaffold to specific molecular parameters. However, despite these limitations, semiquantitative equations for both ^1^*J*(C-6,H-6) couplings (involving two diastereotopic protons) have been established [135]. As a result, based on the computed ^1^*J*_C,H_ couplings and those relationships, it became possible to foresee how the relevant C−H, C−C, and C−O bond lengths change in carbohydrates and saccharides as a function of their conformation.

**Figure 40:**
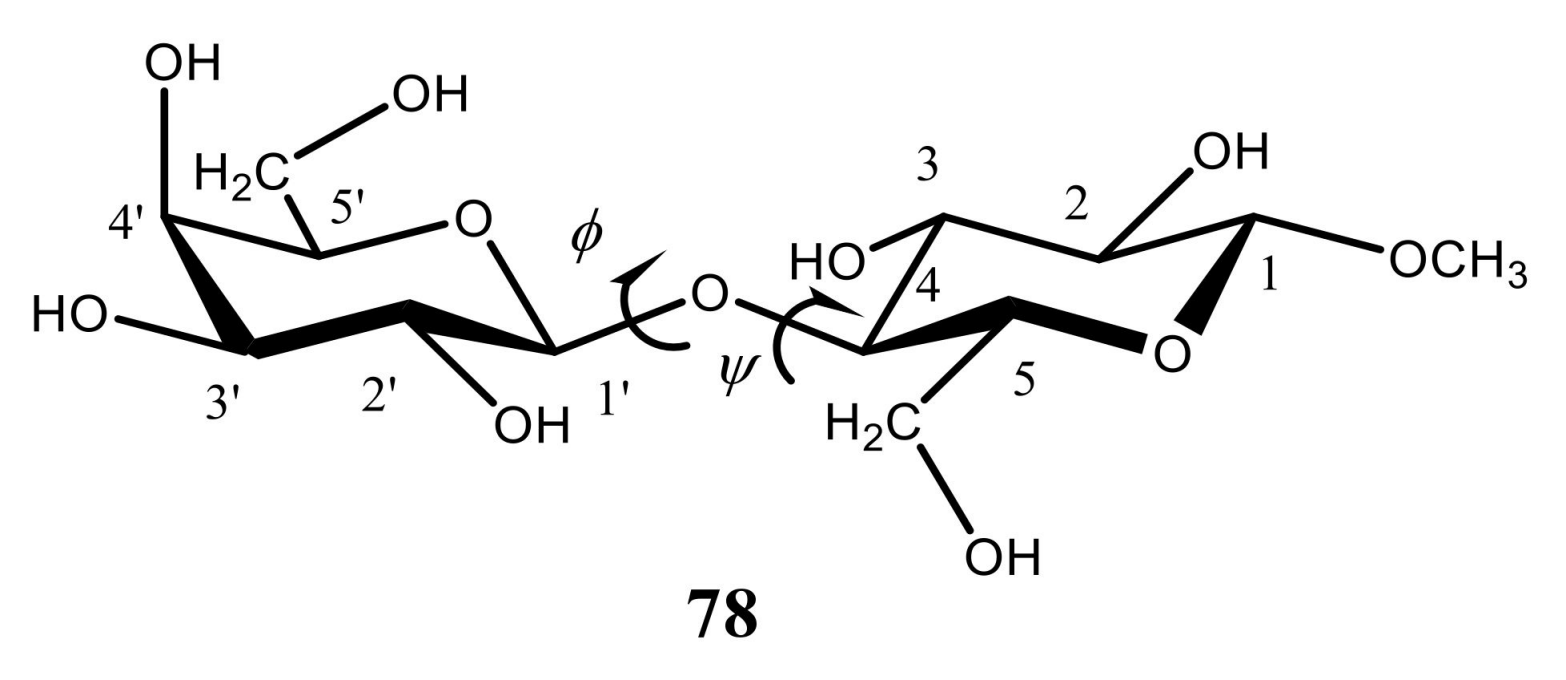
Chemical structure of methyl β-D-galactopyranosyl-(1→4)-β-D-glucopyranoside.

In the recent paper by Zhang et al. [150], aimed at the improving conformational assignments of *O*-glycosidic linkages in oligosaccharides, geminal and vicinal carbon-hydrogen coupling constants across the *O*-glycosidic linkages were investigated at the DFT level. Couplings in a series of twelve structurally related β-(1→4)-linked disaccharides containing systematic covalent modifications in the vicinity of their glycosidic linkages with the most representative methyl β-D-galactopyranosyl-(1→4)-β-D-glucopyranoside (**78**) (Figure 40) were thoroughly analyzed by means of the DFT calculations in comparison with experiment. All calculations of those coupling constants were performed with the B3LYP functional, and the effects of solvent water were accounted for within the Tomasi’s IEF-PCM model. In addition, the dependencies of specific trans-*O*-glycosidic coupling constants on the geometry of the corresponding coupling pathways were evaluated and parameterized at the DFT level. It was found that conformational behavior of those couplings as a function of φ in the studied series of disaccharides was very much the same. At that, the value of ψ varied significantly, allowing a classification of the studied disaccharides based on the preferred linkage conformation in solution.

**Figure 41:**
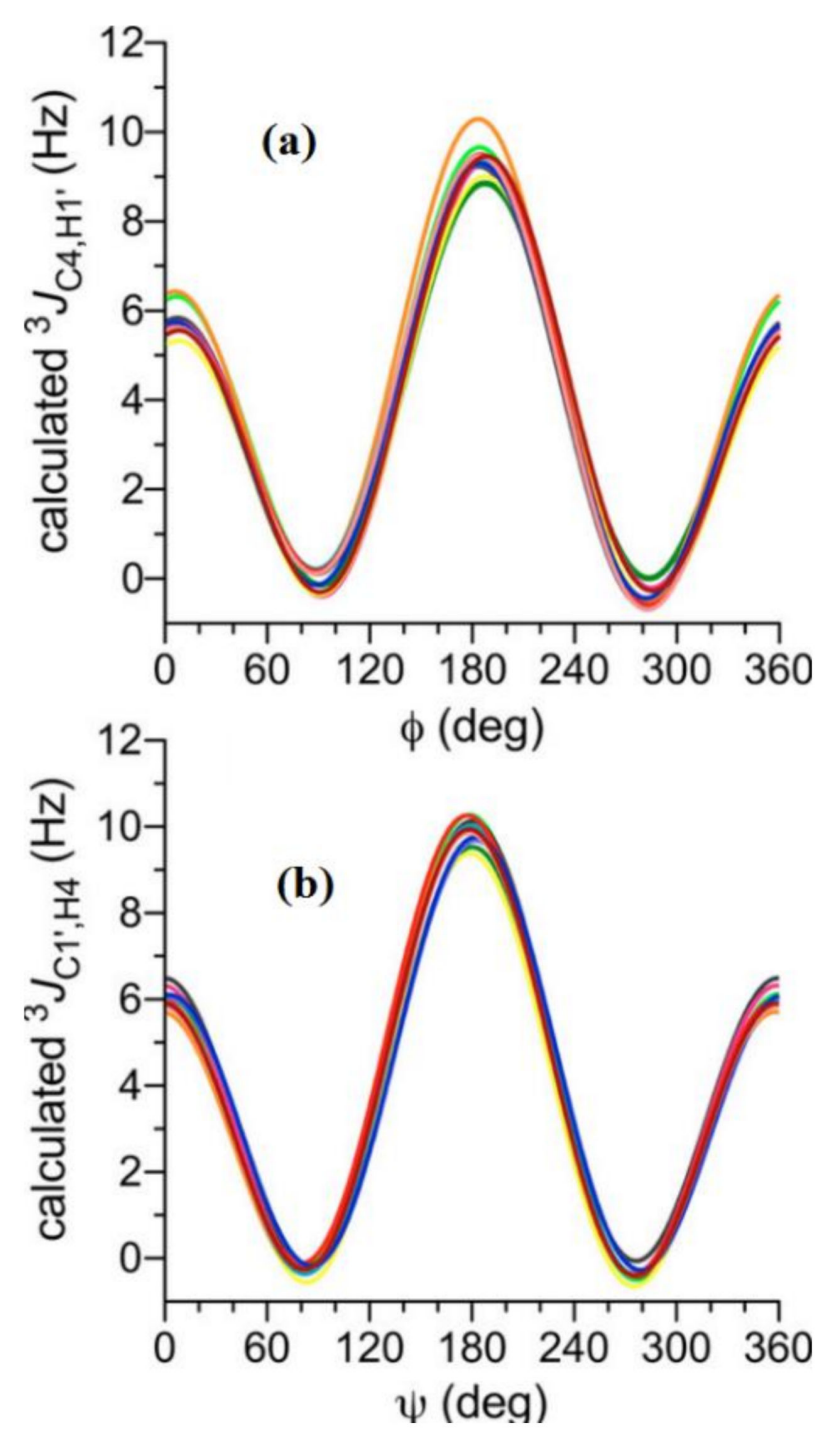
Calculated dihedral angle dependences of (**a**)—^3^*J*(C-4,H-1′) and (**b**)—^3^*J*(C-1,H-4′) in β-D-galactopyranosyl-(1→4)-β-D-glucopyranoside (**78**) as a function of φ and ψ. Reproduced from Zhang et al. [150] with the permission of the American Chemical Society.

**Figure 42:**
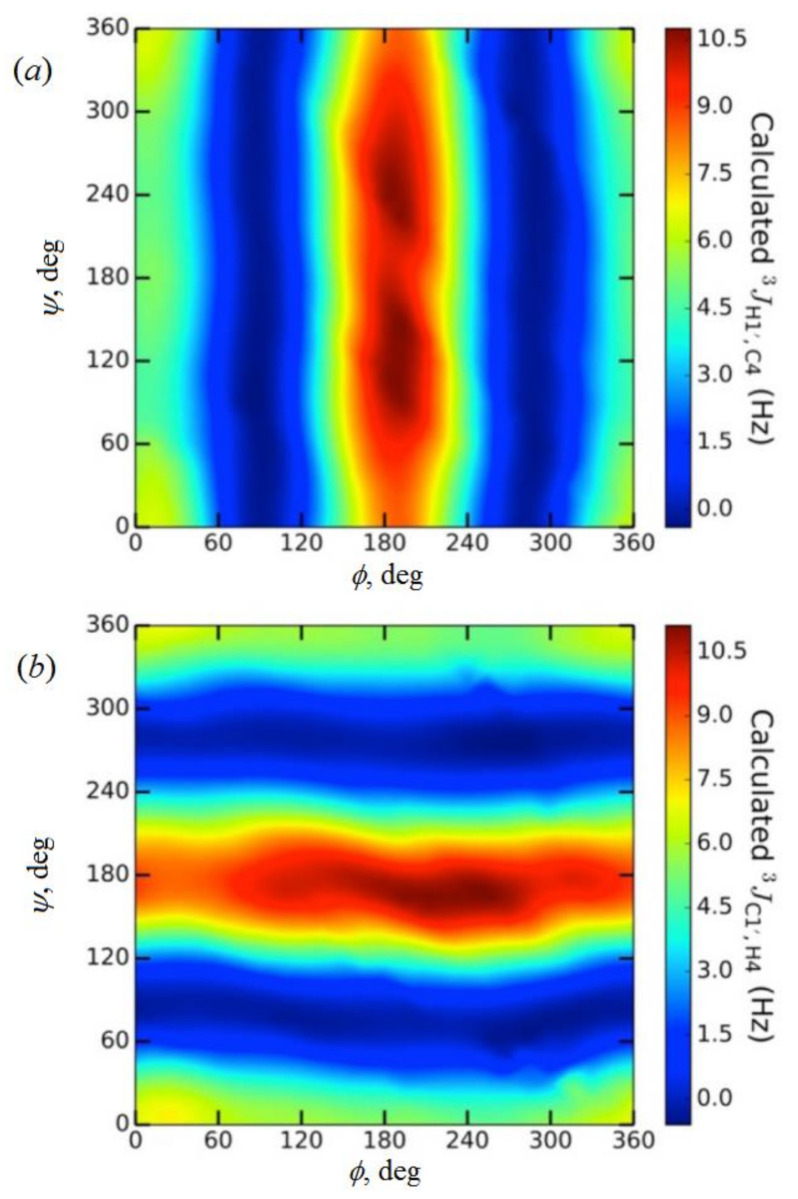
(**a**)—2D contour plot of calculated ^3^*J*(C-4,H-1′) values in β-D-galactopyranosyl-(1→4)-β-D-glucopyranoside (**78**), showing a primary dependency on φ; (**b**)—Contour plot of calculated ^3^*J*(C-1′,H-4) values, showing a primary dependency on ψ. Reproduced from Zhang et al. [150] with the permission of the American Chemical Society.

As illustrated in Figure 41, both trans-*O*-glycosidic ^3^*J*_COCH_ couplings showed primary dependencies on φ and exhibited dynamic ranges of about 10 Hz each. Visualizations of the DFT data presented in 2D contour plots, as shown for compound **78** in Figure 42, demonstrated that ^3^*J*(C-4,H-1′) was largely unaffected by ψ while ^3^*J*(C-1′,H-4) was largely unaffected by φ. It also followed that φ was unaffected by the structural changes in the vicinity of the β-(1→4) linkage, whereas ψ was affected noticeably.

**Scheme 7:**
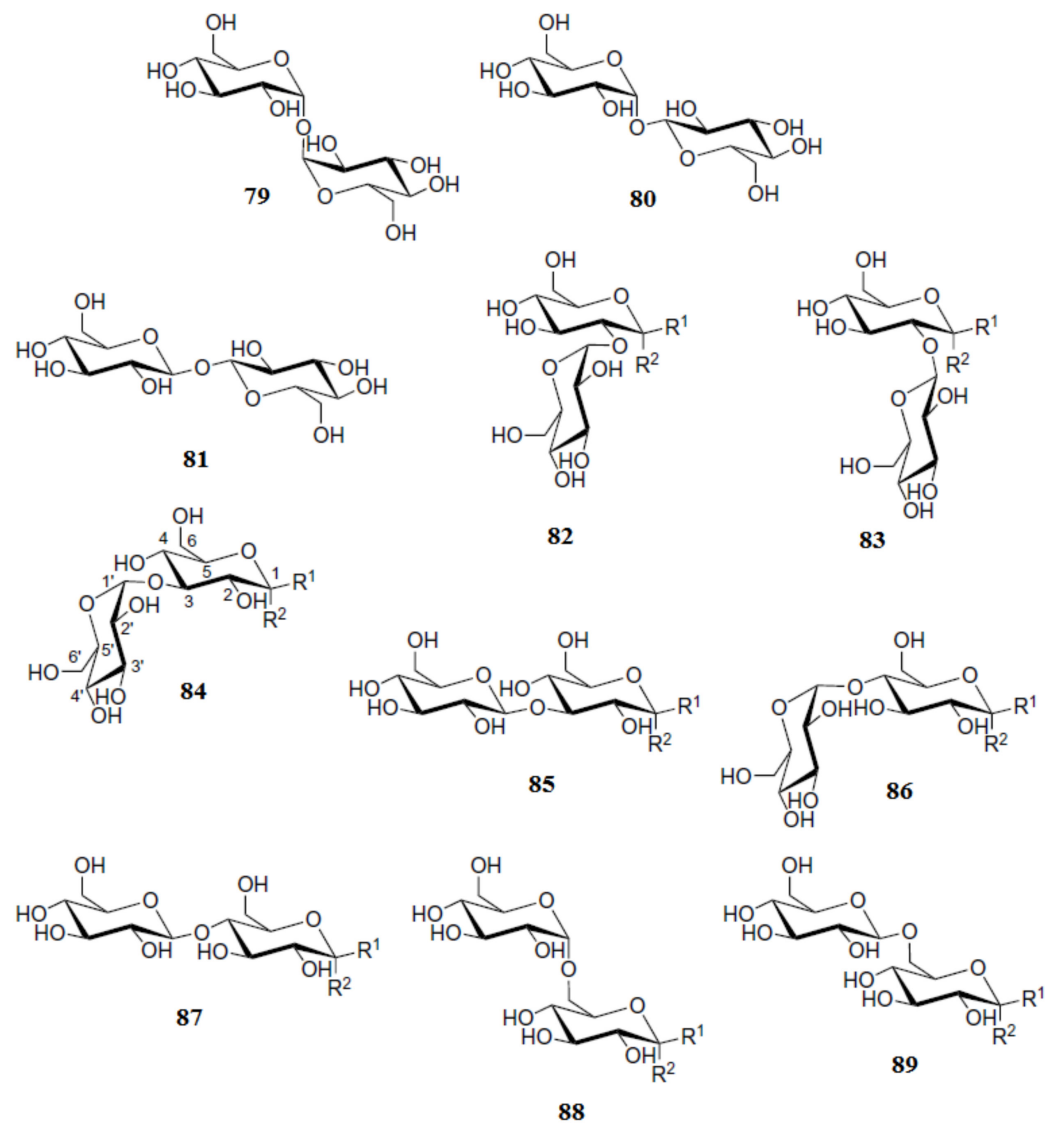
Chemical structures of the glucose-based disaccharides α,α-trehalose (**79**), α,β-trehalose (**80**), β,β-trehalose (**81**), kojibiose (**82**), sophorose (**83**), nigerose (**84**), laminaribiose (**85**), maltose (**86**), cellobiose (**87**), isomaltose (**88**), and gentiobiose (**89**). R^1^ and R^2^ stand for H and OH (or either OH and H) in different anomers. Reproduced with minor editing privilege from Roslund et al. [111] with the permission of Elsevier.

Roslund et al. [111] performed a combined computational and experimental study of proton-proton coupling constants in α and β anomers of the glucose-based disaccharides **79**–**89** shown in a Scheme 7. In that study, a B3LYP functional was used in combination with Jensen’s triple zeta pcJseg-2 basis set, specially designed for the calculation of nuclear spin-spin coupling constants. The effects of different glycosidic linkage types and positions on the glucose ring conformations and on the α/β-ratio of the reducing end hydroxyl groups were investigated. As a result of that study, rather small differences in ^3^*J*(H-4,H-5) coupling constants of the studied disaccharides were observed in the reducing unit of their different anomers. In general, the value of ^3^*J*(H-4,H-5) for the α-anomer was found to be more than 10 Hz while for the β-anomer it was less than 10 Hz. Generally, a reasonably good agreement of the performed calculations with experiment was achieved.

Coming to the discussion of papers dealing with the calculation of ^1^H and ^13^C-NMR isotropic chemical shifts and chemical shift tensors measured in solid state of di- and polysaccharides, we will first focus on the methodological survey by Sefzik et al. [151]. The authors of that paper studied the performance of the HF and DFT methods for this purpose. The quality of the scrutinized methods was assessed by their least-squares linear regression parameters. It was observed that in general DFT outperformed restricted HF theory. For instance, Becke’s functional B3 together with mPW1PW91 provided the best-predicted ^13^C-NMR shieldings. However, this performance was not universal, as none of the DFT functionals could generally predict saccharide tensors better than the HF theory.

Presented in Table 6 are the results of the least-squares correlation of calculated ^13^C-NMR chemical shift tensors for 65 saccharides evaluated using different functionals and basis sets of double and triple zeta quality versus experiment. It is seen that the overall quality of the established linear correlations is very impressive. Thus the RMSD of those correlations were found to be typically 4–5 ppm for the range of about 220 ppm (which is about 2% in relative terms) while the slopes were about (1.0–1.2), which is very close to unit in absolute scale. Interesting to note, the ongoing from the double zeta cc-pVDZ to the triple zeta cc-pVTZ basis set did not improve in general the quality of the correlations established in that paper [151], as can be seen in the values of RMSD given in the table.

It is noteworthy that results reached by Sefzik [152] and at the same period of time by Sergeyev and Moyna [152] were critically remarked by one of the reviewers of the presented review as follows: “I would strongly recommend caution and at least a critical remark when including this papers like this in a review. NMR chemical shifts require basis sets with tight exponents and DFT also has different basis set requirements than post HF methods, for which the Dunning basis sets applied by Sefzik have been developed. The fact that HF exhibits the lowest RMSD for the ^13^C-NMR shifts of 65 saccharides is in stark contrast to practically all relevant benchmark studies on chemical shifts and require a detailed conceptual discussion of all sources or error and an conclusion of how error cancellation leads to these results. The same applies to the discussion of Sergeyev and Moyna’s work (which is discussed next—LBK) using HF and 3-21G and 6-311G(d,p) basis sets”. In my opinion, this criticism is much acquitted.

**Scheme 8:**
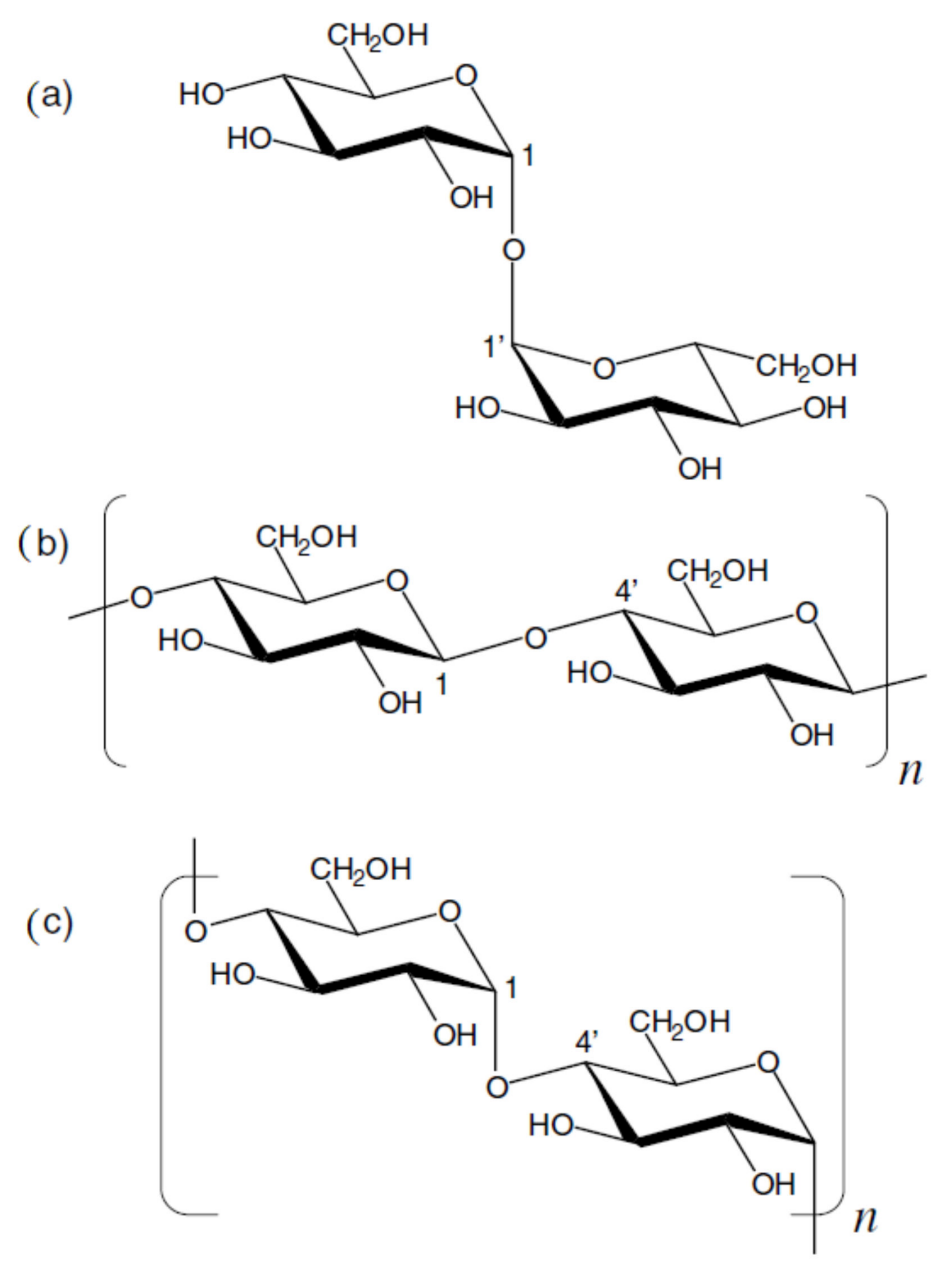
Chemical structures of trehalose (**a**), cellobiose (**b**, *n* = 1), cellotetraose (**b**, *n* = 2), cellohexaose (**b**, *n* = 3), cellulose (**b**, *n* > 100), and amylose (**c**, *n* > 100). Reproduced from Sergeyev and Moyna [152] with the permission of Elsevier.

**Figure 43:**
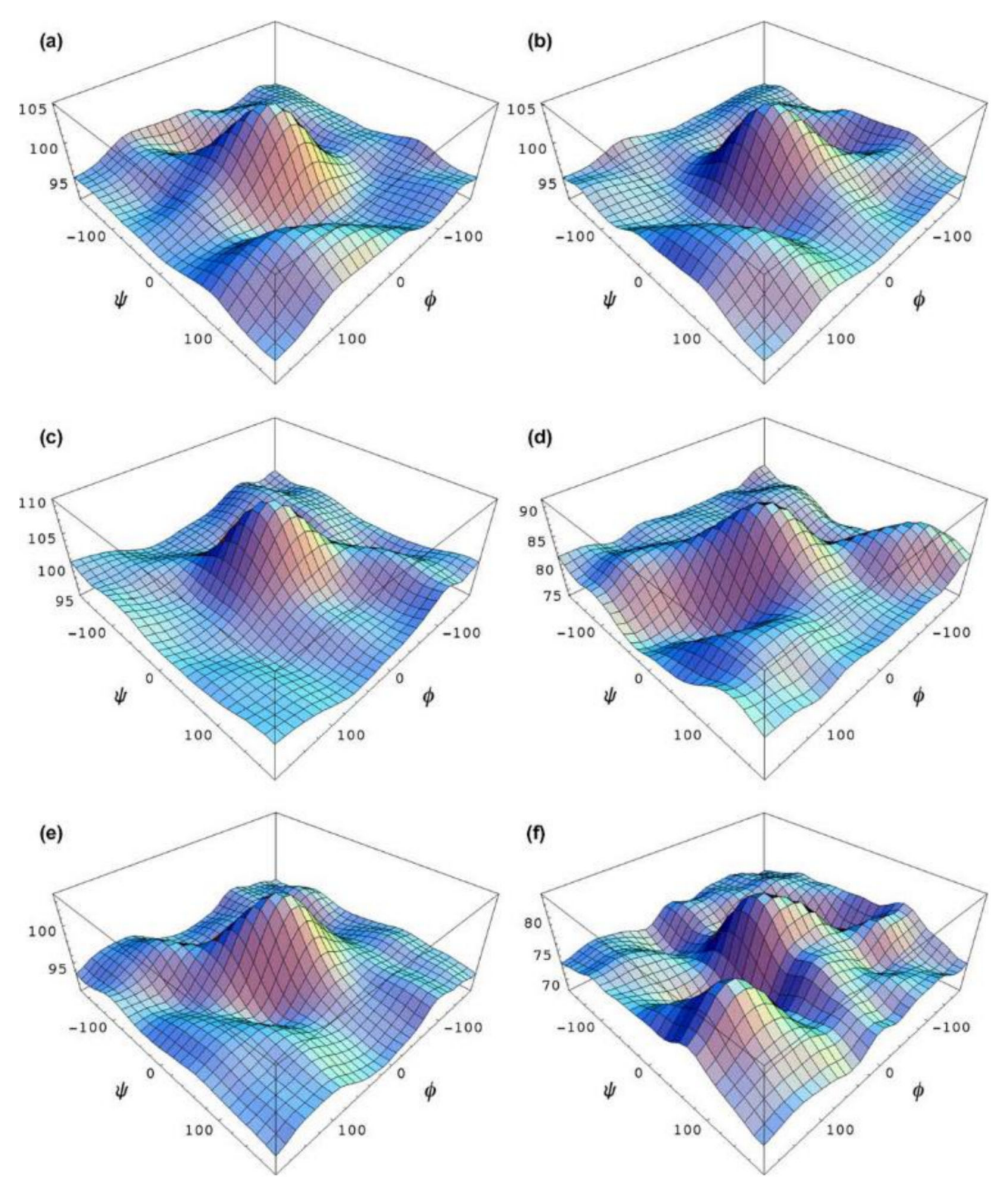
^13^C-NMR chemical shift surfaces for the pairs of transglycosidic carbons in α-(1→1′), β-(1→4′), and α-(1→4′)-linked D-Glcp disaccharides as a function of the glycosidic bond dihedrals. Corresponding surfaces are marked accordingly as (**a**–**f**). Reproduced from Sergeyev and Moyna [153] with the permission of Elsevier.

Sergeyev and Moyna [152] suggested a novel method for the determination of three-dimensional structure of oligosaccharides exemplified with trehalose, cellobiose, cellotetraose, cellohexaose, cellulose II, and amylose V_6_ in solid state. The structures of the examined oligosaccharides are shown in Scheme 8, while ^13^C-NMR chemical shift surfaces for the pairs of transglycosidic carbons in α-(1→1′), β-(1→4′), and α-(1→4′)-linked D-Glcp disaccharides as a function of the glycosidic bond dihedrals are presented in Figure 43. Calculated dihedral angles and ^13^C-NMR chemical shifts as compared to their equilibrium experimental values are given in Table 7. Calculations of ^13^C-NMR chemical shieldings performed in that study were conducted at the HF/3-21G level. The results obtained for the glycosidic carbons were converted into chemical shifts by subtracting the chemical shieldings computed at the same level of theory for the methyl carbons of a standard—tetramethylsilane, and scaled to the results of the reference HF/6-311G(d,p) calculations. It is seen that generally a very good agreement between theory and experiment has been achieved in that study.

It should be noted that very promising in the structural studies of carbohydrates is the application of molecular dynamics in combination with computational NMR, as exemplified in the paper by Kapla et al. [153] dealing with the molecular dynamics simulations and NMR spectroscopical studies of trehalose. The authors investigated interactions between lipid membranes and trehalose using NMR spectroscopy and molecular dynamics computer simulations. The dipolar coupling constants derived from theoretical trajectories were compared with those determined experimentally in a weakly ordered solvent and by the solid-state NMR. Analysis of the experimental and MD-derived couplings indicated that the force field used in the simulations reasonably well described experimental results. The signs of dipolar couplings, not available from the experiments on highly ordered systems, were determined from the trajectory analysis. The crucial step in the analysis of residual dipolar couplings was accessed to the conformational distributions derived from the MD trajectories. Furthermore, the conformational behavior of trehalose was investigated in the ordered and isotropic phases and was found to be essentially identical in both cases.

Later, the dissolution mechanism of cellulose in ionic liquids has been investigated [154] by the variable-temperature NMR using cellobiose and 1-ethyl-3-methylimidazolium acetate (EmimAc) as a model system. It was found that in DMSO-d_6_ solution, the hydrogen bonding was formed between the hydroxyl groups of cellobiose and both anion and cation of EmimAc. Based on that fact, it was concluded that both anion and cation had similar roles in the mechanism of cellulose dissolution. However, it was claimed in a forthcoming critical comment [154] that both experimental and theoretical studies published by different authors indicated that imidazolium ion was not involved in the H-bonding with a sugar solute, as was proposed in the former study.

**Figure 44:**
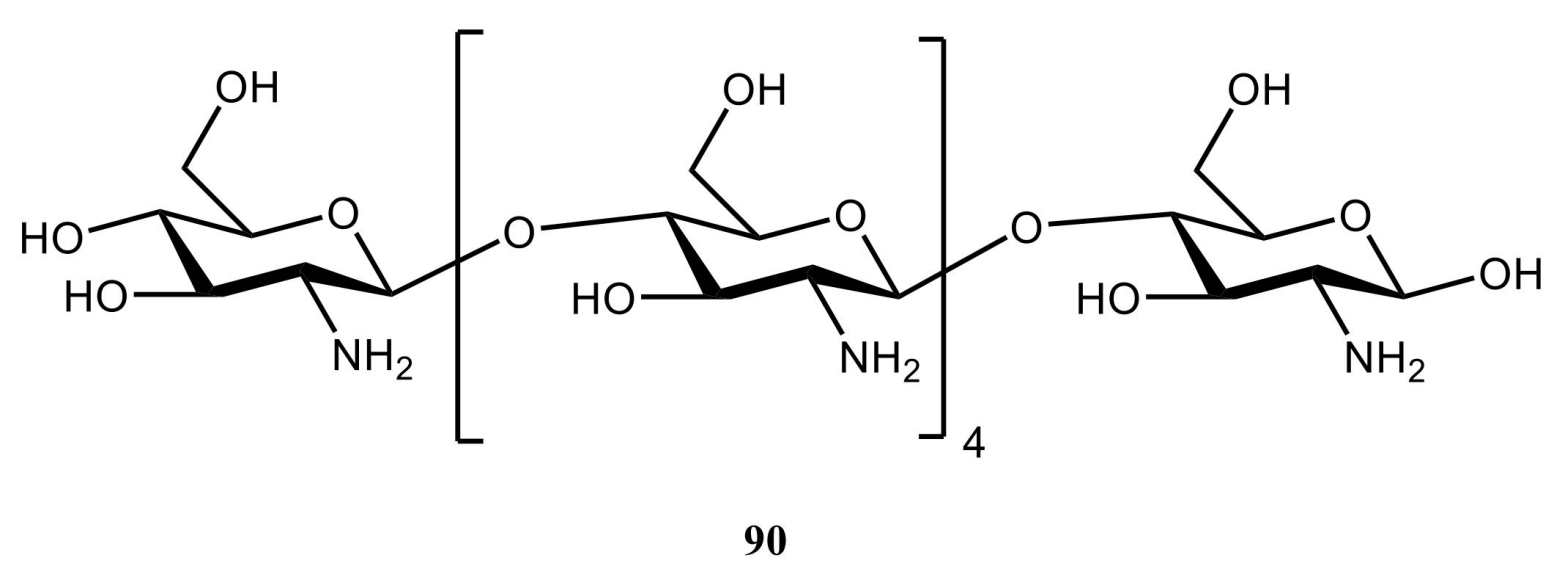
Chemical structure of hexameric chitozan.

**Figure 45:**
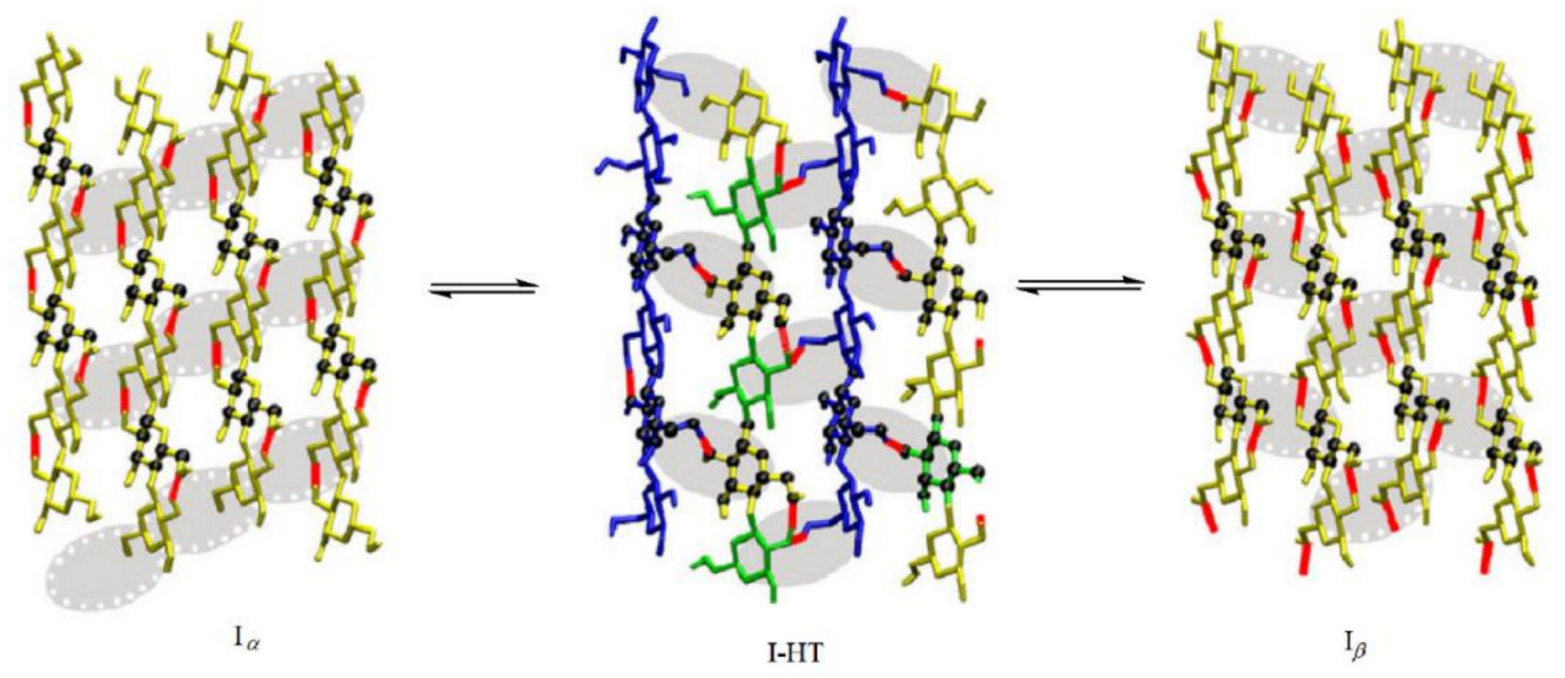
Interconversion of cellulose crystalline forms I_α_ and I_β_ via a high temperature intermediate I-HT. For details, see cited paper. Reproduced with minor editing privilege from Matthews et al. [156] with the permission of Springer Nature.

Esrafili and coauthors [155] performed DFT calculations of ^1^H, ^13^C, ^15^N, and ^17^O chemical shielding tensors in the anhydrous crystalline structure of hexameric chitozan (**90**) (Figure 44). The hydrogen-bonding effects in cellulose crystalline forms I_α_ and I_β_ interconverting to each other via a high temperature intermediate I-HT (see Figure 45 taken from the paper [156]) were taken into account in that study. The authors of the cited publication [155] included the most probable interacting molecules with a target molecule in a crystalline phase, the latter considered as a hexameric cluster. In this model, central molecule was surrounded with five other molecules, which participated in the intra- and intermolecular hydrogen bonding with central moiety. The general finding of the performed study [155] was that intermolecular hydrogen bonding had a major influence on the NMR parameters in isotropic and crystalline states. In addition, the quantum chemical calculations performed with the B3LYP functional and Pople’s 6-311++G(d,p) and 6-31++G(d,p) basis sets indicated that intra- and intermolecular hydrogen bonding played an essential role in the determining the chemical shielding principal components in the molecular frame axes.

**Figure 46:**
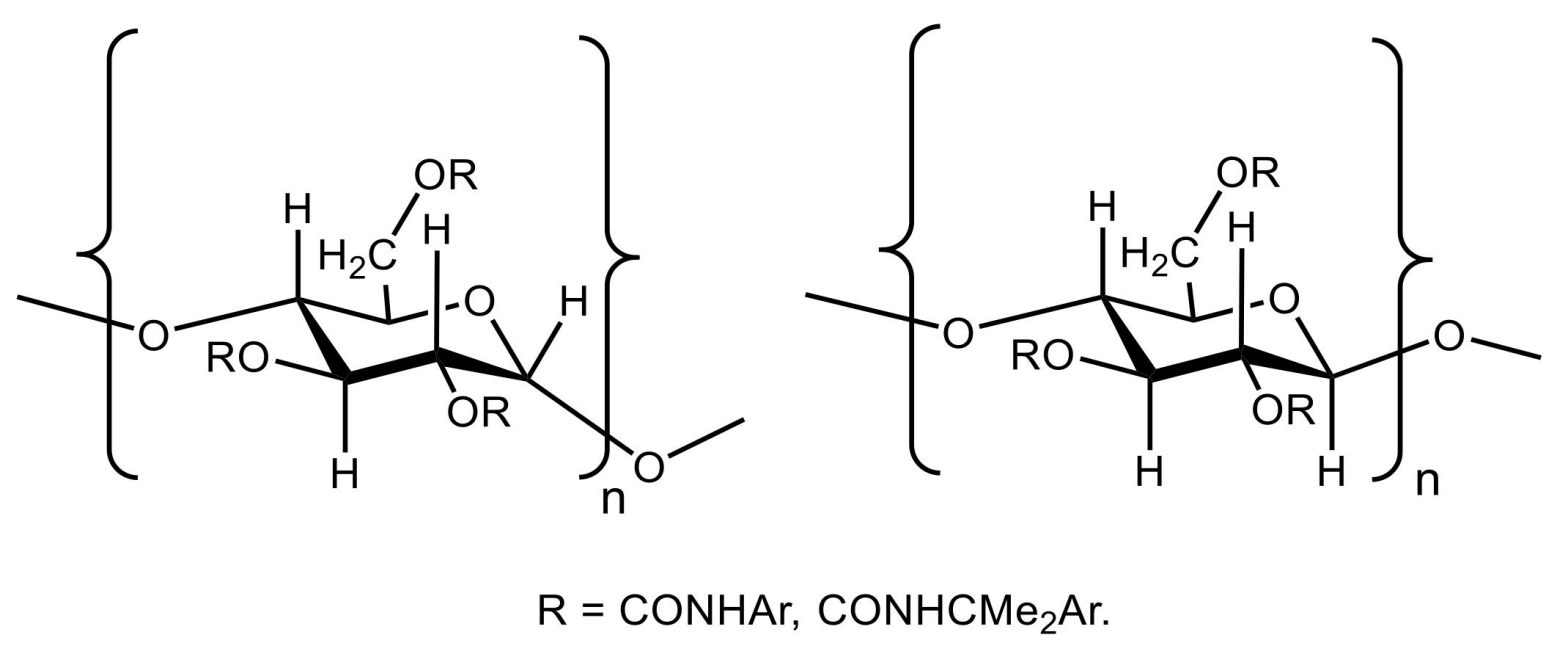
Chemical structures of amylose tris(3,5-dimethylphenylcarbamate) (ADMPC), amylose tris[(S)-R-methylbenzylcarbamate] (ASMBC), and cellulose tris(3,5-dimethylphenylcarbamate) (CDMPC). Amylose has the α-D-(1→4) linkages and cellulose has the β-D-(1→4) linkages.

Kasat et al. [157] studied the effects of the backbone and side chain on the molecular environments in the chiral cavities of three commercially important polysaccharide-based chiral sorbents. They were cellulose tris(3,5-dimethylphenylcarbamate) (known as “CDMPC”), amylose tris(3,5-dimethylphenylcarbamate) (known as “ADMPC”), and amylose tris[(*S*)-R-methylbenzylcarbamate] (known as “ASMBC”) with the general formulas of α and β anomers shown in Figure 46. Those studies were performed using the ^13^C Cross-Polarization/Magic-Angle Spinning (CP/MAS) and Magic-Angle Spinning (MAS) solid-state NMR together with intensive DFT calculations.

All NMR chemical shifts for the polymer backbone monomers and dimers were predicted at the B3LYP/6-311+G(d,p) level. It was concluded that molecular environments of the C=O, NH, and phenyl groups showed significant differences in the intramolecular and intermolecular interactions and in the nanostructures of the chiral cavities of those biopolymers. In general, reported results [157] demonstrated how molecular environments of the chiral cavities did affected their molecular recognition mechanisms, which could be established by the relevent DFT calculations in combination with the solid-state NMR experiment.

**Figure 47:**
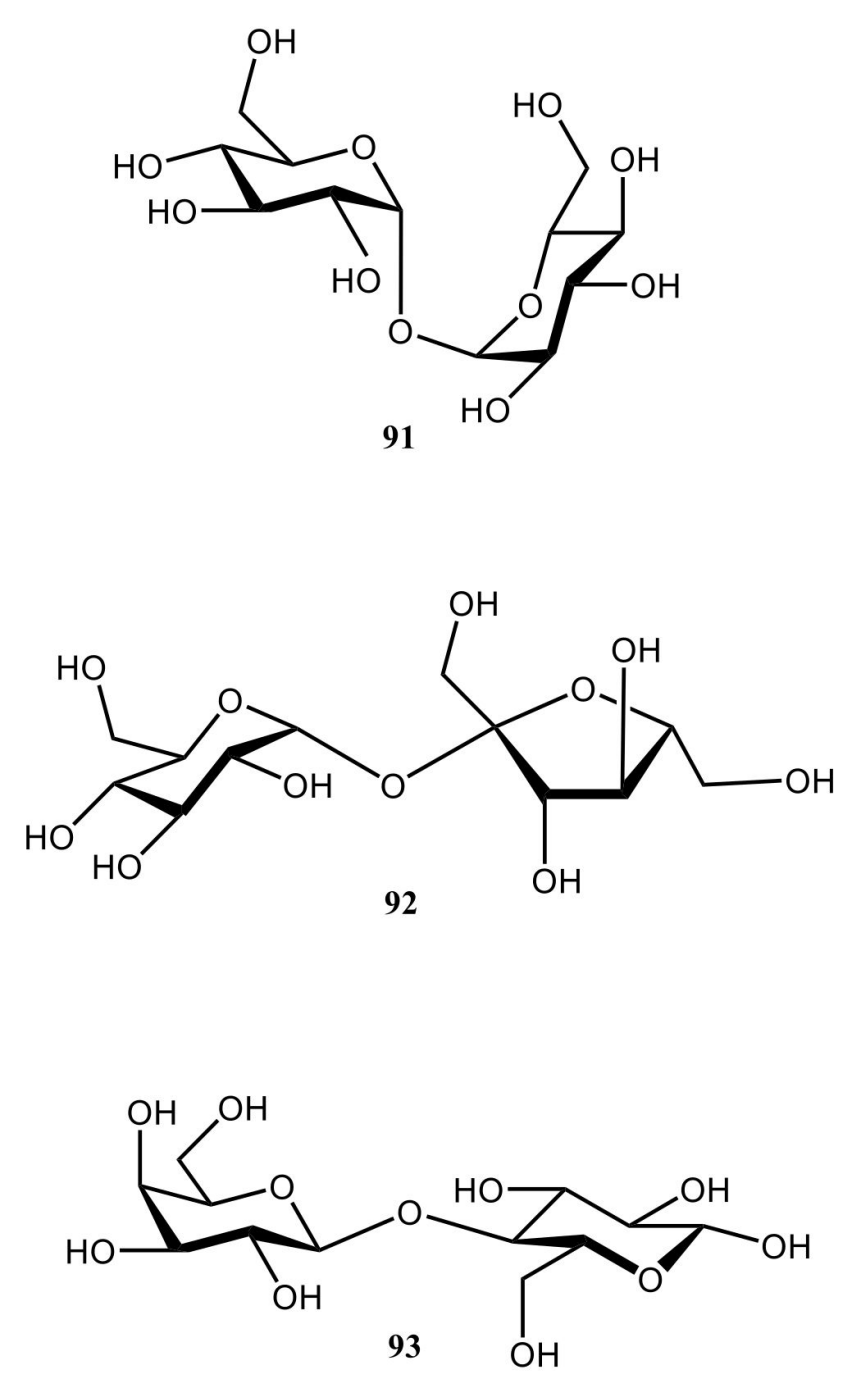
Chemical structures of three classical disaccharides, trehalose (**91**), sucrose (**92**), and lactose (**93**).

Lefort and coauthors [158] demonstrated that chemical shift surfaces contained information on distributions of various conformations of disaccharides in glassy solid state. The authors found with paying particular attention to the glycosidic linkage atoms in three classical disaccharides, trehalose (**91**), sucrose (**92**), and lactose (**93**) (see Figure 47), that chemical shift surfaces of glassy disaccharides were almost independent of the method chosen for optimizing their molecular conformations.

In particular, a proper consideration of extremes and saddle points of the chemical shift map was correctly accounted for the observed discontinuities in the experimental cross polarization magic angle spinning spectra. It was demonstrated that if those basic requirements were met, the Gauge Including Atomic Orbitals (GIAO) calculated on relaxed conformations lead to a good description of the experimental line shapes. In this approximation, London orbitals include the gauge origin in an exponential pre-factor, removing gauge-origin dependences for some of the integrals and to lowest order. However, it can be rigorously shown that those results are not strictly fully gauge independent.

The results of the performed study [158] were interpreted in terms of an enhanced flexibility that disaccharides possess in the amorphous solid state, especially on the ^13^C-NMR time scale. Using amorphous trehalose as a model, the authors considered its most populated conformations in glass state. It was also suggested that ab initio methods were not always necessary for defining the conformational potential basin. Indeed, performed calculations based on the combination of molecular mechanics (MM), semiempirical Austin Model 1 (AM1), and DFT (B3LYP and B3PW91 functionals) with Pople’s basis sets, 3-21+G(d,p) and 6-311+G(d,p), provided the mostly convincing results.

**Figure 48:**
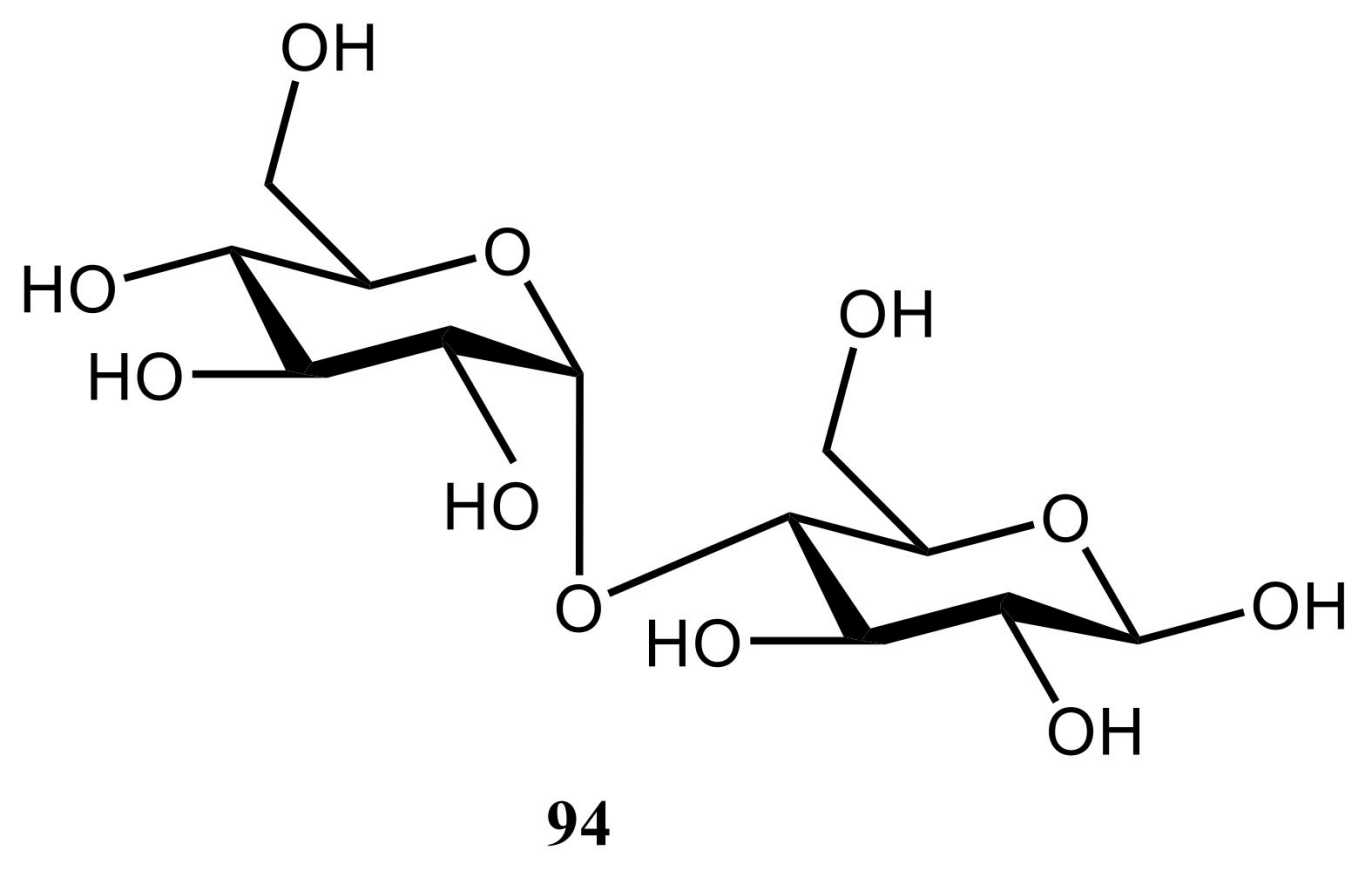
Chemical structure of maltose. For the structures of trehalose and sucrose, see Figure 46.

In addition to that study [158], Shao and coworkers [159] investigated the ability of DFT with using the PBE exchange-correlation functional to reproduce experimental principal components for trehalose (**91**), sucrose (**92**), and maltose (**94**), the latter shown in Figure 48, in solid state and in isotropic phase. A method for assigning poorly dispersed NMR spectra based on comparing experimental and calculated shift anisotropies within the planewave-pseudopotential approach [160] as well as isotropic shifts has also been described.

**Scheme 9:**
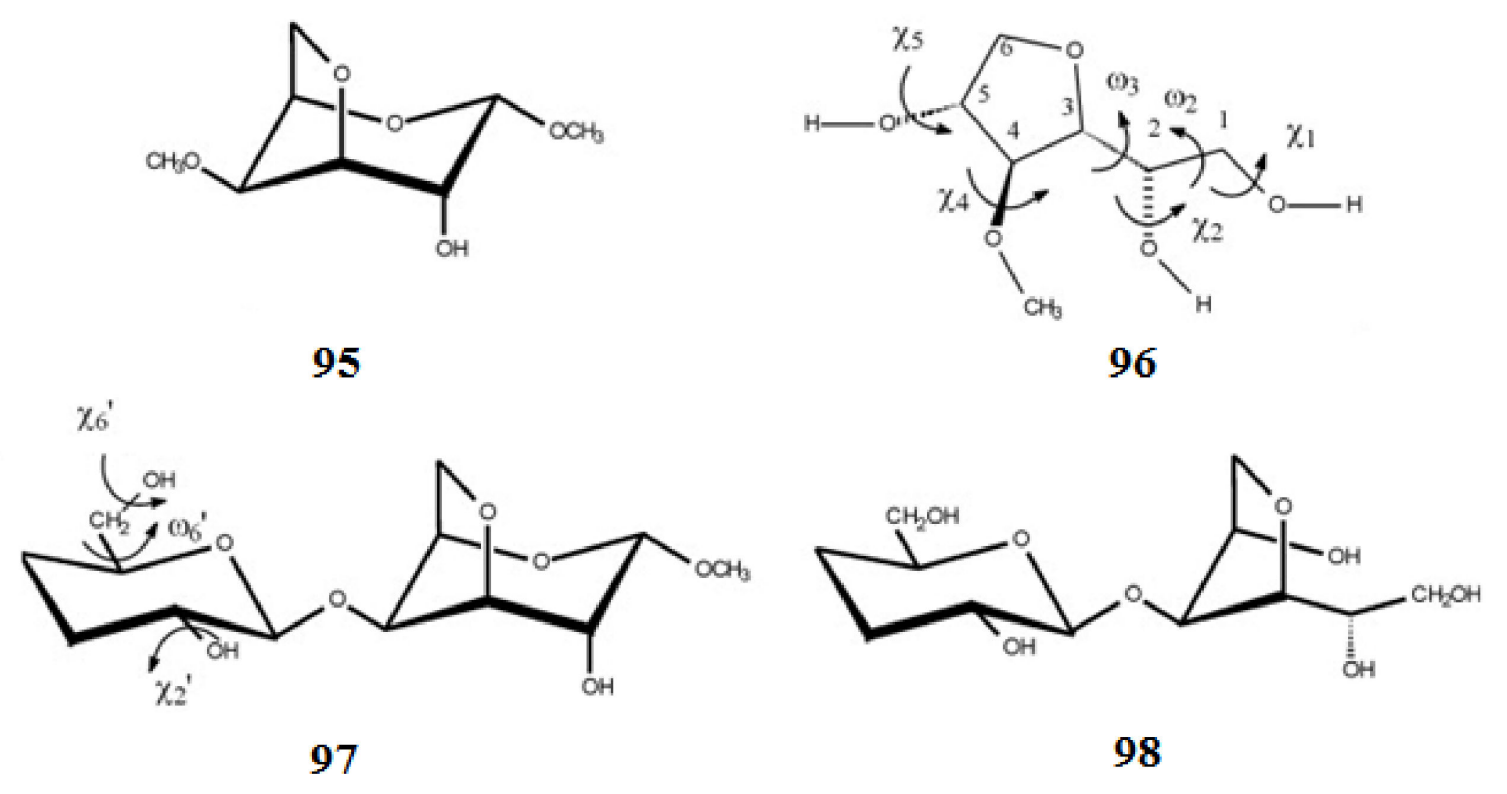
Chemical structures of methyl 3,6-anhydro-4-*O*-methyl-α-D-galactoside, 3,6-anhydro-4-*O*-methylgalactitol, and 3′,4′-dideoxy derivatives of the α-methyl glycoside of carrabiose and carrabiitol. Reproduced from Navarro and Stortz [161] under an open access Creative Commons Attribution 4.0 International Public License.

Different conformations of methyl 3,6-anhydro-4-*O*-methyl-a-D-galactoside (**95**) and 3,6-anhydro-4-*O*-methylgalactitol (**96**) together with 3′,4′-dideoxy derivatives of the α-methyl glycoside of carrabiose (**97**) and carrabiitol (**98**) are shown in Scheme 9. Those structures were reached at by the molecular mechanics and by quantum mechanical methods at the DFT level of theory using B3LYP functional with 6-31+G(d,p) basis set and at the MP2 level with 6-311++G(d,p) basis set with and without taking into account solvent effects [161]. In particular, for compound **96**, where the five-membered ring was free to move, two main stable conformations were found. Calculation of ^1^H and ^13^C-NMR chemical shifts was performed at the same level of theory.

Suzuki et al. [162] studied the effects of conformation and hydrogen bonding on the ^13^C isotropic chemical shifts of the cellobiose units of the native cellulose (**99**) at the DFT level. The linear relationship between the chemical shifts of C^4^, C^5^, and C^6^ carbons and the torsion angles around the C−O bonds in the corresponding CH_2_OH side groups was found. It is well known that the cellobiose unit (the smallest repeating unit in the polymer) consists of two anhydroglucose units joined through the single-oxygen atoms (acetal linkages) between the C^1^ of one pyranose ring and the C^4^ carbon of the next ring. This is known as the β-1,4-linkage (for the native cellulose structure, see one of the most comprehensive reviews [163] and references given therein). Both geometry optimizations and computation of ^13^C-NMR chemical shifts in that study were carried out at the B3LYP/6-31+G(2d,p) level.

Performed calculations for the cellobiose units in native cellulose revealed the *γ*_H_-gauche effect produced by the OH hydrogen atom at the γ position to induce a 3–5 ppm downfield ^13^C-NMR shift, and this effect was reduced by 2–3 ppm when the intramolecular hydrogen bonding associated with the γ-H atom was formed. The appreciable dependences of the C^1^ and C^4^ chemical shifts on the torsion angles around the β-1,4-glycosidic linkage were also reported in that paper [162].

**Scheme 10:**
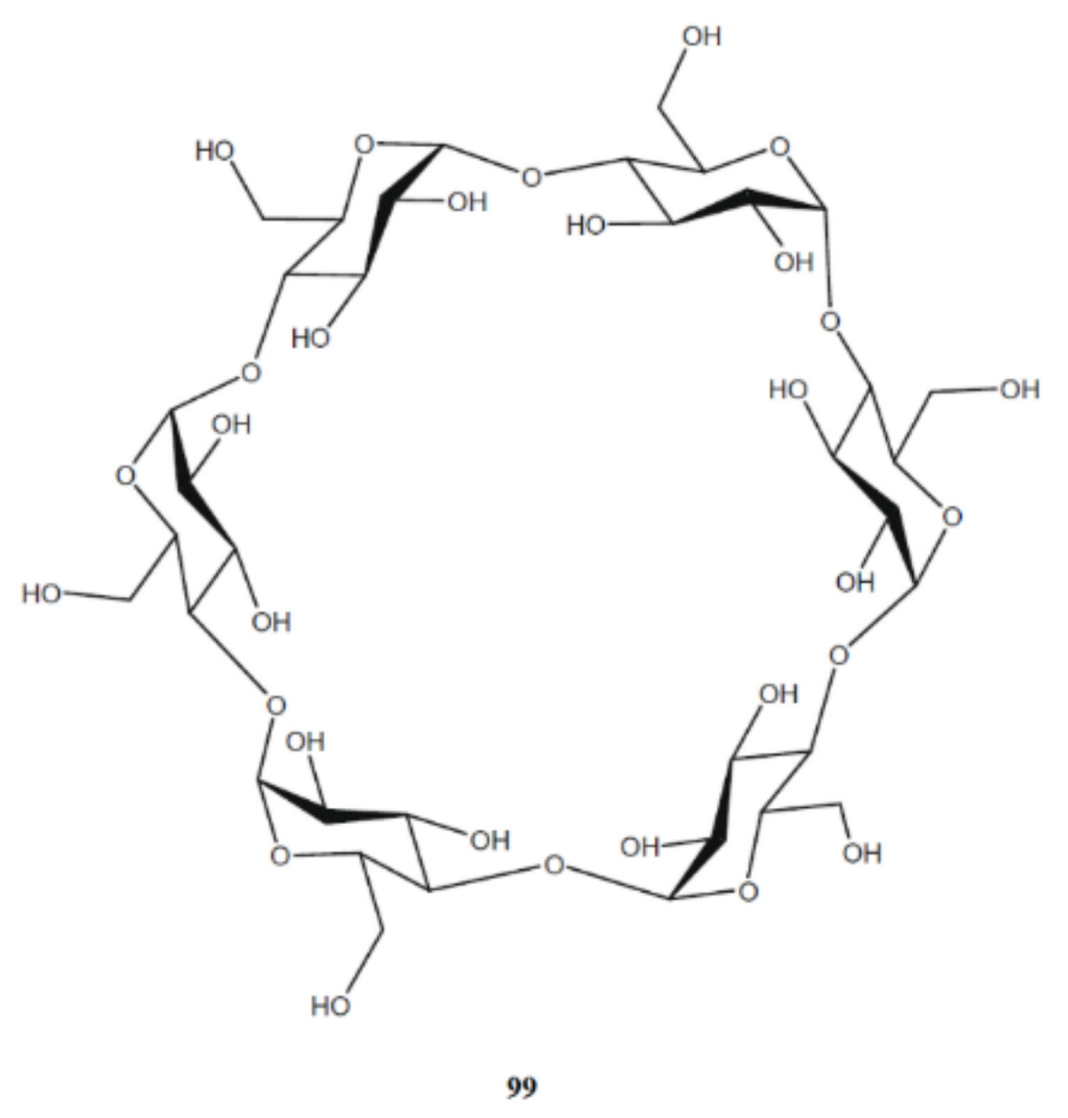
The structure of α-cyclodextrin (six subunits). Reproduced from Tafazzoli and Ghiasi [164] with the permission of Elsevier.

Tafazzoli and Ghiasi [164] performed a systematic study of the dependence of the anomeric ^13^C-NMR chemical shift on the glycosidic bond dihedral angles in D-Glcp-D-Glcp disaccharides with the (1→4) linkages in the α-configurations of α-, β-, and γ-cyclodextrins. Those molecules are exemplified here in Scheme 10 with the structure of α-cyclodextrin shown below possessing six subunits. Cyclodextrins are the cyclic oligosaccharides built up from the α-D-glucopyranose units connected through the α-1,4 glycosidic linkage. The cyclodextrin molecule includes six glucopyranose units, while β- and γ-cyclodextrins consists of accordingly, seven and eight units, arranged in a ring-shape manner. Cyclodextrins have ability to form hydrogen bonds with the surrounding molecules resulting in the formation of a number of inclusion complexes with a variety of inorganic and organic substances.

**Figure 49:**
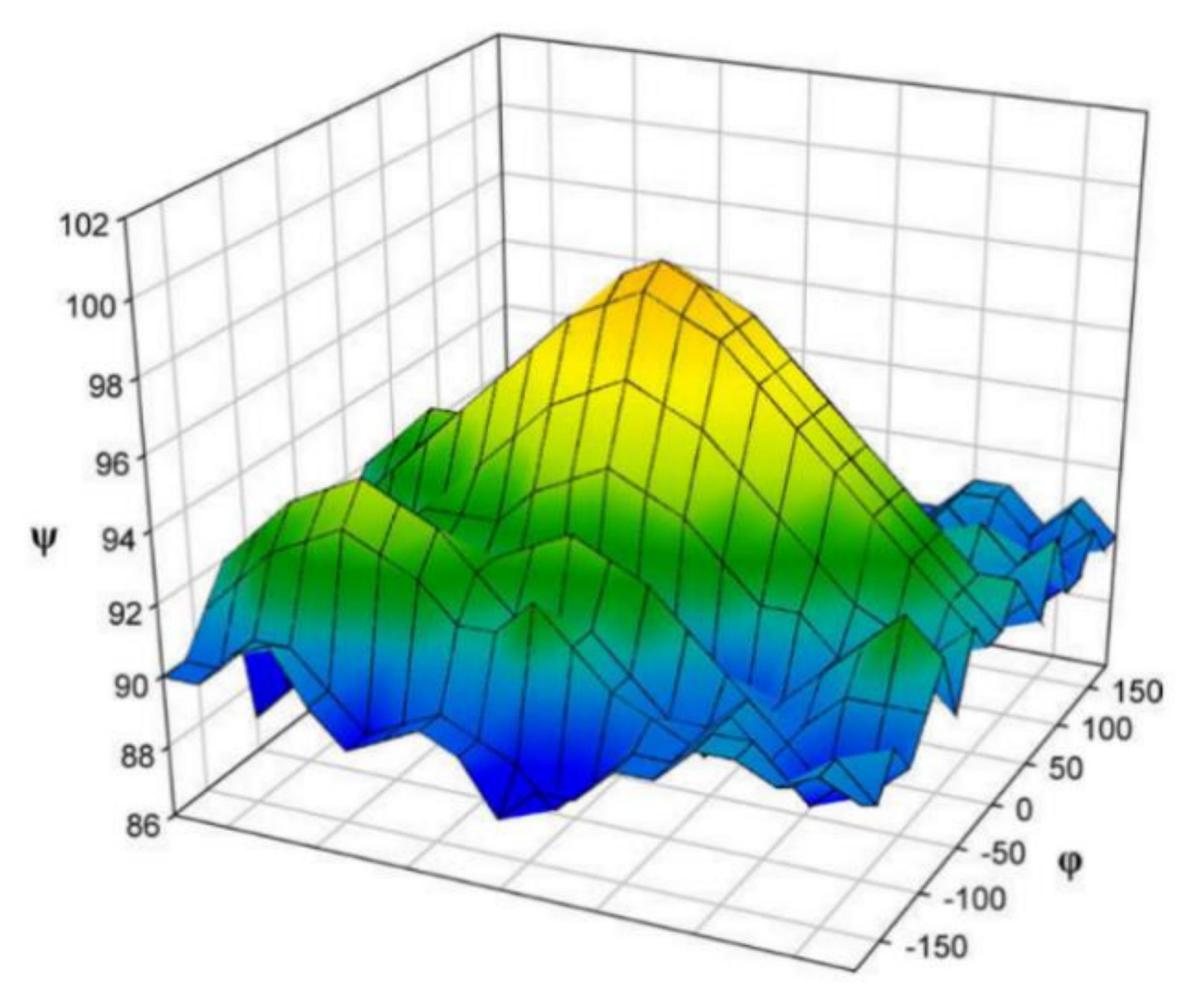
The ^13^C-NMR chemical shift surfaces of the anomeric carbon of D-Glcp-α-(1→4)-D-Glcp disaccharide. Reproduced from Tafazzoli and Ghiasi [164] with the permission of Elsevier.

In the course of that study [164], the chemical shift surfaces versus the dihedral angles φ and ψ for the D-Glcp-α-(1→4)-D-Glcp moiety were computed and plotted as shown in Figure 49. Based on those results, the empirical formulas in the form of δ(^13^C) = ƒ(φ,ψ) were obtained by fitting computational data to the trigonometric series expansions. Those results were consistent with the experimental observations demonstrating the applicability of the ^13^C-NMR chemical shift surfaces to the study of the conformational behavior of oligosaccharides. Both geometry optimizations and calculations of ^13^C-NMR chemical shifts were performed at the DFT (B3LYP) and HF levels using Pople’s 6-31G(d) basis set in the IEF-PCM medium (water).

**Figure 50:**
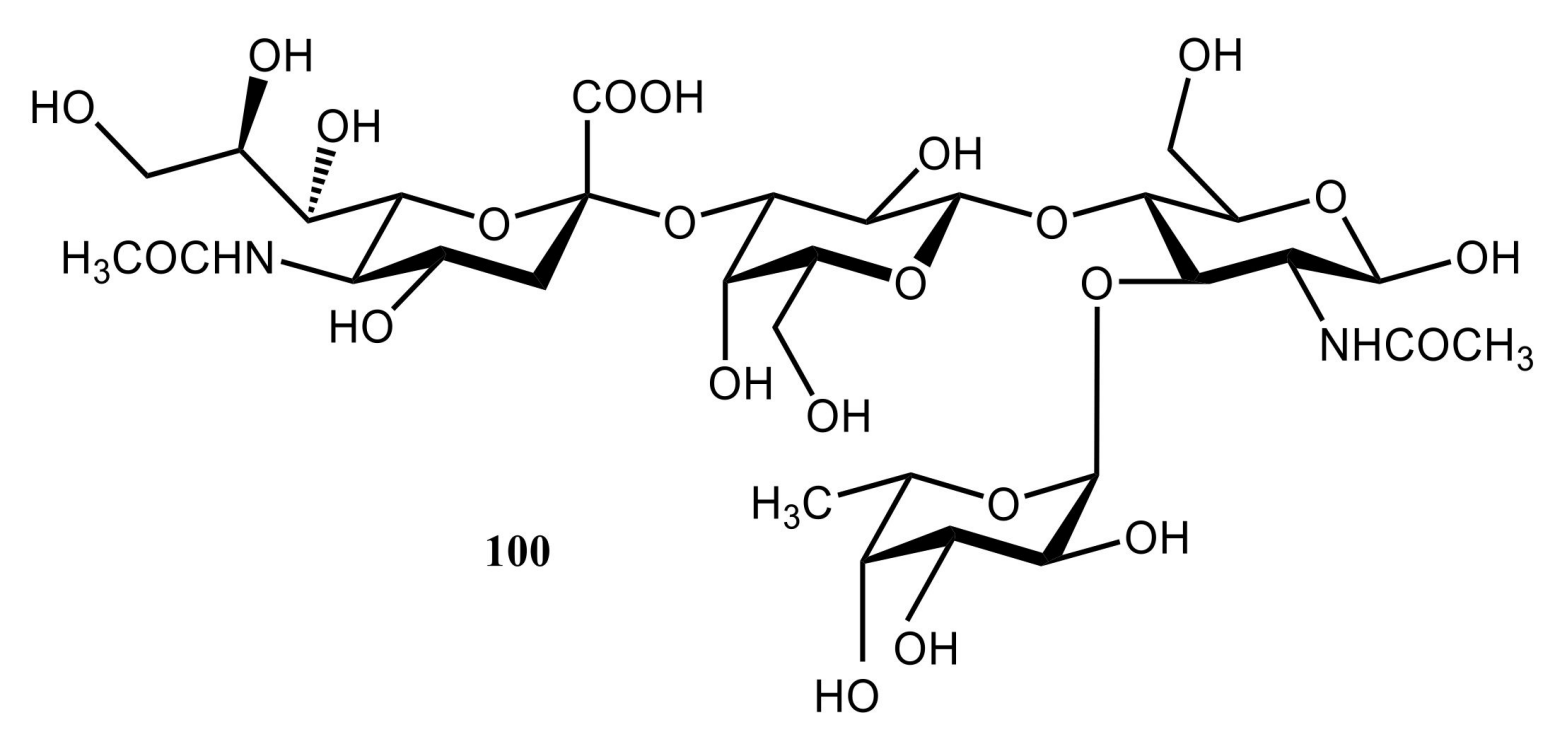
Model structure of the E-selectin complex consisting of four sugar components, which are *N*-acetylneuramic acid (Neu5Ac), galactose (Gal), *N*-acetylglucosime (GlcNAc), and fucose (Fuc).

Ishida [165] performed a computational modeling of the carbohydrate-recognition process in the E-selectin complex with sialyl Lewis X (known as the E-selectin/SLe^x^). That complex consists of four sugar components, which are *N*-acetylneuramic acid (Neu5Ac), galactose (Gal), *N*-acetylglucosime (GlcNAc), and fucose (Fuc) presented by structure **100** (Figure 50). This model clearly demonstrated that the binding geometries of the E-selectin/SLe^x^ complex were determined not by a single rigid carbohydrate structure, but rather by the sum of the averaged conformations fluctuating around the minimum free energy region. It was demonstrated that major molecular interactions in **100** were hydrogen bonds between the fucose moiety and the Ca^2+^ binding site in the carbohydrate recognition domain, and that it was galactose unit which was important in determining the ligand specificity.

**Figure 51:**
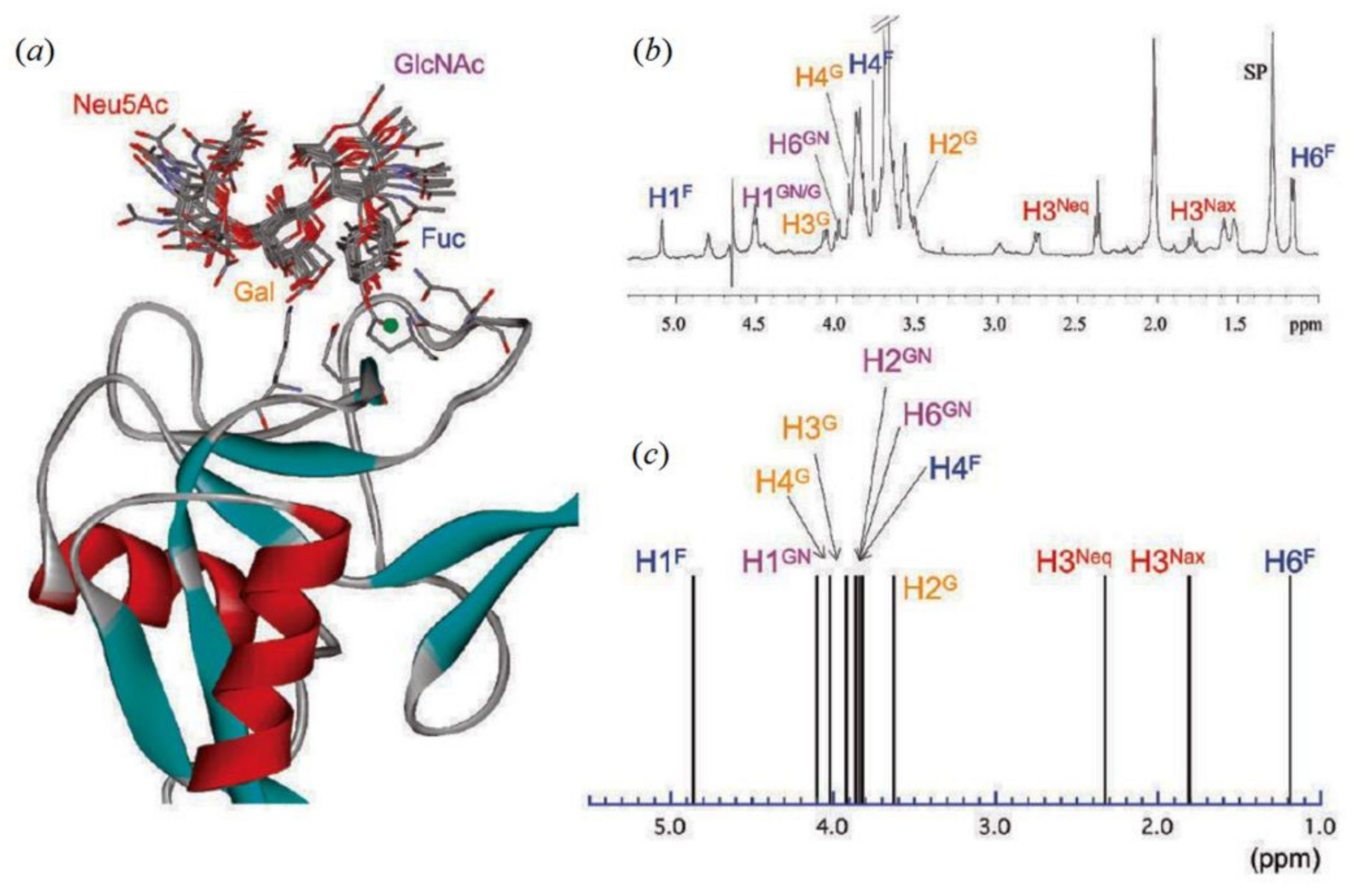
(**a**)—Superimposed geometries of a sialyl Lewis X complex at the carbohydrate-recognition domain. Ten QM/MM optimized structures of SLe^x^ are superimposed onto one reference structure. Only the SLe^x^ structures are shown by the stick model. Right panels: (**b**)—Experimental NMR profile. (**c**)—Computed 1D proton chemical shift profile of the SLe^x^ ligand. Reproduced from Ishida [165] with the permission of the American Chemical Society.

Calculations of ^13^C-NMR chemical shifts performed at the QM/MM/RHF/6-31G(d)/AMBER level in comparison with experiment revealed most favorable conformations of the E-selectin/SLe^x^ complex shown in Figure 51a. The deviations of calculated chemical shifts from their mean average values were found to be rather small, typically in the range of 0.2–0.4 ppm (as compared to the experimental accuracy of about 0.1 ppm). However, the binding conformation of SLe^x^ was clearly not rigid but essentially flexible, especially in the solvent-exposed region. It also followed that the Fuc unit formed stable hydrogen bonds with the Ca^2+^ binding site while the Gal moiety interacted mostly with the protein.

It was also demonstrated [165] that Fuc and Gal residues provided rather small structural fluctuations, while Neu5Ac and GlcNAc, on the contrary, showed much larger molecular motions. By averaging calculated chemical shifts for the QM/MM-refined geometries, the 1D-NMR theoretical spectral profile was obtained (Figure 51c). In comparing theoretical results with the available experimental spectrum (Figure 51b), it followed that calculated 1D-NMR profile qualitatively reproduced experimental results within the accuracy of the employed method, QM/MM-RHF/6-31G(d).

The major findings of the performed study [165] were formulated by the author as follows: “(1)—In the Neu5Ac unit, there are no apparent hydrogen bonds around the carboxyl group, and this unit is more flexible along the glycosidic linkage than are any of the other three sugar units. (2)—In the Gal unit, the 4-OH group is always located near the hydroxyl group of Tyr94, although no apparent hydrogen bonds are formed. The major molecular interactions are the hydrogen bonds between the 6-OH group and the side chains of Glu107 and Lys111. (3)—In the GlcNAc unit, there is no apparent close molecular contact between the hydroxyl groups of the sugar unit and the target amino acid residues. However, the side chain of the NHAc group is relatively flexible, and occasionally forms a hydrogen bond with the side chain of Arg108. (4)—In the Fuc unit, rather stable hydrogen bonds form because of the three types of hydroxyl groups. The 2-OH group can form a moderately strong hydrogen bond with the side chain of Asn83, the 3-OH group occasionally forms a hydrogen bond with Glu107, and the 4-OH group always forms tight and stable hydrogen bonds with Glu80 and Asn82”.

A number of more illustrative examples dealing with stereochemical applications of computational ^1^H and ^13^C chemical shifts together with ^1^H-^1^H, ^13^C-^1^H, and ^13^C-^13^C spin-spin coupling constants of carbohydrates can be found in a series of original papers reviewed elsewhere [1,2,7].

## 5. Conclusions

Nowadays, computational NMR is widely used for structural and stereochemical studies of a wide series of carbohydrates including mono-, di-, and polysaccharides together with their functional derivatives. Stereochemical analysis of carbohydrates could mostly be performed by using computational versus experimental ^1^H and ^13^C-NMR chemical shifts and spin-spin coupling constants in view of their marked stereochemical dependences providing a new guide in stereochemical structure of carbohydrates together with fundamental factors controlling molecular recognition and catalysis in biochemical systems.

Theoretical calculations of NMR parameters are currently performed at either non-empirical level with taking into account electronic correlation in an explicit way or within the DFT level including electronic correlation effects inexplicitly. Both levels are intensively exploited in the calculation of NMR chemical shifts and spin-spin coupling constants of simple inorganic, organic, bioorganic, and larger biological molecules involving in particular, natural products and carbohydrates.

Not surprisingly, calculations of NMR chemical shifts and spin-spin coupling constants in carbohydrates and, all the more so, in larger biological species are performed mostly within the DFT framework, in contrast to the non-empirical computations applied to much smaller molecules. This is not surprising in view of the fact that at the DFT level such calculations are much more economic, as compared to the non-empirical methods. However, it should be stressed that results of any DFT calculation drastically depend on the choice of a particular functional and, to a lesser extent, of a particular basis set, so that any DFT results are certainly to a much extent ambiguous.

In general, calculation of NMR parameters deals with a proper choice of the level of theory (either DFT or ab initio), application of the locally dense basis set schemes (if any), taking into account solvent effects, rovibrational corrections and relativistic effects (in the presence of heavy atoms).

At the non-empirical level, a number of correlated methods, like MP2, CCSD, CCSD(T), CC3, and CCSDT, are currently used for the calculation of chemical shifts showing very promising but enormously expensive results. For spin-spin coupling constants, Sauer’s suite of methods, SOPPA(MP2), SOPPA(CC2), and SOPPA(CCSD), are very budding in terms of the degree of electron correlation taken into account versus computational effort.

At the DFT level, among dozens and dozens of various local spin-density, exchange, correlation and exchange-correlation functionals used for the calculation of NMR chemical shifts and spin-spin coupling constants, the mostly popular are the following ones: B3LYP, BHLYP, OLYP, OPBE, OPW91, PBE0, KT2, and KT3. These functionals are currently used in combination with Pople’s 6-31G(d,p) and 6-311G(d,p); Sauer’s 6-31G-J, 6-311G-J, and aug-cc-pVTZ-J; Dunning’s cc-pVnZ (*n* = D, T, and Q); Jensen’s pcS-n, pcSseg-n, and pcJ-n (*n* = 1−4); and, for relativistic calculations, Dyall’s dyall.vXz, dyall.cvXz, dyall.avXz, and dyall.acvXz (*X* = 2−4) basis sets.

For monosaccharides, calculation and stereochemical applications of the NMR parameters were historically limited to the implementation of four vicinal proton-proton coupling constants, namely, ^3^*J*(H-1,H-2), ^3^*J*(H-2,H-3), ^3^*J*(H-3,H-4), and ^3^*J*(H-4,H-5), which are most critical to the conformation of the hexapyranosyl moiety. Those couplings for a classical ^4^C_1_ conformation of carbohydrates are essentially large (8 Hz or larger), which indicates on the fact that all coupled protons are in the axial positions of the pyranose ring characterized by their mutual transoidal orientations. Such a behavior markedly contrasts to that of α-D-idohexopyranose, where corresponding ^3^*J*_H,H_ couplings range from about 5 to 8 Hz, which suggests a highly preferable alternative ^1^C_4_ conformation, as compared to a classical ^4^C_1_ one.

Geminal, vicinal, and long-range ^1^H-^1^H spin-spin couplings provide marked structural and conformational dependencies, which can be used in the conformational analysis of *O*-glycosidic linkages in mono-, di-, and polysaccharides. The values of ^2^*J*_H,H_ couplings are affected by the conformation of the C–O bond adjacent to the exocyclic hydroxymethyl carbon, while ^3^*J*_H,H_ are strongly influenced by the relative arrangement of the hydroxyl groups attached to carbons bearing coupled hydrogens. In some pyranosyl ring conformations, longer-range four-bond ^1^H-^1^H coupling constants depend on the geometry of the corresponding coupling path, as well as on the relative arrangement of hydroxyl groups along the coupling pathway. This trend provides an additional guiding thread to the conformational behavior of the pyranose rings. In particular, established trends in the computational carbon-hydrogen coupling constants of pyranoses suggest their important application to the conformational studies of the glycosidic C‒O torsion angles of saccharides.

One-bond and geminal carbon-carbon coupling constants are sensitive to the presence and orientation of the hydroxyl groups attached to the coupled carbons depending on the rotation around central C–C bond, demonstrating their well-defined Karplus-type dependence. The latter originates in the rotation around the C–O bonds. On the other hand, the value of ^2^*J*_CCC_ in a HO–C–C(OH)–C–OH coupling pathway in aldopyranosyl rings depends markedly on the relative orientation of the hydroxyl groups attached to the terminal coupled carbons (configurational effect) and on the orientation of a hydroxyl substituent attached to the central carbon (conformational effect).

Rotation of the hydroxyl groups exercises an appreciable effect on ^1^*J*(C-1,C-2) coupling constants in the furanose, pyranose, and septanose forms of carbohydrates. Undoubtedly, those dependences originate in the well-known oxygen lone pair effect on ^1^*J*_C,C_, which should be taken into account in the conformational analysis of the cyclic forms of carbohydrates based on the stereospecificity of carbon-carbon coupling constants involving anomeric carbon.

The DFT calculations provide insight into the formation of intra- and intermolecular hydrogen bonds and ionic interactions. In particular, the H-bond network of a polysaccharide chain consists of intra- and interresidual intramolecular hydrogen bonds that affect the overall three-dimensional molecular structure of oligosaccharides in liquid and solid states exemplified with the α-(1→1′), β-(1→4′), and α-(1→4′)-linked disaccharides.

Intermolecular hydrogen bonding has a major influence on the NMR parameters in isotropic and crystalline states of mono-, di-, and polysaccharides. Intra- and intermolecular hydrogen bonding plays an essential role in the determining the chemical shielding principal components in the molecular frame axes while chemical shift surfaces, defining the conformational potential basin, contain information on conformational behavior of oligo- and polysaccharides and distribution of their conformations in glassy and amorphous states.

## Figures and Tables

**Table 1:** Comparison of calculated and experimental values of δ_H_ (ppm), δ_C_ (ppm), and *J*_H,H_ (Hz) of the α and β anomers of D-glucopyranose. Compiled from Roslund et al. [111].

NMR Parameter	α-D-Glucopyranose		β-D-Glucopyranose	
	Calc.	Exp.	Calc.	Exp.
H-1	5.301	5.214	4.586	4.627
H-2	3.273	3.516	3.152	3.226
H-3	3.653	3.696	3.478	3.469
H-4	3.589	3.393	3.614	3.385
H-5	3.907	3.817	3.422	3.447
H-6a	3.913	3.823	3.966	3.879
H-6b	3.768	3.745	3.753	3.704
MAD		0.110		0.073
C-1	104.74	92.77	108.59	96.59
C-2	82.14	72.15	85.01	74.81
C-3	83.96	73.43	86.00	76.43
C-4	79.55	70.32	79.23	70.27
C-5	81.89	72.10	86.58	76.61
C-6	70.10	61.27	70.18	61.42
MAD		10.06		9.91
^3^*J*(H-1,H-2)	4.59	3.80	7.75	7.97
^4^*J*(H-1,H-3)	−0.45	−0.39	0.00	
^4^*J*(H-1,H-5)	-0.80	−0.53	−0.01	
^3^*J*(H-2,H-3)	9.83	9.84	9.58	9.46
^3^*J*(H-3,H-4)	9.26	9.17	9.11	9.17
^3^*J*(H-4,H-5)	10.06	9.99	9.74	9.92
^3^*J*(H-5,H-6a)	4.00	2.31	3.84	2.27
^3^*J*(H-5,H-6b)	6.23	5.40	7.49	5.95
^3^*J*(H-6a,H-6b)	−13.01	−12.37	−12.99	−12.30
MAD		0.49		0.62

**Table 2:** ^13^C-NMR isotropic chemical shifts of α-D-galactose (**12**) calculated using the PBE and KT3 functionals. Compiled from Kibalchenko et al. [117].

Atom	PBE		KT3		Exp.
	Model A	Model B	Model A	Model B	
C-1	95.0	96.5	94.5	96.1	92.4
C-2	70.3	69.2	69.9	68.9	70.4
C-3	70.0	69.5	69.9	69.2	70.6
C-4	70.4	70.0	71.2	71.0	70.1
C-5	68.8	68.9	68.1	69.0	68.8
C-6	57.6	56.9	57.7	57.0	58.8
MAE	0.9	1.4	1.0	1.6	

**Table 3:** One-bond spin-spin coupling constants ^13^C-^13^C (Hz) of the α and β forms of D-hexopyranoses calculated at the SCPT INDO level as compared to experiment (given in parenthesis). For structures, see Scheme 4. Compiled from the PhD Thesis by Danilova [126].

Cmpd.	Orientation of OH at C^1^ and C^2^	^1^*J*(C-1,C-2)	Cmpd.	Orientation of OH at C^1^ and C^2^	^1^*J*(C-1,C-2)
α-D-allo-4C1	ae	44.8 (45.4)	α-D-gulo-4C1	ae	47.7 (45.9)
α-D-allo-1C4	ea	43.4	α-D-gulo-1C4	ea	43.8
β-D-allo-4C1	ee	47.9 (47.3)	β-D-gulo-4C1	ee	47.6 (47.7)
β-D-allo-1C4	aa	46.2	β-D-gulo-1C4	aa	46.6
α-D-altro-4C1	ae	45.2 (46.2)	α-D-ido-4C1	ae	48.7 (46.2)
α-D-altro-1C4	ea	45.9 (46.2)	α-D-ido-1C4	ea	45.8 (46.2)
β-D-altro-4C1	ee	45.3 (43.9)	β-D-ido-4C1	ee	45.9 (43.8)
β-D-altro-1C4	aa	45.8	β-D-ido-1C4	aa	45.6
α-D-gluco-4C1	ae	45.8 (46.2)	α-D-galacto-4C1	ae	46.0 (46.0)
α-D-gluco-1C4	ea	44.4	α-D-galacto-1C4	ea	44.0
β-D-gluco-4C1	ee	46.2 (46.0)	β-D-galacto-4C1	ee	46.1 (45.9)
β-D-gluco-1C4	aa	47.5	β-D-galacto-1C4	aa	48.2
α-D-manno-4C1	ae	46.9 (46.7)	α-D-talo-4C1	ae	47.5 (46.5)
α-D-manno-1C4	ea	47.2	α-D-talo-1C4	ea	47.5
β-D-manno-4C1	ee	44.2 (42.7)	β-D-talo-4C1	ee	44.8 (42.3)
β-D-manno-1C4	aa	46.6	β-D-talo-1C4	aa	44.7

**Table 4:** Computed averaged vicinal proton-proton coupling constants (Hz) of heparin tetrasaccharide as compared to experiment. Compiled from Hricovini [147].

Residue ^(a)^	Coupled Nuclei	Calculated ^3^*J*_HH_ (Conformationally Averaged)	Exp.
GlcNS,6S_NR_	H-1,H-2	3.9	3.5
	H-2,H-3	10.0	10.3
	H-3,H-4	9.1	9.7
	H-4,H-5	9.9	9.7
IdoA2S_NR_	H-1,H-2	2.1	2.9
	H-2,H-3	3.2	5.4
	H-3,H-4	3.0	3.8
	H-4,H-5	2.8	2.7
GlcNS,6S_R_	H-1,H-2	3.1	3.5
	H-2,H-3	10.9	10.3
	H-3,H-4	9.9	9.2
	H-4,H-5	9.5	9.2
IdoA2S_R_	H-1,H-2	3.0	2.9
	H-2,H-3	5.3	5.3
	H-3,H-4	3.5	3.9
	H-4,H-5	3.0	2.7

^(a)^ For the designation of the residues, see original publication.

**Table 5:** Computed averaged vicinal proton-proton coupling constants (Hz) of heparin pentasaccharide as compared to experiment. Compiled from Hricovini [148].

Residue ^(a)^	Conformation	Coupled Nuclei	Calculated ^3^*J*_HH_ (Conformationally Averaged)	Exp.
GlcN,6S_NR_	^4^ *C*_1_	H-1,H-2	3.2	3.8
		H-2,H-3	10.0	10.3
		H-3,H-4	8.3	9.6
		H-4,H-5	9.7	10.1
GlcA	^4^ *C*_1_	H-1,H-2	8.0	7.99.5
		H-2,H-3	10.9	
		H-3,H-4	8.1	
		H-4,H-5	9.3	
GlcN,3,6S	^4^ *C*_1_	H-1,H-2	4.6	3.5
		H-2,H-3	11.5	10.7
		H-3,H-4	10.4	9.3
		H-4,H-5	9.9	9.9
IdoA2S	^1^*C*_4_/^2^*S*_0_	H-1,H-2	3.2	3.8
		H-2,H-3	7.1	7.2
		H-3,H-4	1.3	2.4
		H-4,H-5	4.7	3.1
GlcN,6S_R_	^4^ *C*_1_	H-1,H-2	4.6	3.6
		H-2,H-3	10.7	10.4
		H-3,H-4	9.6	8.5
		H-4,H-5	9.5	10.0

^(a)^ For the designation of the residues, see original publication.

**Table 6:** Statistical parameters of the least-squares correlation of ^13^C-NMR chemical shift tensors of 65 saccharides calculated with using different functionals and basis sets versus experiment. Compiled from Sefzik [151].

Method	Basis Set	Slope	Intercept	RMSD
RHF	cc-pVDZ	−0.956	205.2	4.14
	cc-pVTZ	−0.966	196.0	3.73
HF+Becke	cc-pVDZ	−1.091	192.5	5.02
	cc-pVTZ	−1.150	183.7	5.08
HF+Slater	cc-pVDZ	−1.144	200.1	5.38
	cc-pVTZ	−1.203	189.7	5.49
BLYP	cc-pVDZ	−1.108	194.0	5.05
	cc-pVTZ	−1.171	185.0	5.03
B3LYP	cc-pVDZ	−1.085	197.6	4.70
	cc-pVTZ	−1.133	188.2	4.60
B3P86	cc-pVDZ	−1.091	201.1	4.51
	cc-pVTZ	−1.130	192.0	4.31
BVWn	cc-pVDZ	−1.099	193.0	4.81
	cc-pVTZ	−1.156	184.0	4.76
SVWN	cc-pVDZ	−1.155	201.1	4.98
	cc-pVTZ	−1.211	190.4	4.99
mPW1PW91	cc-pVDZ	−1.081	201.8	4.42
	cc-pVTZ	−1.112	193.8	5.58

**Table 7:** Calculated and experimental (in parenthesis) ^13^C-NMR chemical shifts of different oligosaccharides in solid state. Compiled from Sergeyev and Moyna [153].

Oligosaccharide	φ	ψ	C-1	C-1′ (C-4′)
Trehalose	−49.0 (−51.9)	−49.0 (−51.9)	93.4 (93.0)	93.4 (93.0)
Cellobiose	40.6 (41.2)	−16.4 (−12.9)	104.8 (104.6)	85.3 (85.0)
Cellotetraose	25.8 (27.3)	−28.9 (−25.5)	107.2 (107.4)	88.7 (89.7)
Cellohexaose	31.3	−35.0	106.2 (107.1)	87.9 (89.3)
Cellulose II	31.3 (21.9)	−35.0 (−29.0)	106.2 (107.1)	87.9 (88.9)
Amilose V_6_	−7.4 (−9.6)	−22.2 (−9.0)	102.3 (103.8)	82.0 (82.4)

## Data Availability

Not applicable.

## Abbreviations

AM1	Austin Model 1
B3	Three-parameter hybrid functional of Becke
BH	Becke’s “half and half” HF/DFT hybrid exchange functional
CBS	Complete Basis Set
CC3	Coupled Cluster Iterative Triples (model)
CCSD	Coupled Cluster Singles and Doubles
CCSD(T)	Coupled Cluster Singles and Doubles with Perturbative Triples Corrections
CCSDT	Coupled Cluster Single-Double and Triple corrections
CMAE	Corrected Mean Absolute Error
CPCM	Conductor-like Polarizable Continuum Model
CP/MAS	Cross-Polarization/Magic-Angle Spinning
DFT	Density Functional Theory
EFG	Electic Field Gradient
Fuc	Fucose
Gal	Galactose
GlcNAc	N-acetyl glucosime
GIAO	Gauge including atomic orbitals
GIPAW	Gauge Including Projector Augmented Waves
HRPA(D)	Highest Random Phase Approximation Doubles (model)
IEF-PCM:	Integral Equation Formalism Polarizable Continuum Model
KT2	Generalized gradient exchange-correlation functional of Keal and Tozer
KT3	Generalized gradient exchange-correlation functional of Keal and Tozer with the gradient-corected exchange and correlation terms
LDBS	Locally Dense Basis Set
LYP	Lee, Yang, and Parr (correlation functional)
MAD	Mean Absolute Deviations
MAE	Mean Absolute Error
MAS	Magic-Angle Spinning
MD	Molecular Dynamics
MM	Molecular Mechanics
MP2	Møller-Plesset perturbation theory (second-order)
Neu5Ac	N-acetyl neuramic acid
NMR	Nuclear Magnetic Resonance
OLYP	Handy and Cohen’s hybrid functional in combination with Lee, Yang and Parr’s correlation functional
OPBE	Handy and Cohen’s hybrid functional in combination with the generalized gradient functional of Perdew, Burke and Ernzerhof
OPTX	Handy and Cohen’s hybrid functional
OPW91	Handy and Cohen’s hybrid functional in combination with the generalized gradient functional of Perdew and Wang
PBE0	the generalized gradient functional of Perdew, Burke and Ernzerhof with a predetermined amount of exact exchange
PBEc	Perdew, Burke and Ernzerhof correlation functional
PW91	Generalized gradient functional of Perdew and Wang
QM/MM	Quantum Mechanics/Molecular Mechanics (approach)
RPA	Random Phase Approximation
RPA(D)	Random Phase Approximation Doubles (model)
RHF	Restricted Hartree-Fock
SOPPA(CC2)	Second-Order Polarization Propagator Approach in combination with the second-order approximate Coupled Cluster to second order
SOPPA(CCSD)	Second-Order Polarization Propagator Approach in combination with Coupled Cluster Singles and Doubles
SOPPA(MP2)	Second-Order Polarization Propagator Approach within the second-order Møller-Plesset perturbation theory
SSM	Supermolecular Solvation Model
ZPVC	Zero-Point Vibrational Correction

## References

[1] Klepach T., Zhao H., Hu X., Zhang W., Stenutz R., Hadad M.J., Carmichael I., Serianni A.S., Lütteke T., Frank M. (2015). Informing Saccharide Structural NMR Studies with Density Functional Theory Calculations. Glycoinformatics, Methods in Molecular Biology.

[2] Hadad M.J., Zhang W., Turney T., Sernau L., Wang X., Woods R.J., Incandela A., Surjancev I., Wang A., Yoon M.-K., Kato K., Peters T. (2017). NMR Spin-Couplings in Saccharides: Relationships Between Structure, Conformation and the Magnitudes of J_HH_, J_CH_ and J_CC_ Values. NMR in Glycoscience and Glycotechnology.

[3] Serianni A.S., Barker R., Buncel E., Jones J.R. (1987). Synthetic Approaches to Carbohydrates Enriched with Stable Isotopes of Carbon, Hydrogen and Oxygen, Isotopes in the Physical and Biomedical Sciences, Vol. I, Labelled Compounds (Part, A).

[4] Serianni A.S., Allen H.J., Kisailus E.C. (1992). Nuclear Magnetic Resonance Approaches to Oligosaccharide Structure Elucidation, Glycoconjugates: Composition, Structure and Function.

[5] Serianni A.S., Trewhella J., Cross T.A., Unkefer C.J. (1994). Stable Isotope Labeled Carbohydrates and Nucleosides: Synthesis and Applications in Chemistry and Biology, Stable Isotope Applications in Biomolecular Structure and Mechanisms.

[6] Serianni A.S., Hecht S.M. (1999). Carbohydrate Structure, Conformation and Reactivity: NMR Studies with Stable Isotopes, Bio-Organic Chemistry: Carbohydrates.

[7] Toukach F.V., Ananikov V.P. (2013). Recent advances in computational predictions of NMR parameters for the structure elucidation of carbohydrates: Methods and limitations. Chem. Soc. Rev..

[8] Sauer S.P.A. (2012). Molecular Electromagnetism. A Computational Chemistry Approach.

[9] Krivdin L.B. (2019). Computational ^1^H-NMR: Part 1. Theoretical background. Magn. Reson. Chem..

[10] Krivdin L.B. (2020). Computational ^1^H-NMR: Part 2. Chemical applications. Magn. Reson. Chem..

[11] Krivdin L.B. (2020). Computational ^1^H-NMR: Part 3. Biochemical studies. Magn. Reson. Chem..

[12] Krivdin L.B. (2019). Computational protocols for calculating ^13^C-NMR chemical shifts. Prog. NMR Spectrosc..

[13] Krivdin L.B. (2018). Theoretical calculations of carbon-hydrogen spin-spin coupling constants. Prog. NMR Spectrosc..

[14] Krivdin L.B. (2018). Carbon-carbon spin-spin coupling constants: Practical applications of theoretical calculations. Prog. NMR Spectrosc..

[15] Helgaker T., Jaszuński M., Ruud K. (1999). Ab initio methods for the calculation of NMR shielding and indirect spin-spin coupling constants. Chem. Rev..

[16] Helgaker T., Jaszuński M., Pecul M. (2008). The quantum-chemical calculation of NMR indirect spin-spin coupling constants. Prog. NMR Spectrosc..

[17] Helgaker T., Coriani S., Jørgensen P., Kristensen K., Olsen J., Ruud K. (2012). Recent advances in wave function-based methods of molecular-property calculations. Chem. Rev..

[18] Cremer D., Grafenstein J. (2007). Calculation and analysis of NMR spin-spin coupling constants. Phys. Chem. Chem. Phys..

[19] Vaara J. (2007). Theory and computation of nuclear magnetic resonance parameters. Phys. Chem. Chem. Phys..

[20] Aucar G.A., Romero R.H., Maldonado A.F. (2010). Polarization propagators: A powerful theoretical tool for a deeper understanding of NMR spectroscopic parameters. Int. Rev. Phys. Chem..

[21] Bühl M., van Mourik T. (2011). NMR spectroscopy: Quantum-chemical calculations. WIREs Comput. Mol. Sci..

[22] Rusakov Y.Y., Krivdin L.B. (2013). Modern quantum chemical methods for calculating spin-spin coupling constants: Theoretical basis and structural applications in chemistry. Russ. Chem. Rev..

[23] Rusakova I.L., Rusakov Y.Y., Krivdin L.B. (2016). Theoretical grounds of relativistic methods for calculation of spin-spin coupling constants in nuclear magnetic resonance spectra. Russ. Chem. Rev..

[24] Mulder F.A.A., Filatov M. (2010). NMR chemical shift data and ab initio shielding calculations: Emerging tools for protein structure determination. Chem. Soc. Rev..

[25] Krivdin L.B., Contreras R.H. (2007). Recent advances in theoretical calculations of indirect spin-spin coupling constants. Ann. Rep. NMR Spectrosc..

[26] Navarro-Vázquez A. (2017). State of the art and perspectives in the application of quantum chemical prediction of ^1^H and ^13^C chemical shifts and scalar couplings for structural elucidation of organic compounds. Magn. Reson. Chem..

[27] Krivdin L.B. (2017). Calculation of ^15^N-NMR chemical shifts: Recent advances and perspectives. Prog. NMR Spectrosc..

[28] Krivdin L.B. (2020). Computational aspects of ^19^F-NMR. Russ. Chem. Rev..

[29] Krivdin L.B. (2020). Computational liquid-phase and solid-state ^29^Si-NMR. Russ. Chem. Rev..

[30] Krivdin L.B. (2020). Recent advances in computational ^31^P-NMR: Part 1. Chemical shifts. Magn. Reson. Chem..

[31] Krivdin L.B. (2020). Recent advances in computational ^31^P-NMR: Part 2. Spin-spin coupling constants. Magn. Reson. Chem..

[32] Krivdin L.B., Rusakov Y.Y. (2014). Structural and stereochemical applications of computational NMR using ^29^Si-^1^H and ^77^Se-^1^H indirect spin-spin coupling constants. Enc. Magn. Reson..

[33] Krivdin L.B. (2020). Recent advances in computational liquid-phase ^77^Se-NMR. Russ. Chem. Rev..

[34] Rusakova I.L., Rusakov Y.Y. (2021). Quantum chemical calculations of ^77^Se and ^125^Te nuclear magnetic resonance spectral parameters and their structural applications. Magn. Reson. Chem..

[35] Rusakova I.L., Krivdin L.B. (2018). Relativistic effects in the NMR spectra of compounds containing heavy chalcogens. Mendeleev Commun..

[36] Krivdin L.B. (2020). Computational NMR of heavy nuclei involving ^109^Ag, ^113^Cd, ^119^Sn, ^125^Te, ^195^Pt, ^199^Hg, ^205^Tl, and ^207^Pb. Russ. Chem. Rev..

[37] Frisch M.J., Trucks G.W., Schlegel H.B., Scuseria G.E., Robb M.A., Cheeseman J.R., Scalmani G., Barone V., Mennucci B. (2009). GAUSSIAN 09, Revision, C.01.

[38] Auer A.A., Gauss J. (2001). Triple excitation effects in coupled-cluster calculations of indirect spin-spin coupling constants. J. Chem. Phys..

[39] Faber R., Sauer S.P.A., Gauss J. (2016). The importance of triples contributions to NMR spin-spin coupling constants computed at the CC3 and CCSDT levels. J. Chem. Theory Comp..

[40] Sauer S.P.A. (1997). Second order polarization propagator approximation with coupled cluster singles and doubles amplitudes—SOPPA(CCSD): The polarizability and hyperpolarizability of Li^-^. J. Phys. B At. Mol. Opt. Phys..

[41] Enevoldsen T., Oddershede J., Sauer S.P.A. (1998). Correlated calculations of indirect nuclear spin-spin coupling constants using second order polarization propagator approximations: SOPPA and SOPPA(CCSD). Theor. Chem. Acc..

[42] Kjær H., Sauer S.P.A., Kongsted J. (2010). Benchmarking NMR indirect nuclear spin-spin coupling constants: SOPPA, SOPPA(CC2) and SOPPA(CCSD) versus CCSD. J. Chem. Phys..

[43] Kjær H., Sauer S.P.A., Kongsted J., Rusakov Y.Y., Krivdin L.B. (2011). Benchmarking SOPPA(CC2) for the calculation of indirect nuclear spin-spin coupling constants: Carbocycles. Chem. Phys..

[44] Schnack-Petersen A.C., Haase P.A.B., Faber R., Provasi P.F., Sauer S.P.A. (2018). RPA(D) and HRPA(D): Two new models for calculations of NMR indirect nuclear spin-spin coupling constants. J. Comp. Chem..

[45] Møller C.H.S., Sauer S.P.A. (2020). RPA(D) and HRPA(D): Calculation of carbon-carbon spin-spin coupling constants for saturated cycloalkanes. Mol. Phys..

[46] Becke A.D. (1993). Density functional thermochemistry. III. The role of exact exchange. J. Chem. Phys..

[47] Lee C., Yang W., Parr R.G. (1988). Development of the Colle-Salvetti correlation-energy formula into a functional of the electron density. Phys. Rev. B.

[48] Becke A.D. (1993). A new mixing of Hartree-Fock and local density-functional theories. J. Chem. Phys..

[49] Handy N.C., Cohen A.J. (2001). Left-right correlation energy. Mol. Phys..

[50] Perdew J.P., Burke K., Ernzerhof M. (1996). Generalized gradient approximation made simple. Phys. Rev. Lett..

[51] Burke K., Perdew J.P., Wang Y., Dobson J.F. (1998). Derivation of a Generalized Gradient Approximation: The PW91 Density Functional. Electronic Density Functional Theory.

[52] Adamo C., Barone V. (1999). Toward reliable density functional methods without adjustable parameters: The PBE0 model. J. Chem. Phys..

[53] Keal T.W., Tozer D.J. (2003). The exchange-correlation potential in Kohn–Sham nuclear magnetic resonance shielding calculations. J. Chem. Phys..

[54] Keal T.W., Tozer D.J. (2004). A semiempirical generalized gradient approximation exchange-correlation functional. J. Chem. Phys..

[55] Basis Set Exchange Database. https://www.basissetexchange.org/.

[56] Iron M.A. (2017). Evaluation of the factors impacting the accuracy of ^13^C-NMR chemical shift predictions using density functional theory—the advantage of long-range corrected functionals. J. Chem. Theory Comp..

[57] Smith S.G., Goodman J.M. (2010). Assigning stereochemistry to single diastereoisomers by GIAO NMR calculation: The DP4 Probability. J. Am. Chem. Soc..

[58] Grimblat N., Zanardi M.M., Sarotti A.M. (2015). Beyond DP4: An improved probability for the stereochemical assignment of isomeric compounds using quantum chemical calculations of NMR shifts. J. Org. Chem..

[59] Semenov V.A., Krivdin L.B. (2021). Computational ^1^H and ^13^C-NMR of the trimeric monoterpenoid indole alkaloid strychnohexamine: Selected spectral updates. Magn. Reson. Chem..

[60] Chesnut D.B., Moore K.D. (1989). Locally dense basis sets for chemical shift calculations. J. Comp. Chem..

[61] Chesnut D.B., Rusiloski B.E., Moore K.D., Egolf D.A. (1993). Use of locally dense basis sets for nuclear magnetic resonance shielding calculations. J. Comp. Chem..

[62] Chesnut D.B., Byrd E.F.C. (1996). The use of locally dense basis sets in correlated NMR chemical shielding calculations. Chem. Phys..

[63] Provasi P.F., Aucar G.A., Sauer S.P.A. (2000). The use of locally dense basis sets in the calculation of indirect nuclear spin-spin coupling constants: The vicinal coupling constants in H_3_C-CH_2_X (X. = H., F., Cl, Br, I). J. Chem. Phys..

[64] Sanchez M., Provasi P.F., Aucar G.A., Sauer S.P.A. (2005). On the usage of locally dense basis sets in the calculation of nmr indirect nuclear spin-spin coupling constants: Vicinal fluorine-fluorine couplings. Adv. Quantum Chem..

[65] Kirby R.A., Hansen A.E. (1996). Study of locally dense and locally saturated basis sets in localized molecular orbital calculations of nuclear shielding: Ab initio LORG calculations for ^13^C and ^17^O in norbornenone. Int. J. Quantum Chem..

[66] Chesnut D.B., Phung C.G. (1991). Ab initio determination of chemical shielding in a model dipeptide. Chem. Phys. Lett..

[67] Semenov V.A., Krivdin L.B. (2020). DFT computational schemes for ^1^H and ^13^C-NMR chemical shifts of natural products, exemplified by strychnine. Magn. Reson. Chem..

[68] Rusakov Y.Y., Krivdin L.B. (2013). One-bond ^29^Si-^1^H spin-spin coupling constants in the series of halosilanes: Benchmark SOPPA and DFT calculations, relativistic effects, and vibrational corrections. Magn. Reson. Chem..

[69] Rusakov Y.Y., Krivdin L.B., Osterstrom F.F., Sauer S.P.A., Potapov V.A., Amosova S.V. (2013). First example of a high-level correlated calculation of the indirect spin-spin coupling constants involving tellurium: Tellurophene and divinyl telluride. Phys. Chem. Chem. Phys..

[70] Rusakov Y.Y., Krivdin L.B., Nosova V.M., Kisin A.V., Lakhtin V.G. (2012). Structural trends of ^29^Si-^1^H spin-spin coupling constants across double bond. Magn. Reson. Chem..

[71] Rusakov Y.Y., Krivdin L.B., Penzik M.V., Potapov V.A., Amosova S.V. (2012). Open-chain unsaturated selanyl sulfides: Stereochemical structure and stereochemical behavior of their ^77^Se-^1^H spin-spin coupling constants. Magn. Reson. Chem..

[72] Rusakov Y.Y., Krivdin L.B. (2012). Stereochemical behavior of ^2^*J*(Se,H) and ^3^*J*(Se,H) spin-spin coupling constants across sp^3^ carbons: A theoretical scrutiny. Magn. Reson. Chem..

[73] Rusakov Y.Y., Krivdin L.B., Kumar A.A., Szilagyi L., Kover K.E. (2012). Resonance assignments of diastereotopic CH_2_ protons in the anomeric side-chain of selenoglycosides by means of ^2^*J*(Se,H) spin-spin coupling constants. Magn. Reson. Chem..

[74] Rusakov Y.Y., Krivdin L.B., Nosova V.M., Kisin A.V. (2012). Benchmark calculations of ^29^Si-^1^H spin-spin coupling constants across double bond. Magn. Reson. Chem..

[75] Rusakov Y.Y., Krivdin L.B., Papernaya L.K., Shatrova A.A. (2012). Stereochemical behavior of ^77^Se-^1^H spin-spincoupling constants in pyrazolyl-1,3-diselenanes and 1,2-diselenolane. Magn. Reson. Chem..

[76] Rusakov Y.Y., Krivdin L.B., Orlov N.V., Ananikov V.P. (2011). Stereochemical study of the sterically crowded phenylselanylalkenes by means of ^77^Se-^1^H spin-spin coupling constants. Magn. Reson. Chem..

[77] Rusakov Y.Y., Krivdin L.B., Potapov V.A., Penzik M.V., Amosova S.V. (2011). Conformational analysis and diastereotopic assignments in the series of seleniumcontaining heterocycles by means of ^77^Se-^1^H spin-spin coupling constants: A combined theoretical and experimental study. Magn. Reson. Chem..

[78] Rusakov Y.Y., Krivdin L.B., Sauer S.P.A., Levanova E.P., Levkovskaya G.G. (2010). Structural trends of ^77^Se-^1^H spin-spin coupling constants and conformational behavior of 2-substituted selenophenes. Magn. Reson. Chem..

[79] Rusakov Y.Y., Krivdin L.B., Istomina N.V., Potapov V.M., Amosova S.V. (2008). Divinyl selenide: Conformational study and stereochemical behavior of its ^77^Se-^1^H spin-spin coupling constants. Magn. Reson. Chem..

[80] Semenov V.A., Samultsev D.O., Krivdin L.B. (2020). The ^1^H and ^13^C-NMR chemical shifts of *Strychnos* alkaloids revisited at the DFT level. Magn. Reson. Chem..

[81] Semenov V.A., Samultsev D.O., Krivdin L.B. (2020). ^1^H and ^13^C-NMR spectra of *Strychnos* alkaloids: Selected NMR updates. Int. J. Quant. Chem..

[82] Semenov V.A., Samultsev D.O., Krivdin L.B. (2019). DFT computational schemes for ^15^N-NMR chemical shifts of the condensed nitrogen—Containing heterocycles. Magn. Reson. Chem..

[83] Semenov V.A., Samultsev D.O., Krivdin L.B. (2018). Substitution effects in the ^15^N-NMR chemical shifts of heterocyclic azines evaluated at the GIAO—DFT level. Magn. Reson. Chem..

[84] Semenov V.A., Samultsev D.O., Krivdin L.B. (2018). GIAO—DFT calculation of ^15^N-NMR chemical shifts of Schiff bases: Accuracy factors and protonation effects. Magn. Reson. Chem..

[85] Samultsev D.O., Semenov V.A., Krivdin L.B. (2017). On the accuracy factors and computational cost of the GIAO–DFT calculation of ^15^N-NMR chemical shifts of amides. Magn. Reson. Chem..

[86] Mennucci B., Tomasi J. (1997). Continuum solvation models: A new approach to the problem of solute’s charge distribution and cavity boundaries. J. Chem. Phys..

[87] Cancès E., Mennucci B., Tomasi J. (1997). A new integral equation formalism for the polarizable continuum model: Theoretical background and applications to isotropic and anisotropic dielectrics. J. Chem. Phys..

[88] Mennucci B., Cancès E., Tomasi J. (1997). Evaluation of solvent effects in isotropic and anisotropic dielectrics and in ionic solutions with a unified integral equation method:  theoretical bases, computational implementation, and numerical applications. J. Phys. Chem. B.

[89] Tomasi J., Mennucci B., Cancès E. (1999). The IEF version of the PCM solvation method: An overview of a new method addressed to study molecular solutes at the QM ab initio level. THEOCHEM.

[90] Tomasi J., Mennucci B., Cammi R. (2005). Quantum mechanical continuum solvation models. Chem. Rev..

[91] Klamt A., Schüürmann G. (1993). COSMO: A new approach to dielectric screening in solvents with explicit expressions for the screening energy and its gradient. J. Chem. Soc. Perkin Trans. 2.

[92] Andzelm J., Kölmel C., Klamt A. (1995). Incorporation of solvent effects into density functional calculations of molecular energies and geometries. J. Chem. Phys..

[93] Barone V., Cossi M. (1998). Quantum calculation of molecular energies and energy gradients in solution by a conductor solvent model. J. Phys. Chem. A.

[94] Cossi M., Rega N., Scalmani G., Barone V. (2003). Energies, structures, and electronic properties of molecules in solution with the C-PCM solvation model. J. Comput. Chem..

[95] Takano Y., Houk K.N. (2005). Benchmarking the conductor-like polarizable continuum model (CPCM) for aqueous solvation free energies of neutral and ionic organic molecules. J. Chem. Theory Comput..

[96] Semenov V.A., Samultsev D.O., Krivdin L.B. (2014). Solvent effects in the GIAO-DFT calculations of the ^15^N-NMR chemical shifts of azoles and azines. Magn. Reson. Chem..

[97] Caputo M.C., Provasi P.F., Sauer S.P.A. (2018). The role of explicit solvent molecules in the calculation of NMR chemical shifts of glycine in water. Theor. Chem. Acc..

[98] Lacerda E.G., Kamounah F.S., Coutinho K., Sauer S.P.A., Hansen P.E., Hammerich O. (2019). Computational Prediction of ^1^H and ^13^C-NMR Chemical Shifts for Protonated Alkylpyrroles: Electron Correlation and Not Solvation is the Salvation. ChemPhysChem.

[99] Møgelhøj A., Aidas K., Mikkelsen K.V., Sauer S.P.A., Kongsted J. (2009). Prediction of spin-spin coupling constants in solution based on combined density functional theory/molecular mechanics. J. Chem. Phys..

[100] Ruden T.A., Lutnæs O.B., Helgaker T., Ruud K. (2003). Vibrational corrections to indirect nuclear spin-spin coupling constants calculated by density-functional theory. J. Chem. Phys..

[101] Lutnæs O.B., Ruden T.A., Helgaker T. (2004). The performance of hybrid density functional theory for the calculation of indirect nuclear spin-spin coupling constants in substituted hydrocarbons. Magn. Reson. Chem..

[102] Barone V. (2005). Anharmonic vibrational properties by a fully automated second-order perturbative approach. J. Chem. Phys..

[103] Irikura K.K. (2007). Experimental vibrational zero-point energies: Diatomic molecules. J. Phys. Chem. Ref. Data.

[104] Faber R., Buczek A., Kupka T., Sauer S.P.A. (2016). On the convergence of zero-point vibrational corrections to nuclear shieldings and shielding anisotropies towards the complete basis set limit in water. Mol. Phys..

[105] Kupka T., Buczek A., Broda M.A., Stachów M., Tarnowski P. (2016). DFT studies on the structural and vibrational properties of polyenes. J. Mol. Model..

[106] Ruden T.A., Ruud K., Kaupp M., Malkin V.G., Buehl M. (2004). Calculation of NMR and EPR Parameters: Theory and Applications.

[107] Faber R., Kaminsky J., Sauer S.P.A., Jackowski K., Jaszuński M. (2016). Rovibrational and temperature effects in theoretical studies of NMR parameters. Gas Phase NMR..

[108] Autschbach J., Contreras R.H. (2013). Relativistic effects on NMR parameters. High Resolution NMR Spectroscopy: Understanding Molecules and Their Electronic Structures.

[109] Xiao Y., Liu W., Autschbach J., Liu W. (2017). Relativistic theories of NMR shielding. Handbook of Relativistic Quantum Chemistry.

[110] Zhu Y., Zajicek J., Serianni A.S. (2001). Acyclic forms of [1-^13^C]aldohexoses in aqueous solution:  quantitation by ^13^C-NMR and deuterium isotope effects on tautomeric equilibria. J. Org. Chem..

[111] Roslund M.U., Tähtinen P., Niemitz M., Sjöholm R. (2008). Complete assignments of the ^1^H and ^13^C chemical shifts and *J*_H,H_ coupling constants in NMR spectra of D-glucopyranose and all D-glucopyranosyl-D-glucopyranosides. Carbohydr. Res..

[112] Da Silva C.O., Mennucci B., Vreven T. (2004). Density functional study of the optical rotation of glucose in aqueous solution. J. Org. Chem..

[113] Zrelov O.Y., Syroeshkin A.V., Uspenskaya E.V., Titorovich O.V., Pleteneva T.V. (2015). Effect of water isotopic composition on galactose mutarotation kinetics. Pharm. Chem. J..

[114] Bagno A., Rastrelli F., Saielli G. (2007). Prediction of the ^1^H and ^13^C-NMR spectra of *α*-D-glucose in water by DFT methods and MD simulations. J. Org. Chem..

[115] Saielli G., Bagno A. (2010). Preferential solvation of glucose and talose in water-acetonitrile mixtures: A molecular dynamics simulation study. Phys. Chem. Chem. Phys..

[116] Kibalchenko M., Lee D., Shao L., Payne M.C., Titman J.J., Yates J.R. (2010). Distinguishing hydrogen bonding networks in *α*-D-galactose using NMR experiments and first principles calculations. Chem. Phys. Lett..

[117] Pickard C.J., Mauri F. (2001). All-electron magnetic response with pseudopotentials: NMR chemical shifts. Phys. Rev. B.

[118] Szeleszczuk Ł., Gubica T., Zimniak A., Pisklak D.M., Dạbrowska K., Cyrański M.K., Kańska M. (2017). The potential for the indirect crystal structure verification of methyl glycosides based on acetates’ parent structures: GIPAW and solid-state NMR approaches. Chem. Phys. Lett..

[119] Kaseman D.C., McKenney M. (2020). Quadrupolar Coupling. https://chem.libretexts.org/@go/page/1827.

[120] Haasnoot C.A.G., de Leeuw F.A.A.M., Altona C. (1980). The relationship between proton-proton NMR coupling constants and substituent electronegativities—I: An empirical generalization of the Karplus equation. Tetrahedron.

[121] Altona C., Ippel J.H., Hoekzema A.J.A.W., Erkelens C., Groesbeek M., Donders L.A. (1989). Relationship between proton-proton NMR coupling constants and substituent electronegativities. V—Empirical substituent constants deduced from ethanes and propanes. Magn. Reson. Chem..

[122] Altona C., Francke R., de Haan R., Ippel J.H., Daalmans G.J., Hoekzema A.J.A.W., van Wijk J. (1994). Empirical group electronegativities for vicinal NMR proton-proton couplings along a C-C bond: Solvent effects and reparameterization of the Haasnoot equation. Magn. Reson. Chem..

[123] Barfield M., Dean A.M., Fallick C.J., Spear R.J., Sternhell S., Westerman P.W. (1975). Conformational dependence and mechanisms for long-range hydrogen-hydrogen coupling constants over four bonds. J. Am. Chem. Soc..

[124] Zhao H., Pan Q., Zhang W., Carmichael I., Serianni A.S. (2007). DFT and NMR studies of ^2^*J*_COH_, ^3^*J*_HCOH_, and ^3^*J*_CCOH_ spin-couplings in saccharides:  C−O torsional bias and H-bonding in aqueous solution. J. Org. Chem..

[125] Serianni A.S., Wu J., Carmichael I. (1995). One-bond ^13^C-^1^H spin-coupling constants in aldofuranosyl rings: Effect of conformation on coupling magnitude. J. Am. Chem. Soc..

[126] Podlasek C.A., Wu J., Stripe W.A., Bondo P.B., Serianni A.S. (1995). [^13^C]-Enriched methyl aldopyranosides: Structural interpretations of ^13^C–^1^H spin-coupling constants and ^1^H chemical shifts. J. Am. Chem. Soc..

[127] Thibaudeau C., Stenutz R., Hertz B., Klepach T., Zhao S., Wu Q., Carmichael I., Serianni A.S. (2004). Correlated C−C and C−O bond conformations in saccharide hydroxymethyl groups:  parametrization and application of redundant ^1^H−^1^H, ^13^C−^1^H, and ^13^C−^13^C-NMR *J.*-couplings. J. Am. Chem. Soc..

[128] Klepach T., Carmichael I., Serianni A.S. (2005). Geminal ^2^*J*_CCH_ spin−spin coupling constants as probes of the *φ* glycosidic torsion angle in oligosaccharides. J. Am. Chem. Soc..

[129] Tafazzoli M., Ghiasi M. (2007). New Karplus equations for ^2^*J*_HH_, ^3^*J*_HH_, ^2^*J*_CH_, ^3^*J*_CH_, ^3^*J*_COCH_, ^3^*J*_CSCH_, and ^3^*J*_CCCH_ in some aldohexopyranoside derivatives as determined using NMR spectroscopy and density functional theory calculations. Carbohydr. Res..

[130] Carmichael I., Chipman D.M., Podlasek C.A., Serianni A.S. (1993). Torsional effects on the one-bond ^13^C-^13^C spin coupling constant in ethylene glycol: Insights into the behavior of ^1^*J*_CC_ in carbohydrates. J. Am. Chem. Soc..

[131] Church T., Carmichael I., Serianni A.S. (1996). Two-bond ^13^C-^13^C spin-coupling constants in carbohydrates: Effect of structure on coupling magnitude and sign. Carbohydr. Res..

[132] Serianni A.S., Bondo P.B., Zajicek J. (1996). Verification of the projection resultant method for two-bond ^13^C-^13^C coupling sign determinations in carbohydrates. J. Magn. Reson. Ser. B.

[133] Zhao H., Carmichael I., Serianni A.S. (2008). Oligosaccharide trans-glycoside ^3^*J*_COCC_ Karplus curves are not equivalent: Effect of internal electronegative substituents. J. Org. Chem..

[134] Bose-Basu B., Klepach T., Bondo G., Bondo P.B., Zhang W., Carmichael I., Serianni A.S. (2007). ^13^C−^13^C-NMR spin-spin coupling constants in saccharides: Structural correlations involving all carbons in aldohexopyranosyl rings. J. Org. Chem..

[135] Stenutz R., Carmichael I., Widmalm G., Serianni A.S. (2002). Hydroxymethyl group conformation in saccharides: Structural dependencies of ^2^*J*_HH_, ^3^*J*_HH_, and ^1^*J*_CH_ spin-spin coupling constants. J. Org. Chem..

[136] Danilova V.A. (2003). Stereochemical Dependences of ^13^C-^13^C Spin-Spin Coupling Constants of Carbohydrates. Ph.D. Thesis.

[137] Danilova V.A., Krivdin L.B. (2003). ^13^C-^13^C Coupling constants in structural studies: XXXIII. Stereochemical study of the pyranose ring. Russ. J. Org. Chem..

[138] Danilova V.A., Krivdin L.B. (2003). ^13^C-^13^C Spin-spin coupling constants in structural studies: XXXV. Stereochemical study of the furanose ring. Russ. J. Org. Chem..

[139] Danilova V.A., Krivdin L.B. (2004). ^13^C-^13^C Spin-spin coupling constants in structural studies: XXXVI. Stereochemical study of the septanose ring. Russ. J. Org. Chem..

[140] Danilova V.A., Istomina N.V., Krivdin L.B. (2004). ^13^C-^13^C Spin-spin coupling constants in structural studies: XXXVII. Rotational conformations of hydroxy groups in pyranose, furanose, and septanose rings. Russ. J. Org. Chem..

[141] Taurian O.E., Contreras R.H., de Kowalewski D.G., Pérez J.E., Tormena C.F. (2007). Lone-pair orientation effect of an *α*-oxygen atom on ^1^*J*_CC_ NMR spin-spin coupling constants in *o*-substituted phenols. Experimental and DFT study. J. Chem. Theory Comput..

[142] Guerrini M., Hricovíni M., Torri G. (2007). Interaction of heparins with fibroblast growth factors: Conformational aspects. Curr. Pharm. Des..

[143] Hricovíni M., Scholtzová E., Bízik F. (2007). B3LYP/6-311++G** study of structure and spin-spin coupling constant in heparin disaccharide. Carbohydr. Res..

[144] Hricovíni M. (2011). Effect of solvent and counterions upon structure and nmr spin-spin coupling constants in heparin disaccharide. J. Phys. Chem. B.

[145] Hricovíni M., Driguez P.-A., Malkina O.L. (2014). NMR and DFT analysis of trisaccharide from heparin repeating sequence. J. Phys. Chem. B.

[146] Rudd T.R., Yates E.A., Hricovíni M. (2009). Spectroscopic and theoretical approaches for the determination of heparin saccharide structure and the study of protein-glycosaminoglycan complexes in solution. Curr. Med. Chem..

[147] Hricovíni M., Hricovíni M. (2018). Solution conformation of heparin tetrasaccharide. DFT analysis of structure and spin-spin coupling constants. Molecules.

[148] Hricovini M. (2015). The solution structure of heparin pentasaccharide: NMR and DFT analysis. J. Phys. Chem. B.

[149] Zhang W., Zhao H., Carmichael I., Serianni A.S. (2009). An NMR investigation of putative interresidue H-bonding in methyl *α*-cellobioside in solution. Carbohydr. Res..

[150] Zhang W., Turney T., Meredith R., Pan Q., Sernau L., Wang X., Hu X., Woods R.J., Carmichael I., Serianni A.S. (2017). Conformational populations of *β*-(1→4) *O*-glycosidic linkages using redundant NMR *J.*-couplings and circular statistics. J. Phys. Chem. B.

[151] Sefzik T.H., Turco D., Iuliucci R.J., Facelli J.C. (2005). Modeling NMR chemical shift: A survey of density functional theory approaches for calculating tensor properties. J. Phys. Chem. A.

[152] Sergeyev I., Moyna G. (2005). Determination of the three-dimensional structure of oligosaccharides in the solid state from experimental ^13^C-NMR data and ab initio chemical shift surfaces. Carbohydr. Res..

[153] Kapla J., Engström O., Stevensson B., Wohlert J., Widmalm G., Maliniak K. (2015). Molecular dynamics simulations and NMR spectroscopy studies of trehalose-lipid bilayer systems. Phys. Chem. Chem. Phys..

[154] Zhang J., Zhang H., Wu J., Zhang J., He J., Xiang J. (2010). NMR spectroscopic studies of cellobiose solvation in EmimAc aimed to understand the dissolution mechanism of cellulose in ionic liquids. Phys. Chem. Chem. Phys..

[155] Esrafili M.D., Elmi F., Hadipour N.L. (2007). Density functional theory investigation of hydrogen bonding effects on the oxygen, nitrogen and hydrogen electric field gradient and chemical shielding tensors of anhydrous chitosan crystalline structure. J. Phys. Chem. A.

[156] Matthews J.F., Himmel M.E., Crowley M.F. (2012). Conversion of cellulose I*α* to I*β* via a high temperature intermediate (I-HT) and other cellulose phase transformations. Cellulose.

[157] Kasat R.B., Wang N.-H.L., Franses E.I. (2007). Effects of backbone and side chain on the molecular environments of chiral cavities in polysaccharide-based biopolymers. Biomacromolecules.

[158] Lefort R., Bordat P., Attilio Cesaro A., Descamps M. (2007). Exploring conformational energy landscape of glassy disaccharides by cross polarization magic angle spinning ^13^C-NMR and numerical simulations. I. Methodological aspects. J. Chem. Phys..

[159] Shao L., Yates J.R., Titman J.J. (2007). Carbon-13 chemical shift tensors of disaccharides: Measurement, computation and assignment. J. Phys. Chem. A.

[160] Clark S.J., Segall M.D., Pickard C.J., Hasnip P.J., Probert M.J., Refson K., Payne M.C. (2005). First principles methods using CASTEP. Z. Kristallogr..

[161] Navarro D.A., Stortz C.A. (2008). DFT/MM modeling of the five-membered ring in 3,6-anhydrogalactose derivatives and its influence on disaccharide adiabatic maps. Carbohyd. Res..

[162] Suzuki S., Horii F., Kurosu H. (2009). Theoretical investigations of ^13^C chemical shifts in glucose, cellobiose, and native cellulose by quantum chemistry calculations. J. Mol. Struct..

[163] Klemm D., Heublein B., Fink H.-P., Bohn A. (2005). Cellulose: Fascinating biopolymer and sustainable raw material. Angew. Chem. Int. Ed..

[164] Tafazzoli M., Ghiasi M. (2009). Structure and conformation of *α*-, *β*- and *γ*-cyclodextrin in solution: Theoretical approaches and experimental validation. Carbohydr. Pol..

[165] Ishida T. (2010). Computational modeling of carbohydrate-recognition process in E-selectin complex: Structural mapping of Sialyl Lewis X onto ab initio QM/MM free energy surface. J. Phys. Chem. B.

